# Alkali Metal Based Triimidosulfite Cages as Versatile Precursors for Single‐Molecule Magnets

**DOI:** 10.1002/chem.202104470

**Published:** 2022-02-10

**Authors:** Daniel Lüert, Christina M. Legendre, Regine Herbst‐Irmer, Dietmar Stalke

**Affiliations:** ^1^ Institut für Anorganische Chemie Georg-August-University Göttingen Tammannstraße 4 37077 Göttingen Germany

**Keywords:** SMM, sodium, sulfur-nitrogen ligands, triimidosulfite, X-ray diffraction

## Abstract

Based on the potassium [{S(*t*BuN)_2_(*t*BuNH)}_2_K_3_(tmeda)‐K_3_{(HN*t*Bu)(N*t*Bu)_2_S}_2_] (**1**) and sodium precursors [S(*t*BuN)_3_(thf)_3_‐Na_3_SNa_3_(thf)_3_(N*t*Bu)_3_S] (**2**), [S(*t*BuN)_3_(thf)_3_Na_3_{(HN*t*Bu)(N*t*Bu)_2_S}] (**3**) and [(tmeda)_3_S‐{Na_3_(N*t*Bu)_3_S}_2_] (**4**) the syntheses and magnetic properties of three mixed metal triimidosulfite based alkali‐lanthanide‐metal‐cages [(*t*BuNH)Dy{K(0.5tmeda)}_2_{(N*t*Bu)_3_S}_2_]_*n*_ (**5**) and [ClLn{Na(thf)}_2_{(N*t*Bu)_3_S}_2_] with Ln=Dy (**6**), Er (**7**) are reported. The corresponding potassium (**1**) and sodium (**2**–**4**) based cages are characterized through XRD and NMR experiments. Preventing lithium chloride co‐complexation led to a significant increase of SMM performance to previously reported sulfur‐nitrogen ligands. The subsequent Dy^III^‐complexes **5** and **6** display slow relaxation of magnetization at zero field, with relaxation barriers *U*=77.0 cm^−1^ for **5**, 512.9 and 316.3 cm^−1^ for **6**, respectively. Significantly, the latter complex **6** also exhibits a butterfly‐shaped hysteresis up to 7 K.

## Graphical abstract

**The potassium and sodium** cages [{S(*t*BuN)_2_(*t*BuNH)}_2_K_3_(tmeda)K_3_{(HN*t*Bu)(N*t*Bu)_2_S}_2_] (**1**), [S(*t*BuN)_3_(thf)_3_Na_3_SNa_3_(thf)_3_(N*t*Bu)_3_S] (**2**) and [S(*t*BuN)_3_(thf)_3_Na_3_{(HN*t*Bu)(N*t*Bu)_2_S}] (**3**) serve as precursors to give f‐metal single‐molecule magnets (SMM). Especially, the sodium precursor **2** represents an ideal candidate and the subsequent dysprosium SMM [ClDy{Na(thf)}_2_{(N*t*Bu)_3_S}_2_] (**6**) displays an energy barrier *U*_eff_ of 513 cm^−1^, a frequency dependency of the dynamic out‐of‐phase susceptibility up to 60 K and a butterfly hysteresis up to 7 K.

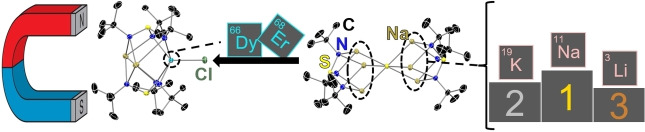

## Introduction

In the last 30 years, the field of single‐molecule magnets (SMMs) has evolved into a sophisticated discipline in chemistry.[^1^, ^2^, ^3^, ^4^, ^5^] Designing the next generation of functional high density data storage and quantum computing application represents the main challenge for this field of research.^[6]^ SMM behavior refers to the property of molecules to preserve the magnetic moment even after switching off the external magnetic field under their blocking temperature.^[7]^ To implement these desired properties for a spin system, one has to fine‐tune the local anisotropy of the metal center that enhances the energy barrier $U_{eff}\;$ to spin reversal. The first approach was the design of giant‐spin clusters with numerous transition metal centers. Despite these systems’ strong magnetic exchange coupling, the opposite occurred, and these systems showed weak or no SMM behavior.^[8]^ An alternative approach, which was no longer based on increasing the total spin quantum number, started in 2003 when 4 f‐metals were first implemented in the field of SMMs and exhibited the highest energy barrier at that time.^[9]^ Here, its intrinsic magnetic anisotropy originates from a first‐order effect due to strong spin‐orbit coupling.^[3]^ Especially Dy^III^ is an ideal candidate because, as a Kramers ion, it implies a bistable ground state that is less affected by the coordination geometry, and this led to impressive results in SMM chemistry.[^4^, ^10^] Therefore, lanthanides seem to be the most promising candidates employed as metal centers in SMM design.[^2^, ^5^, ^11^] The ligand fields affect lanthanide ions to a lesser extent than transition metals: 4f orbitals are deeply buried close to the inner core, while d‐orbitals are further away and primarily affected by the ligand fields. However, it is one of the critical factors chemically addressable through ligand design and tuning, as it impacts magnetic anisotropy. For example, it has been postulated that axial ligand coordination is preferable to stabilize the most magnetic state (*M*_J_=15/2) and destabilize the least magnetic state (*M*_J_=1/2) of Dy^III^.^[19]^ Recent remarkable systems based on dysprosocenium units (a dysprosium trivalent ion sandwiched between two cyclopentadienyl derivatives) further validated this hypothesis, as they outperform any other SMMs, holding operating temperatures above the liquid nitrogen threshold.[^10^, ^20^, ^21^] These systems maximize the linearity of the molecule and ensure the axiality of the magnetic anisotropy of the dysprosium ion.^[22]^ This breakthrough resulted in the design of many similar SMMs, partially neglecting other alternatives and ligand systems, such as p‐block donating ligands or SN ligands.^[23]^

**Scheme 1:**
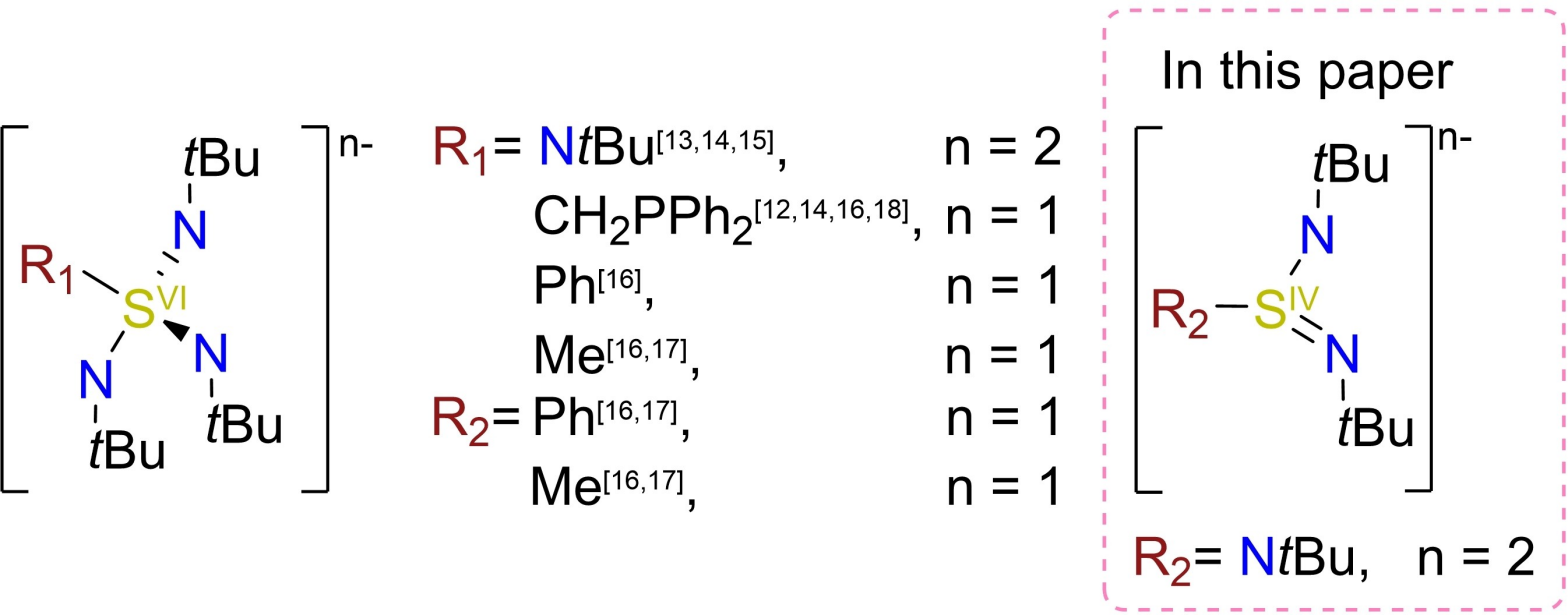
Reported SN ligands already employed in SMMs.[^12^, ^13^, ^14^, ^15^, ^16^, ^17^, ^18^]

The SN ligands,[^24^, ^25^] however, have recently shown their potential for SMM design for the following reasons (Scheme 1):[^12^, ^13^, ^14^, ^15^, ^16^, ^17^, ^18^] First, acute bite angles of the SN motif provide access to interesting and promising geometries.[^12^, ^14^] Further, tuning the SN ligands served as model systems to find the ideal bite angles for tetrahedral Co^II^ SMMs and allowed an increase of linearity in f‐metal SMMs.[^13^, ^16^, ^18^] The tetraimido sulfate ligand family also represents a platform to investigate d‐metal exchange coupling, which was applied for Co^II^ and Mn^II^.^[15]^ Sulfur offers great flexibility in the complex geometry while the harder nitrogen atoms ensure an optimal coordination mode around the metal center, resulting in a great synergy.[^25^, ^27^, ^28^, ^29^] Lanthanide complexes are easily accessible through metathesis reactions and for that purpose, lithium precursors are convenient to use. However, our recent work showed that the successful optimization of the magnetic properties is limited by LiHal co‐complexation.[^13^, ^14^, ^15^, ^17^]

Consequently, we exchanged lithium with the heavier homologs potassium (**1**) and sodium (**2**,**3**) to prevent additional LiHal‐co‐complexation during metathesis reactions.^[30]^ The resulting Dy^III^‐complexes exhibit SMM properties and were characterized by X‐ray diffraction and magnetic experiments. Herein, we present the synthesis and structural characterization of new alkali metal based triimidosulfites and their subsequent lanthanide(III) complexes. We investigated their magneto‐structural properties to evaluate the potential of sulfur‐nitrogen ligands for f‐metal based SMM design.

## Results and Discussion

### Precursor synthesis and XRD analysis

**Scheme 2:**
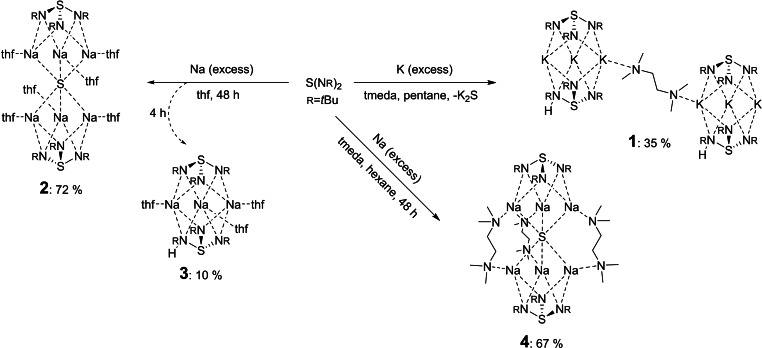
Synthesis of potassium triimidosulfite (**1**) and sodium triimidosulfites (**2**–**4**) from sulfurdiimide.

We herein present novel sodium and potassium based triimidosulfites. All complexes are accessible through the treatment of the pure metal with sulfurdiimide, which was prepared according to established methods.^[31]^ For [{S(*t*BuN)_2_(*t*BuNH)}_2_K_3_(tmeda)K_3_‐{(HN*t*Bu)(N*t*Bu)_2_S}_2_] (**1**), to an excess of potassium in a pentane/ N,N,N’,N’‐tetramethylethylenediamine (tmeda) (1 : 1) sulfurdiimide was added and stirred for 24 h. The remaining potassium and precipitated K_2_S were removed through filtration. Upon recrystallization from pentane twice, **1** was obtained as a dark‐red crystalline solid in a yield of 35 % (Scheme 2). We assume a one‐electron reduction pathway with radical intermediates as previously described.[^27^, ^28^, ^32^] Additionally, work directed towards the trimethylsilyl substituted sulfurdiimide S(NSiMe_3_)_2_ elucidates their radical properties, upon reaction with alkali metals, in lanthanide complexes.^[26]^

Similar reaction conditions were used for **2** and **3**. Sodium was combined with sulfurdiimide in thf, which resulted in a rapid color change to red. Notably, if the reaction time is shortened, an intermediate product [S(*t*BuN)_3_(thf)_3_Na_3_{(HN*t*Bu)(N*t*Bu)_2_S}] (**3**) is isolable in a low yield of 10 % as a red solid after 4 h. With allowing more reaction time, [S(*t*BuN)_3_(thf)_3_Na_3_SNa_3_(thf)_3_(N*t*Bu)_3_S] (**2**) is formed in high yield (72 %) and obtained as a colorless solid after 48 h, for details on the yield calculation procedure see Supporting Information, page 45 (Scheme 2). Both complexes were purified through recrystallization from a concentrated thf solution.

Applying the reaction conditions of **1** with the sodium metal, [(tmeda)_3_S{Na_3_(N*t*Bu)_3_S}_2_] (**4**) is received in a high yield of 67 % as a colorless solid. A rapid color change to red was observed for this reaction too. Detailed procedures and all analytic data for **1**–**4** are presented in the Supporting Information and the Experimental.

**Figure 1:**
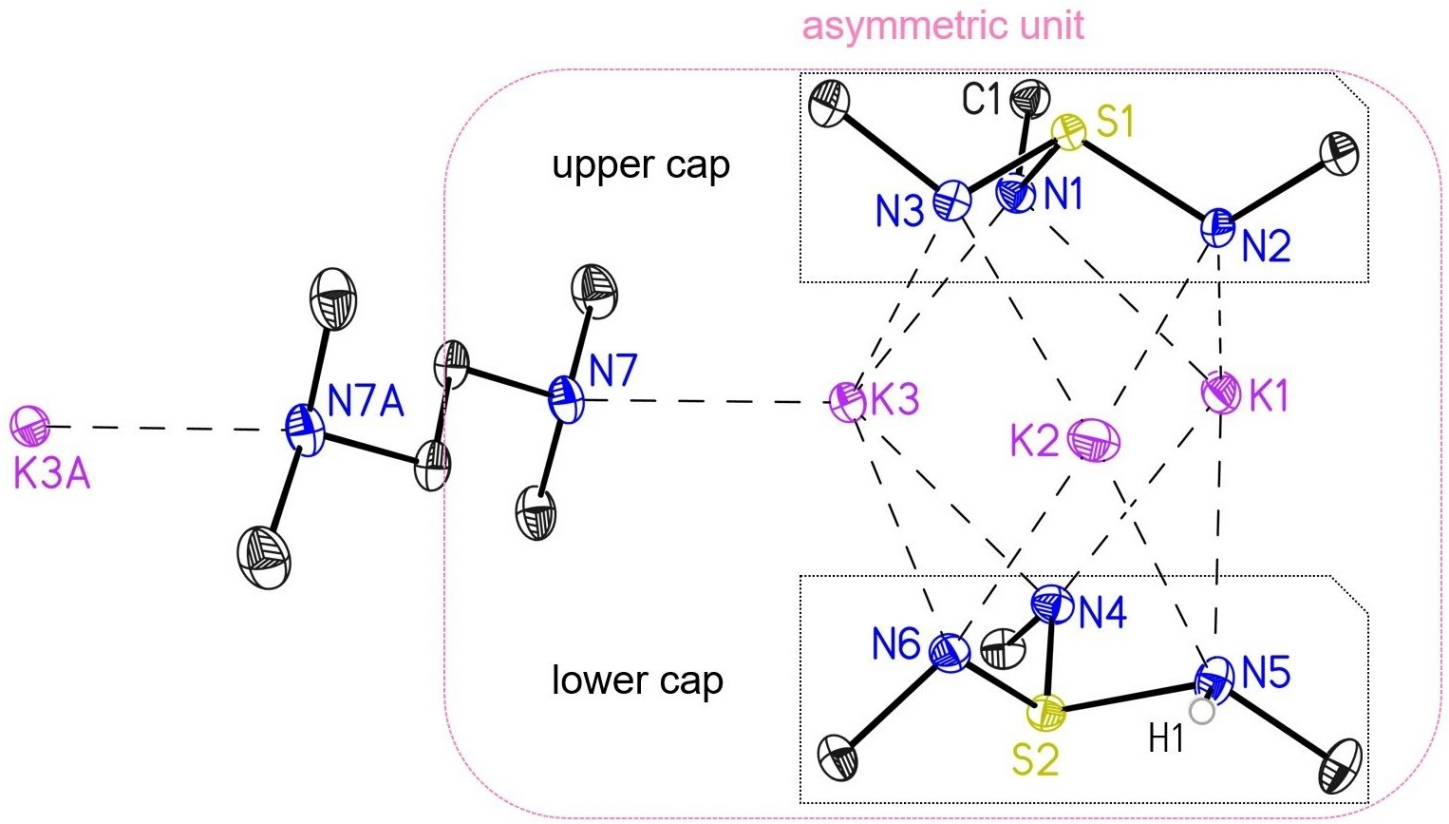
The solid‐state structure of **1** with anisotropic displacement parameters at the 50 % probability level. Note: The asymmetric unit consists of only one complex molecule and half of the tmeda (pink box). The *tert‐*butyl groups associated with each nitrogen atom of the lower and upper cap are truncated. All hydrogen atoms are omitted for clarity. The position of H1 at N5 could be taken from the Fourier map and refined freely.

**Figure 2:**
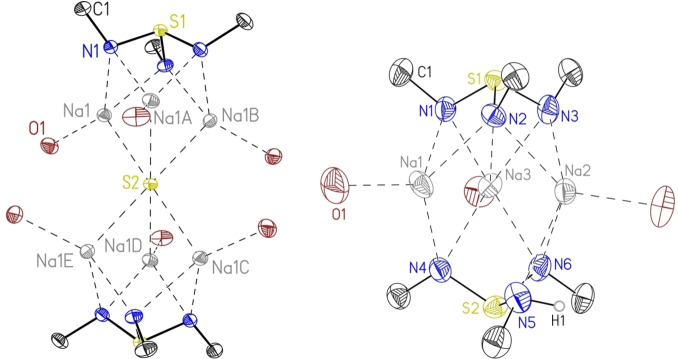
The solid‐state structure of **2** (left) and **3** (right) with the anisotropic displacement parameters at the 50 % probability level. The hydrogen atoms, except H1 at N(5) and the thf molecules bound to each sodium ion are omitted for clarity. The *tert‐*butyl groups associated with each nitrogen atom are truncated for clarity.

**Figure 3:**
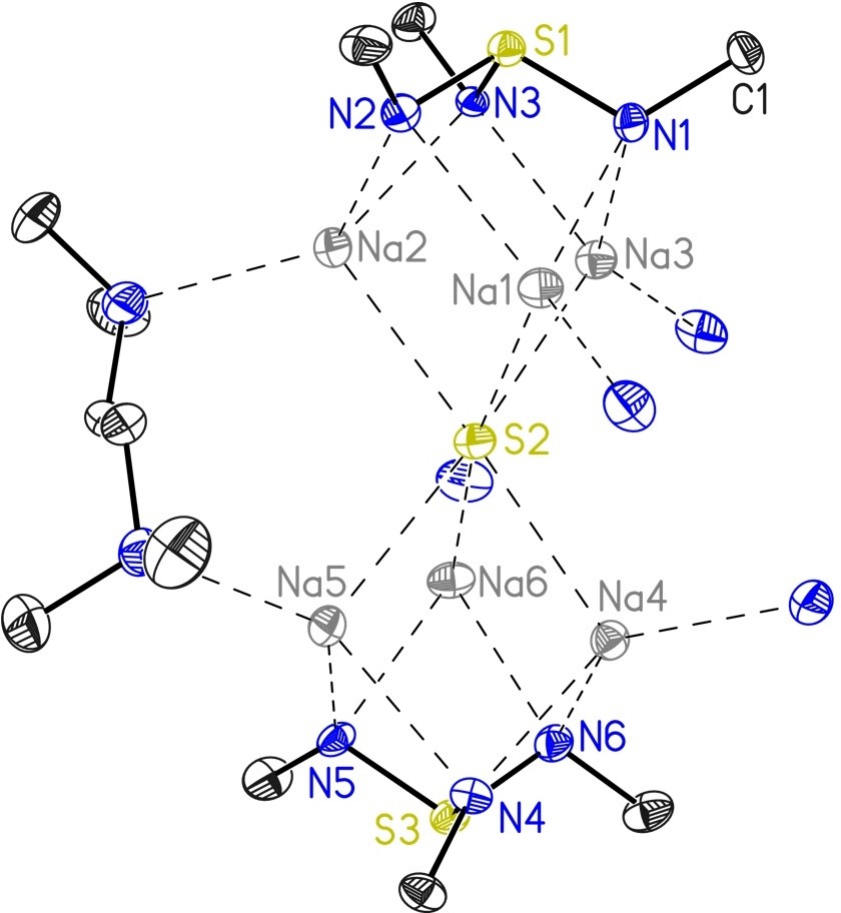
Solid‐state structure of **4** with the anisotropic displacement parameters at the 50 % probability level. The hydrogen atoms, the *tert‐*butyl groups associated with each nitrogen atom of the triimidosulfite and the tmeda moieties bridging from Na1 to Na4 and Na3 to Na6 are omitted for clarity.

[{S(*t*BuN)_2_(*t*BuNH)}_2_K_3_(tmeda)K_3_{(HN*t*Bu)(N*t*Bu)_2_S}_2_] (**1**) was obtained as dark‐red, block shaped crystals, and displays a monoclinic crystal system (*P*2_1_/c). The asymmetric unit of **1** consists of one complex molecule and half of the tmeda moiety, as depicted in the pink box in Figure 1. One upper and one lower triimidosulfite ligand encapsulate three potassium ions, while a tmeda moiety is bridging two complex units [L2 K3] through the K(3) atom, as depicted in Figure 1. The upper cap displays low variations in S−N bond lengths and angles, the S(1)−N distances ranging from 1.6422(14) to 1.6548(14) Å and the N−S(1)−N angles from 102.01(7)° to 105.51(7)° (Table 1), respectively. In contrast, the lower cap illustrates high deviations in the individual bond lengths and angles. This confirms the presence of the hydrogen atom at N(5), which results in an elongated S(2)−N(5) bond of 1.7590(15) Å and significant shorter S(2)−N(4) and S(2)−N(6) bonds with 1.5891(14) and 1.6133(14) Å, respectively. Moreover, the N(5)−S(2)−N(4) and N(5)−S(2)−N(6) angles are narrowed to 99.39(7)° and 95.78(7)°, while the N(6)−S(2)−N(4) angle is widened to 110.83(7)°. This phenomenon stems from the exchange of a sterically more demanding lone pair with a hydrogen atom. All further analyses are in good agreement with the XRD structure (see Experimental). [S(*t*BuN)_3_(thf)_3_Na_3_SNa_3_(thf)_3_(N*t*Bu)_3_S] (**2**)^[33]^ crystallizes in the cubic space group *Pa* $\bar{3}$ , with 1/6 of the molecule in the asymmetric unit, as portrayed in Figure 2, left. In this high symmetry, a sulfide S^2−^ centers a Na_6_ trigonal antiprism. **2** consists of two [S(N*t*Bu)_3_]^2−^ caps, associated with the three sodium ions of each side. Each sodium is additionally coordinated by one thf moiety. [(tmeda)_3_S{Na_3_(N*t*Bu)_3_S}_2_] (**4**) is similar to **2**, apart from the solvent coordination. Three tmeda molecules bridge two sodium ions from either side of the central sulfide dianion (Figure 3). For complex **4** we found a monoclinic space group (*P*2_1_/c), with two molecules in the asymmetric unit, as displayed in Figure 3. Noteworthy, the oxidation states of the sulfur atoms in the [S(N*t*Bu)_3_]^2−^ caps are +IV for all complexes, as shown in Scheme 1, while the bridging sulfide in **2** and **4** displays ‐II, and leads to a neutral complex charge. Therefore, the structure of **2** and **4** can be seen as composed from a Na_6_S^4+^ unit capped by two S(N*t*Bu)_3_]^2−^ ligands. Comparing this central Na_6_S^4+^ unit with the solid‐state structure of Na_2_S^[34]^ shows similar Na−S−Na angles in **2** (69.56(2), 110.44(2), 180.0 and 70.5, 109.5 and 180.0 in Na_2_S) although the coordination number increases from six in **2** and **4** to eight in Na_2_S. **4** follows this trend but displays deviations of the Na−S−Na angles. The smaller coordination number leads to shorter Na−S bond lengths [2.7691(8) in **2** and 2.7232(18)–2.7623(17) Å in **4**] compared to 2.83 Å in Na_2_S.

**Table 1:** Selected bond lengths [Å] and angles [°] for **1**–**4**.

Complex	**1**	**2**	**3**	**4**
M−N_range_^[a]^	2.6001(14)–	2.3300(17)	2.340(4)–	2.331(3)–
	3.2356(16)		2.705(4)	2.562(4)
M−M_range_	3.6449(7)–	3.1592(13)	3.1509(19)	3.151(2)–
	3.9473(7)		3.335(2)	3.228(2)
S(1)−N_range_	1.6442(14)–	1.6510(15)	1.586(4)–	1.645(3)–
	1.6548(14)		1.657(3)	1.655(3)
S(2/3)−N(4)	1.5891(14)	1.6510(15)	1.604(4)	1.658(3)
S(2/3)−N(5)	1.7590(15)	1.6510(15)	1.732(4)	1.648(3)
S(2/3)−N(6)	1.6133(14)	1.6510(15)	1.603(4)	1.653(3)
S(2/3)−M_range_	–	2.7691(8)	–	2.7232(18)–
				2.7623(17)
N−S(1)−	102.01(7)–	103.01(7)	99.09(18)–	102.04(16)–
N_range_	105.51(7)		103.13(18)	103.54(17)
N−S(2/3)−−	95.78(7)–	103.01(7)	96.1(2)–	102.43(16)–
N_range_	110.83(7)		103.13(18)	103.82(17)

[a] Disordered atoms are not considered.

The intermediate [S(*t*BuN)_3_(thf)_3_Na_3_{(HN*t*Bu)(N*t*Bu)_2_S}] (**3**) crystallizes in the orthorhombic space group *P*na2_1_ with one complex molecule in the asymmetric unit, shown in Figure 2, right. Three sodium ions are encapsulated by two [S(N*t*Bu)_3_]^2−^ caps, while each sodium atom coordinates an additional thf molecule. The M−N bond lengths for **2**–**4** compared to those in **1** assemble the expected decrease of the ionic radii from potassium to sodium. Therefore, shorter M−N bond length ranges 2.3300(17) Å for **2**, 2.340(4)–2.705(4) Å for **3**, and 2.331(3)–2.562(4) Å for **4** are reported compared to 2.6001(14)–3.2356(16) Å in **1**. The same applies for the shortened M⋅⋅⋅M distances of 3.1592(13) Å in **2**, 3.1509(19) to 3.335(2) Å in **3**, and 3.151(2)–3.228(2) Å in **4**, compared to 3.6449(7)–3.9473(7) Å found in **1** (see Table 1). Interestingly, apart from **2**, the alkali‐metal ions are not equally distributed from each other and exhibit a deviation of almost 0.2 Å, which may also arise from the protonation of one nitrogen atom or the tmeda coordination. For the cubic complex **2**, bond lengths and angles are identical for all symmetry generated positions, since only one sixth of the molecule is in the asymmetric unit. Consequently, we found 1.6510(15) Å for all S−N bond lengths and 103.01(7)° for the N−S−N angles, while the related complex **4** exhibits similar S−N bond lengths [1.645(3) – 1.663(3) Å] and N−S−N angles [102.04(16)°– 103.82(17) °]. Analogous to **1**, the lower cap of **3** displays high deviations of individual bond lengths and angles, due to the protonation of N(5) [S(2)−N(5) 1.732(4), S(2)−N(4) 1.604(4) and S(2)−N(6) 1.603(4) Å]. Therefore, also the corresponding N−S(2)−N angles exhibit a broad range from 96.1(2)° to 103.13(18)°. Usually, a hydrogen atom at this ligand class is hard to detect with standard NMR techniques due to its fast exchange rate. However, ^15^N{H}‐HMBC NMR data confirmed the protonation of one nitrogen atom also in solution (for details see experimental part).

### Syntheses and XRD analyses of 5–7

**Scheme 3:**
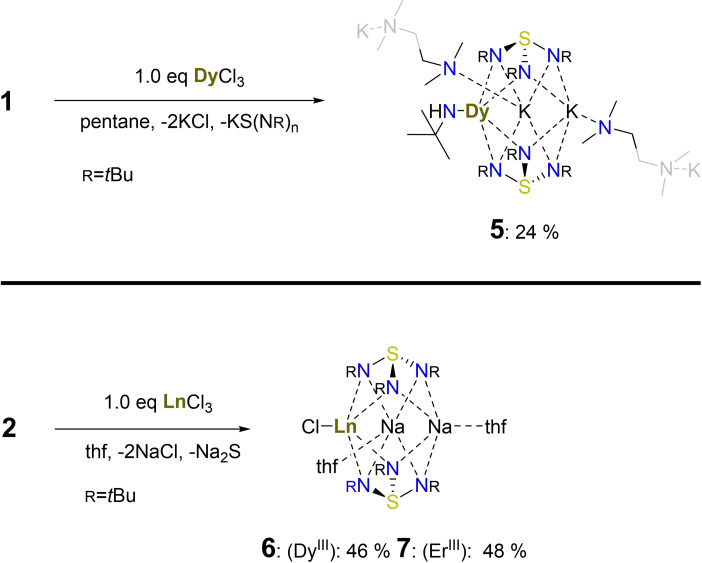
(top) Synthesis of complex **5** from potassium triimidosulfite (**1**) in pentane. (bottom) Preparation of compounds **6** and **7**: Treatment of **2** with Dy^III^‐ and Er^III^‐chloride in thf yields the desired complexes.

**5** [(*t*BuNH)Dy{K(0.5tmeda)}_2_{(N*t*Bu)_3_S}_2_]_*n*_ is accessible through a metathesis reaction of **1** with Dy^III^ chloride in pentane at ambient temperatures, depicted in Scheme 3. We were able to record ^1^H NMR spectra for this strong paramagnetic complex with all expected signals, for further details, the reader is referred to the experimental part. [ClLn{Na(thf)}_2_{(N*t*Bu)_3_S}_2_] with Ln=Dy (**6**), Er (**7**) were obtained as stated in Scheme 3, bottom, and in the experimental.

**Figure 4:**
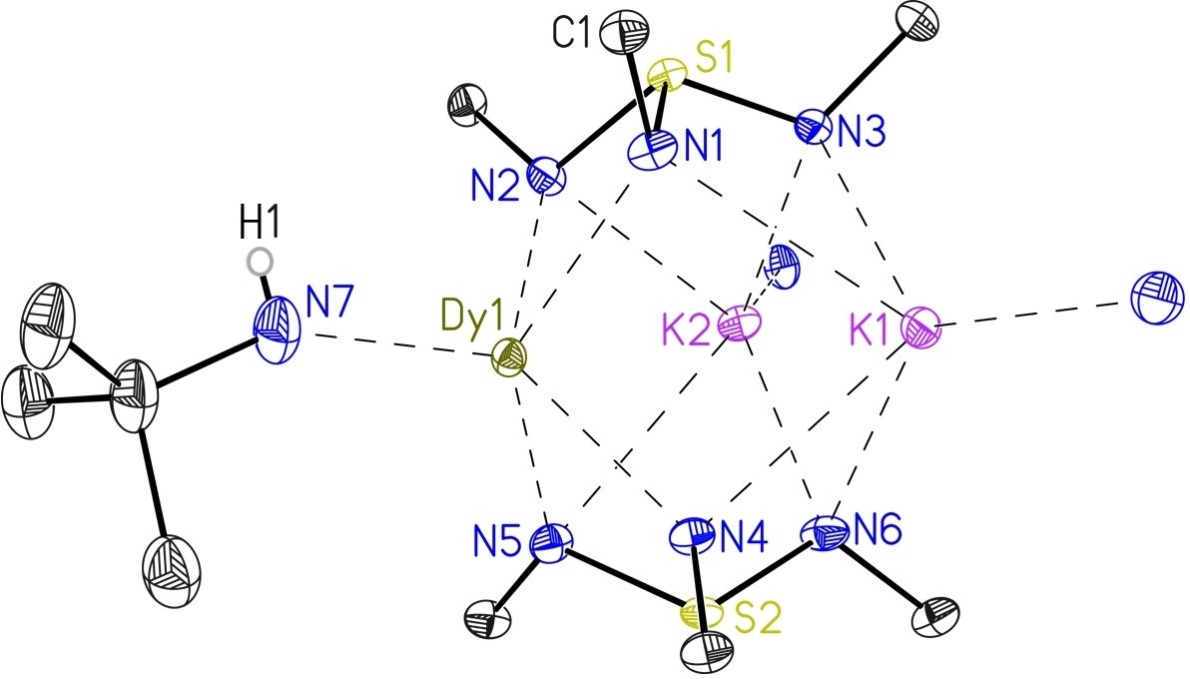
Solid‐state structure of **5** with the anisotropic displacement parameters at the 50 % probability level. The hydrogen atoms, except H1 at N(7), are omitted and the triimidosulfite associated *tert‐*butyl groups are truncated for clarity.

**5** crystallizes in the triclinic space group *P* $\bar{1}$ .The mixed‐metal complex comprises two capping ligands encapsulating two potassium ions and one dysprosium ion.^[35]^ The Dy^III^ ion is additionally coordinated to a negatively charged *t*BuNH amide moiety. This arises from ligand scrambling since this precursor (**1**) is not as selective as the sodium pendant (**2**). However, despite the lowered yield from the ligand scrambling, we successfully isolated **5** as crystalline material. **5** forms a chain‐like structure through the potassium ions bridged through tmeda donor bases, as illustrated in Figure 4 and the Supporting Information. It, therefore, confirms the adequate substitution of one K‐ion with a Dy^III^ ion.

**Figure 5:**
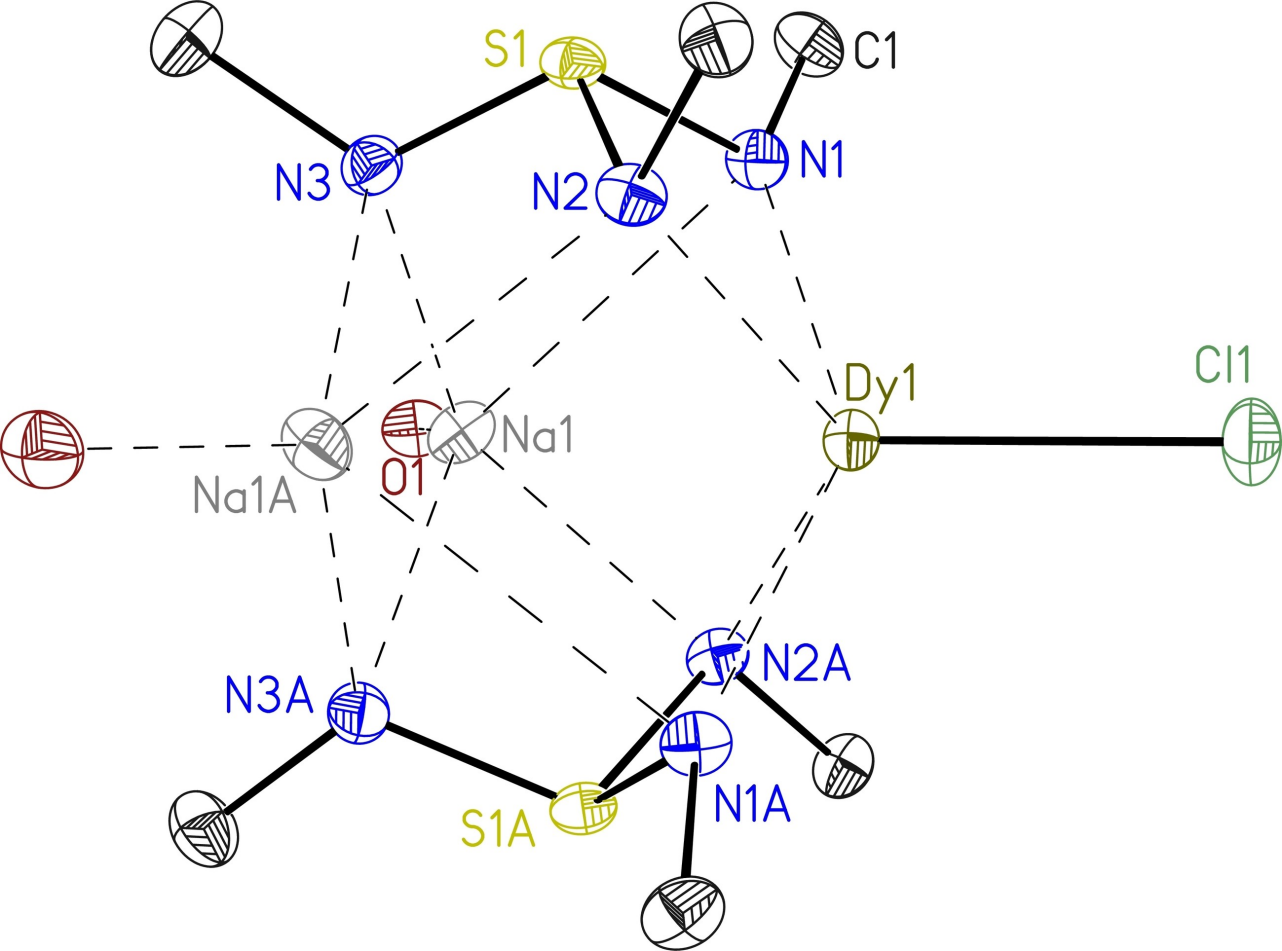
The solid‐state structure of **6**. The Er^III^ complex **7** is isostructural. The anisotropic displacement parameters are displayed at the 50 % probability level. The hydrogen atoms and the thf molecules bound to each sodium‐ion are omitted for clarity. The *tert‐*butyl groups associated with each nitrogen atom are truncated for clarity.

The isomorphous complexes **6** and **7** crystallize in the monoclinic space group *C*2/c as colorless blocks and display only half a molecule in the asymmetric unit (Figure 5). Their structure motive is similar to **5**, apart from the remaining chloride anion coordinated to the dysprosium instead of the amide. The slightly longer Dy−N bond lengths for **5** [2.3239(17)–2.3865(17) Å, see Table 2] are in good agreement with the expected ionic radii trend from sodium to potassium. The further Ln−N bond lengths decrease from **6** to **7** is due to the lanthanide contraction. Similarly, the decrease of Ln(1)⋅⋅⋅Na(1) distances from **6** to **7** can be attributed to the ionic radii decrease. Two nitrogen atoms of the triimidosulfite unit coordinate the lanthanide(III) ion with little variation for the individual S−N bond lengths, which is characteristic of the high adaptability of SN ligands in metal complexes.^[16]^ In **5** they range from 1.6709(17) to 1.6760(17) Å, while in **6** from 1.6667(13) to 1.6875(13) Å, and in **7** from 1.6632(15) to 1.6892(16) Å. However, since N(3) and N(6) do not participate in Ln‐metal bonding but alkali‐metal bonding, their corresponding S−N bond lengths are significantly shorter, 1.6097(16) and 1.6101(17) Å in **5**, 1.6141(13) Å in **6** and 1.6102(16) Å in **7**.

**Table 2:** Selected bond lengths [Å] and angles [°] for **5–7**.

Complex	**5**	**6**	**7**
Ln(1)−N_range_	2.3239(17)–2.3865(17)	2.2985(13)–2.3702(13)	2.2868(16)–2.3403(16)
Ln(1)−M(1)	3.9188(10)	3.5151(7)	3.4965(9)
S(1)−N(1)	1.6760(17)	1.6667(13)	1.6632(15)
S(1)−N(2)	1.6724(17)	1.6875(13)	1.6892(16)
S(1)−N(3)	1.6097(16)	1.6141(13)	1.6102(16)
S(2)−N(4)	1.6719 (16)	–	–
S(2)−N(5)	1.6709(17)	–	–
S(2)−N(6)	1.6101(17)	–	–
Dy(1)−N(7)	2.199(2)	–	–
Ln(1)−Cl(1)	–	2.5564(6)	2.5203(8)
N(1)−S(1)−N(2)	96.30(8)	96.53(6)	96.32(8)
N(5)−S(2)−N(4)	95.30(8)	–	–
N(3)−S(1)−N(1)	107.85(9)	105.21(7)	105.32(8)
N(3)−S(1)−N(2)	104.80(9)	106.44(7)	106.47(8)
N(6)−S(2)−N(4)	106.75(9)	–	–
N(6)−S(2)−N(5)	106.97(9)	–	–

While in **5** the Dy^III^ ion is coordinated to a *tert*‐butylamide, showing the shortest Dy−N bond length of 2.199(2) Å, one chloride ligand remains attached to the lanthanides in **6** and **7**, with the corresponding Ln(1)−Cl(1) bond lengths in the range with previously reported structures.[^10^, ^36^] Furthermore, the N(1)−S(1)−N(2) angles are the smallest observed angles with 96.30(8) for **5**, 96.53(6) for **6**, and 96.32(8)° for **7**, respectively. This agrees with the tightening of the N(1)⋅⋅⋅N(2) distance, caused by the lanthanide coordination.

### Magnetic properties

**Figure 6:**
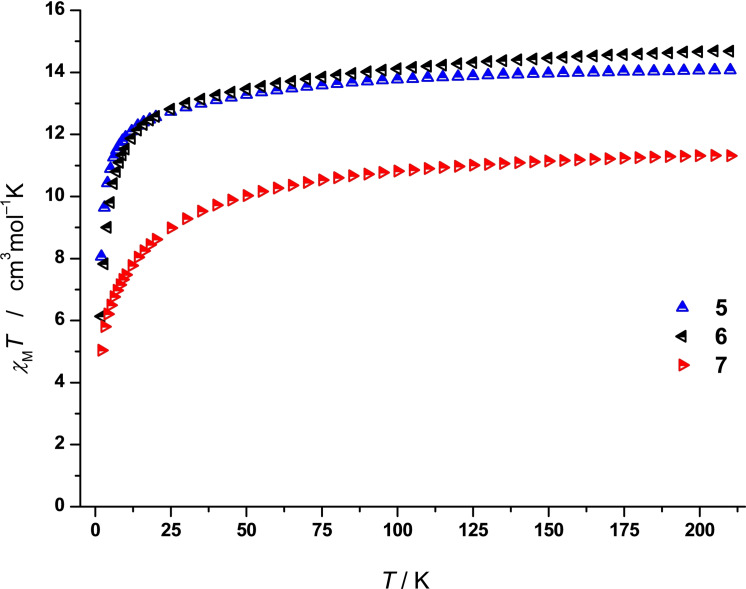
*χ*_M_ *T* vs. *T* for **5** (blue), **6** (black), and **7** (red) under an applied field of *H*_dc_ = 5000 Oe.

We determined the temperature‐dependency of the magnetic susceptibility as the product of the temperature (*χ*_M_ *T*), applying a direct current (dc) field of 5000 Oe. Measured values of *χ*_M_ *T* (cm^3^mol^−1^K) exhibit 14.07 for **5**, 14.68 for **6**, and 11.31 for **7** at 210 K, where the calculated ones for the free ions are 14.17 for both **5** and **6**, while 11.48 for **7**.^[37]^ The measured *χ*_M_ *T* values for **5** and **7** are slightly lower than the calculated ones, while **5** is slightly above the calculated value, but still within the range of related compounds. With reducing the temperature to 25 K, an almost linear decay of *χ*_M_ *T* occurs, which is in alignment with the successive depopulation of the Stark sublevels.[^19^, ^38^] Below 25 K the decrease is more rapid and displays values of 8.06 (**4**), 6.13 (**5**), and 5.04 (**6**) cm^3^mol^−1^K at 2 K, respectively (Figure 6). This phenomenon is caused by spin‐orbit coupling effects and all presented *χ*_M_ *T* values are in the range of previously reported complexes of this class.[^2^, ^20^, ^37^, ^39^]

We subsequently conducted magnetic susceptibility measurements under a dynamic alternating current (ac) field of 3 Oe for compounds **5–7**. For the Er^III^ complex **7**, there was no out‐of‐phase signal (*χ”*_M_) typical for SMM behavior under zero dc field, probably due to strong QTM effects. To prevent such fast relaxation processes, an external dc field was applied, and the highest *χ”*_M_ value was found for an optimal field of 1000 Oe. However, **7** shows only a maximum slightly above 2 K and a rapid decay of *χ”*_M_ , suggesting that QTM still occurs (see Supporting Information, Figure S38) and that this type of ligand design may not be suitable for prolate‐shaped lanthanide ions. Since dysprosium containing molecules are more prone to display SMM behavior, even under zero dc field, we were expecting more interesting properties for complexes **5** and **6**. Indeed, both Dy^III^ complexes display out‐of‐phase signals of the dynamic susceptibility (*χ”*_M_) under zero dc field. Maxima in the *χ”*_M_ versus frequency plot were detected up to 7.0 K for **5**, while one relaxation process was applied to fit the data with the CC‐fit program to generate the Cole‐Cole plot fit.^[40]^ The corresponding Arrhenius plot was constructed from the output relaxation times *τ*_1_. Considering Raman, Orbach, QTM and direct relaxation processes we employed Equation (1) to determine the energy barrier:

(1) $$\tau^{-1}=\;{\tau_{0}}^{-1}{\cdot e}^{{-U}_{eff}/k_{B}T}+CT^{n}+\;\tau_{QTM}^{-1}$$

**Figure 7:**
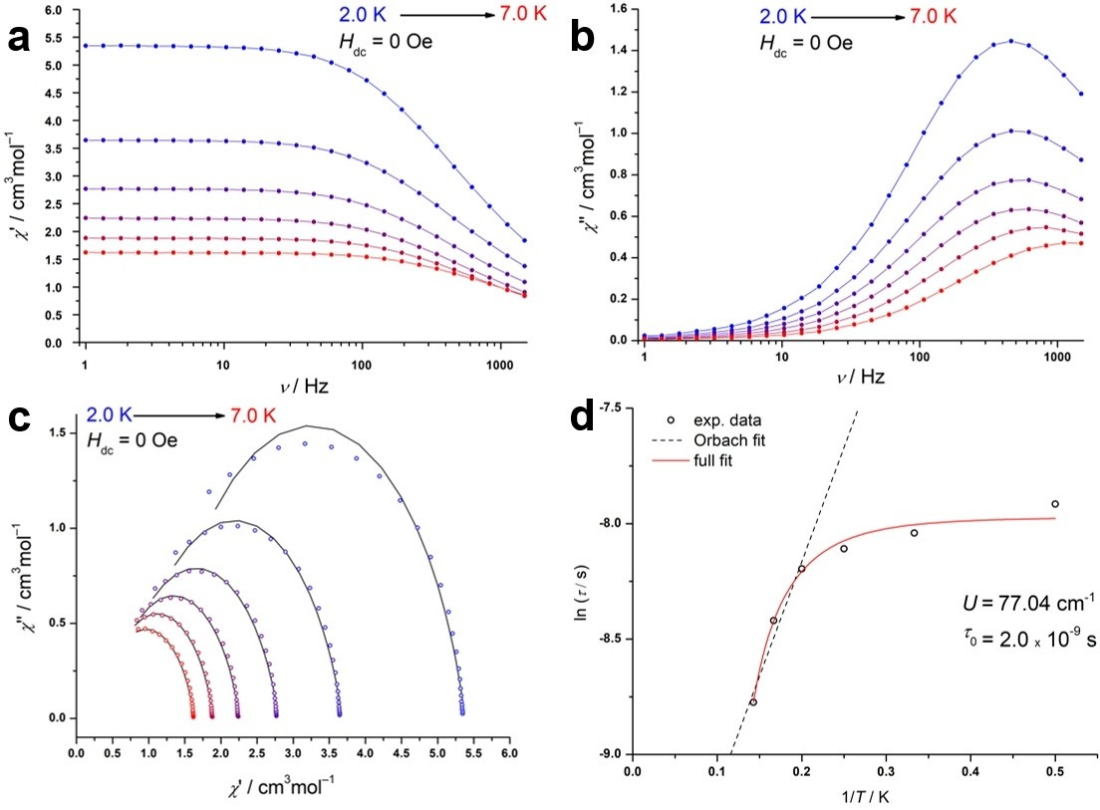
Magnetic data for **5** under zero field conditions. Dynamic in‐phase a) and out‐of‐phase b) susceptibility versus variable frequencies from 2 to 7 K. c) Cole‐Cole plot with corresponding CC‐fit data; d) Arrhenius plot with red curve exemplifying the full fit of all relaxation processes and the dash line demonstrates a sole Orbach regression.

From the full fit we obtained an energy barrier of *U*=77.04 cm^−1^ for **5**, illustrated by the red full‐fit curve in Figure 7, while relevant magnetic data is summarized in Table 3.

**Table 3:** Selected magnetic data for **5** and **6** under zero field conditions.

Complex	**5**	**6^[c]^**	**6^[d]^**
*U* ^[a]^ (cm^−1^)	77.04	512.85	316.34
*U*_eff_^[b]^ (cm^−1^)	14.9	403.3	297.2
*τ*_0_ (s)	2.01×10^−9^	6.66×10^−10^	8.25×10^−10^
*n*	4.59	5.17	4.41

[a] *U* refers to the energy barrier for the full fit with Equation (1), [b] *U*_eff_ refers to a linear approximation of a sole Orbach relaxation and is reported in the Supporting Information together with all best fitting parameters and their errors, [c] corresponds to the data for the first relaxation process, [d] corresponds to the data for the second relaxation process.

Subsequently, we tried to suppress QTM, which was observable at low temperatures (Figure 7d), by applying a dc field. The optimal field was determined to be 100 Oe, at which the dynamic out‐of‐phase susceptibility reached its highest maximum. Interestingly, the applied field initiates a second slow relaxation process, which is observable with a second local maximum at around 10 Hz at 3 K in the *χ”*_M_ versus *ν* plot, as observable in the corresponding Figure S28 in the Supporting Information.

**Figure 8:**
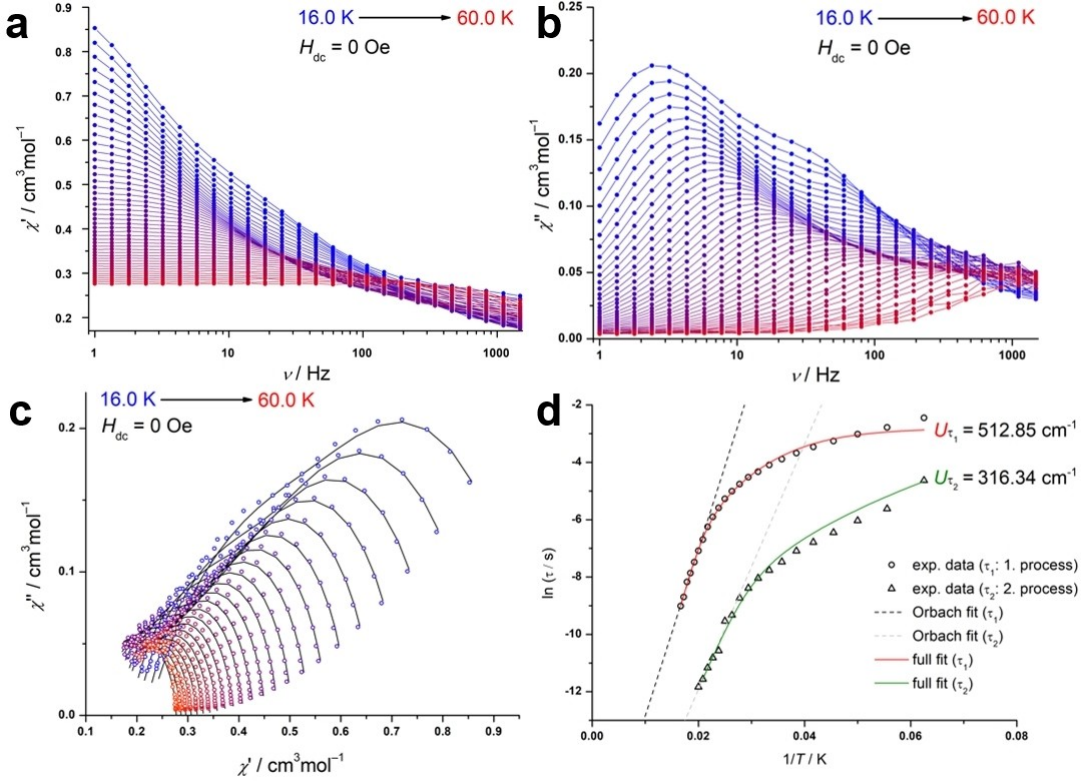
Magnetic data for **6** under zero field conditions. Dynamic in‐phase a) and out‐of‐phase b) susceptibility versus variable frequencies from 16 to 60 K. c) Cole‐Cole plot with corresponding CC‐fit data; d) Arrhenius plot with red curve exemplifying the full fit of all relaxation processes and the dash line demonstrates a sole Orbach regression.

**Figure 9:**
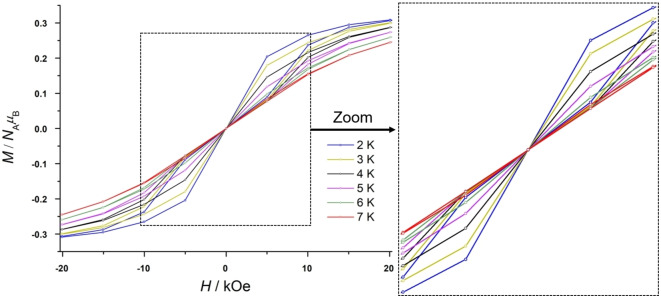
Field dependency of the magnetization for **6** from 2 to 7 K at a sweep rate of 185 Oe/s.

**Figure 10:**
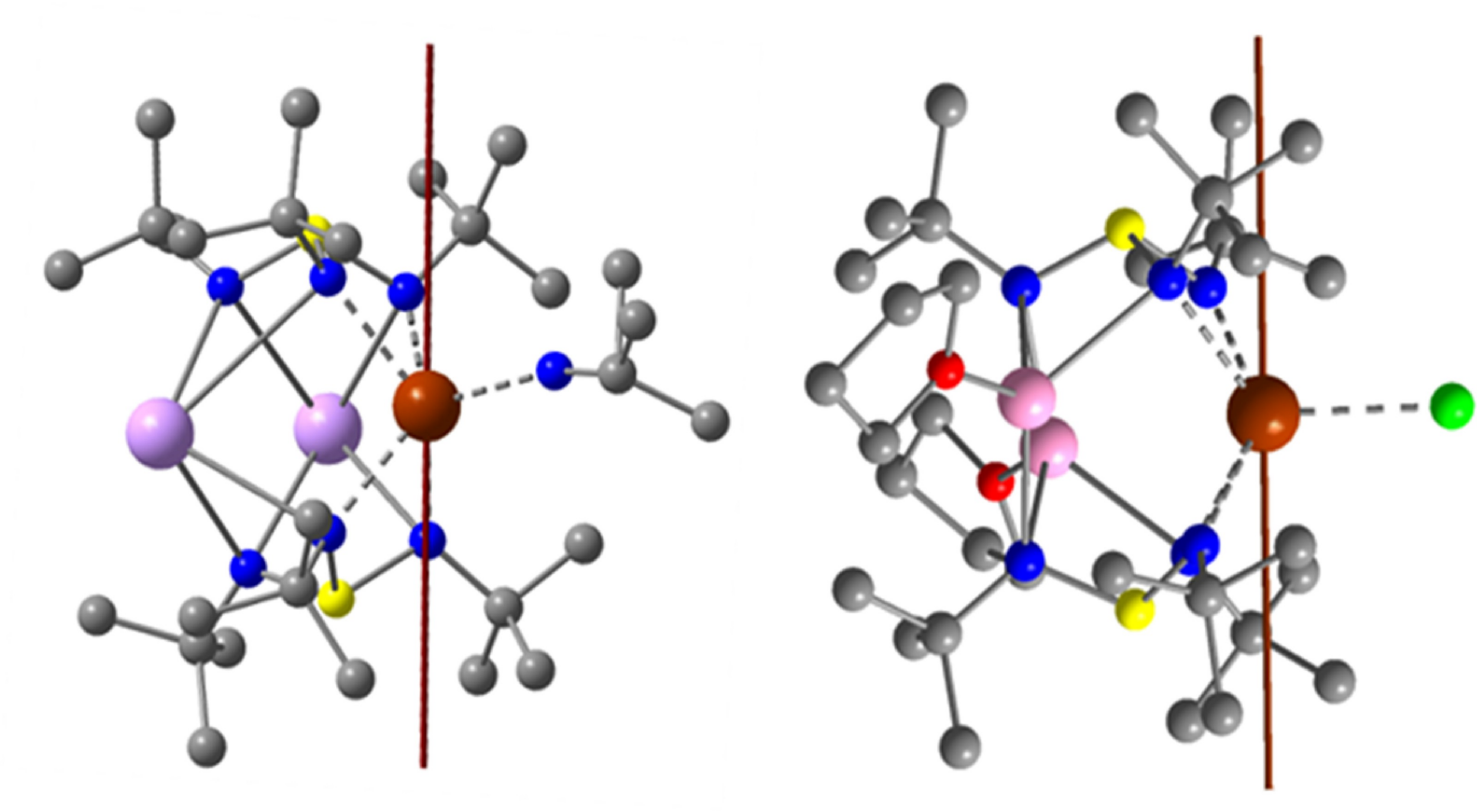
Orientation of the main magnetic axis (brown) in **5** (left) and **6** (right). Color code: C (grey), O (red), N (blue), S (yellow), K (purple), Na (pink), Dy (brown), and Cl (green).

Complex **6** exhibits SMM behavior as aforementioned and as illustrated in Figure 8(b). Below 16 K, no maxima for the dynamic out‐of‐phase susceptibility (*χ”*_M_ vs frequency plot, Figures S32, S33 in the Supporting Information) are observed, and QTM is the dominant process,. Two local maxima in the out‐of‐phase signal are detected at a frequency of 3 Hz and 34 Hz at 16 K. Increasing the temperature up to 60 K results in a characteristic shift of the maxima to the higher frequencies, indicating a true temperature‐depending relaxation process and a typical SMM‐like behavior. The Cole‐Cole plot was fitted with applying two relaxation processes within the CC‐fit program. The two sets of output relaxation times were used to draw and fit both relaxation processes in the Arrhenius plot (Figure 8d). A sole Orbach approximation of the Arrhenius plot exhibits a significant higher effective energy barrier for *τ*_1_ (403.3 cm^−1^) and *τ*_2_ (297.2 cm^−1^) than observed for **5** (14.9 cm^−1^), as demonstrated by the dashed lines in Figure 8(d). Acknowledging the other relaxation processes (by using Eq1), we found even higher energy barriers of *U* τ_1_=512.9 cm^−1^ for τ_1_ and *U* τ_*2*_=316.3 cm^−1^ shown as the red fitting curve in Figure 8(d). The corresponding best fit parameters are illustrated in Table 3 and the Supporting Information in Tables S10 and S11. Further enhancement of the magnetic properties could not be achieved by applying an external field. Under these conditions no maxima for the frequency dependency of *χ”*_M_ did occur (see Supporting Information, Figure S31) Magnetization studies showed that compound **6** can retain its magnetization to a certain extent if the applied magnetic field is not entirely removed. Indeed, the presence of QTM at low temperatures prevents the observation of magnetic remanence at 0 dc field and results in a butterfly‐shaped hysteresis,^[41]^ as shown in Figure 9. The wings of the hysteresis are open at emperatures up to 7 K with a sweep rate of 185 Oe/s. A similar hysteresis was reported for Cp^ttt^_2_DyCl,^[10]^ which makes **6** an equal contender. The observation of this magnetic hysteresis suggests that the present ligand design is quite suitable for dysprosium based SMMs, however, some improvement could potentially result in an open hysteresis and a higher blocking temperature. In order to determine the next areas of investigations towards this aim, we calculated the main magnetic axis in complexes **5** and **6** based on the electrostatic model in the program Magellan as exemplified in Figure 10.^[42]^ The main magnetic axes are oriented similarly in both complexes, showing a small deviation of the Dy−N bonds, which suggests a relatively high local axiality at the dysprosium center. If translated to the Er analogous complex, this observation might further explain why no SMM behavior is detected for **7** and confirms that the present ligands generate a ligand field beneficial to oblate‐shaped lanthanides, such as the Dy^3+^ ion. The main magnetic axes are also located perpendicularly to the nitrogen atom of the *tert*‐butylamide in **5** and to the halide atom in **6**, respectively, resulting in substantial transverse anisotropy.

Thus, the next steps should focus on the removal of these additional donors, which are likely hampering the achievement of optimal axial anisotropy in the present complexes.

## Conclusion

In developing a new route for promising triimidosulfite precursors, we have introduced the potassium based [{S(*t*BuN)_2_(*t*BuNH)}_2_K_3_(tmeda)K_3_{(HN*t*Bu)(N*t*Bu)_2_S}_2_] (**1**) and the sodium [S(*t*BuN)_3_(thf)_3_Na_3_SNa_3_(thf)_3_(N*t*Bu)_3_S] (**2**) complexes. Both effectively suppress LiHal co‐complexation, which turned out to be the main hindrance for SN ligands to enhance the SMM properties. The subsequent Dy complex [(*t*BuNH)Dy{K(0.5tmeda)}_2_{(N*t*Bu)_3_S}_2_]_n_ (**5**) exhibits SMM behavior under zero field conditions. We assume the additional *t*BuNH coordination to be the crucial factor in lowering its energy barrier *U*_eff_ to only 70.0 cm^−1^. However, [ClDy{Na(thf)}_2_{(N*t*Bu)_3_S}_2_] (**6**) demonstrates the high potential of SN ligands in SMM chemistry. Indeed, for complex **6**, we found an energy barrier of 515.9 cm^−1^ (*τ*_1_) and 316.2 cm^−1^ (*τ*_2_) for the fullfit of all relaxation processes and a frequency dependency of the dynamic out‐of‐phase susceptibility up to 60 K. Excitingly, **6** also shows a butterfly shaped magnetic hysteresis up to 7 K. Looking forward, the abstraction of the remaining chloride from **6** could lead to [(thf)_2_Na_2_Dy{S(N*t*Bu)_3_}_2_]^+^ and substantially increase the axial anisotropy, consequently leading to better overall magnetic properties.

## Experimental Section

### General procedures

All reactions were carried out in flame‐dried glassware under dry argon conditions, applying Schlenk techniques or done in an argon dry box.^[43]^ All solvents were distilled from sodium or potassium and stored over 3 Å molecular sieve prior to use. Filtrations were performed with Whatman^TM^ glass microfiber filters GF/B standard. The lanthanide salts were purchased from Sigma‐Aldrich at 99.999 % purity level and used as received. Starting materials were purchased commercially and used without further purification. ^1^H‐, ^13^C‐, and ^15^N NMR experiments were performed on Bruker Avance 500 MHz, Bruker Avance 400 MHz, and Bruker Avance 300 MHz spectrometers. NMR data were referenced to the deuterated solvent (thf‐*d*_8_), which was dried over activated molecular sieves.^[44]^ Elemental analyses (C, H, N, S) were carried out on a Vario EL3 at the Mikroanalytisches Labor, Institut für Anorganische Chemie, Universität Göttingen. Some of the structures contain lattice solvent, as shown by X‐ray diffraction experiments, since the crystals were grown in the mother liquor. Even overnight drying under reduced pressure of these samples could not remove the entire amount of lattice solvent. The remaining solvent contributed to slightly increased values in the elemental analyzes for C and H. All magnetic data were recorded with a Quantum‐Design MPMS‐XL‐5 SQUID magnetometer equipped with a 5 T magnet. The crystalline material was crushed into crystalline powder, transferred into a gel capsule, and coated with a few drops of inert oil (perfluoropolyether Fomblin YL VAC 25/6; this oil is solid under 210 K). The oil avoids magnetic torquing and evaporation of coordinated or lattice solvent molecules. A smaller capsule was placed on the oil to seal the sample. Each capsule was positioned in a nonmagnetic sample holder, which was then inserted in the SQUID magnetometer for measurement. The obtained experimental data were worked up in the OriginPro and CCFit programs.^[40]^ The calculations of the main magnetic axis were performed with the Magellan program.^[42]^

All crystals were selected from a Schlenk line under cooling, using a X‐Temp2 device.^[45]^ Crystallographic data were collected with an Incoatec Mo−IμS microfocus source, a Bruker TXS rotating‐Mo‐anode, or a Bruker Smart Apex with Incoatec IμS−Ag‐Microsource on an Apex II CCD detector.^[46]^ The diffractometers contain an Oxford Cryosystems crystal cooling device and are equipped with Helios or Incoatec Quazar mirror optics. The data were integrated with SAINT.^[47]^ A multi‐scan absorption correction with SADABS was applied.^[48]^ The structures were solved by direct methods SHELXT^[49]^ and refined on *F* ^2^ using the full‐matrix least‐squares methods of SHELXL^[50]^ in the graphical user interface ShelXle.^[51]^ More details on the crystallographic data and the refinement can be found in the Supporting Information. Deposition Numbers 2128424 (for **1**), 2128425 (for **2**), 2128426 (for **3**), 2128427 (for **4**), 2128428 (for **5**) 2128429 (for **6**), 2128430 (for **7**) contain the supplementary crystallographic data for this paper. These data are provided free of charge by the joint Cambridge Crystallographic Data Centre and Fachinformationszentrum Karlsruhe Access Structures service.

Crystal data for **1** at 100(2) K: C_54_H_126_K_6_ N_6_S_4_ *M*_r_=1334.52 g mol^−1^, 0.377×0.281×0.190 mm, monoclinic, *P*2_1_/*c*, *a*=15.380(2) Å, *b*=13.885(2) Å, *c*=17.912(3) Å, *β*=95.74(2)°, *V*=3806.0(10) Å^3^, Z=2, *μ*(Ag *K*_α_)=0.258 mm^−1^, *Θ*_max_=20.252°, 141047 reflections measured, 7484 independent (*R*_*i*nt_=0.0722), *R*_1_=0.0296 [*I*>2*σ*(*I*)], w*R*_2_=0.0722 (all data), res. density peaks: 0.306 to −0.353 e Å^−3^.

Crystal data for **2** at 100(2) K: C_52_H_110_N_6_Na_6_O_7_S_3_ *M*_r_=1165.57 g mol^−1^, 0.407×0.400×0.305 mm, cubic, *Pa* $\bar{3}$ , *a*=18.917(2) Å, *V*=6770(2) Å^3^, Z=4, *μ*(Ag *K*_α_)=0.107 mm^−1^, *Θ*_max_=19.994°, 32527 reflections measured, 2148 independent (*R*_int_=0.0722), *R*_1_=0.0370 [*I*>2*σ*(*I*)], w*R*_2_=0.0942 (all data), res. density peaks: 0.250 to −0.225 e Å^−3^.

Crystal data for **3** at 100(2) K: C_36_H_79_N_6_Na_3_O_3_S_2_ *M*_r_=777.14 g mol^−1^, 0.355×0.283×0.272 mm, orthorhombic, *Pna*2_1_, *a*=15.072(2) Å, *b*=17.049(3) Å, *c*=17.726(3) Å, *V*=4554.9(13) Å^3^, Z=4, *μ*(Mo *K*_α_)=0.184 mm^−1^, *Θ*_max_=26.715°, 73524 reflections measured, 8681 independent (*R*_*i*nt_=0.0414), *R*_1_=0.0454 [*I*>2*σ*(*I*)], w*R*_2_=0.1193 (all data), res. density peaks: 0.397 to −0.212 e Å^−3^.

Crystal data for **4** at 100(2) K: C_42_H_102_N_12_N_6_S_3_ *M*_r_=1009.47 g mol^−1^, 0.254×0.212×0.145 mm, monoclinic, *P*2_1_/*c*, *a*=19.880(3) Å, *b*=17.651(2) Å, *c*=36.973(3) Å, *β*=90.00(2)°, *V*=12974(3) Å^3^, Z=8, *μ*(Mo *K*_α_)=1.034 mm^−1^, *Θ*_max_=25.693°, 126730 reflections measured, 24641 independent (*R*_*i*nt_=0.0655), *R*_1_=0.0456 [*I*>2*σ*(*I*)], w*R*_2_=0.1078 (all data), res. density peaks: 0.328 to −0.304 e Å^−3^.

Crystal data for **5** at 100(2) K: C_78_H_184_Dy_2_K_4_N_18_S_4_ *M*_r_ =1984.06 g mol^−1^, 0.241×0.231×0.184 mm, triclinic, *P* $\bar{1}$ , *a*=11.149(2) Å, *b*=12.947(2) Å, *c*=18.319(3) Å, *α*=82.43(2)°, *β*=79.11(2)°, *γ*=82.64(3)° *V*=2559.5(8) Å^3^, Z=1, *μ*(Ag *K*_α_)=0.930 mm^−1^, *Θ*_max_=21.787°, 150125 reflections measured, 12369 independent (*R*_*i*nt_=0.0406), *R*_1_=0.0227 [*I*>2*σ*(*I*)], w*R*_2_=0.0524 (all data), res. density peaks: 2.483 to −1.160 e Å^−3^.

Crystal data for **6** at 100(2) K: C_32_H_70_ClDyN_6_N_2_S_2_ *M*_r_=878.99 g mol^−1^, 0.355×0.138×0.134 mm, monoclinic, *C*2/*c*, *a*=11.858(2) Å, *b*=18.318(2) Å, *c*=19.385(3) Å, *β*=90.26(2)°, *V*=4210.7(11) Å^3^, Z=4, *μ*(Mo *K*_α_)=1.992 mm^−1^, *Θ*_max_=26.796°, 60115 reflections measured, 4490 independent (*R*_*i*nt_=0.0193), *R*_1_=0.0152 [*I*>2*σ*(*I*)], w*R*_2_=0.0405 (all data), res. density peaks: 0.789 to −0.272 e Å^−3^.

Crystal data for **7** at 100(2) K: C_32_H_70_ClErN_6_N_2_S_2_ *M*_r_=883.75 g mol^−1^, 0.454×0.430×0.360 mm, monoclinic, *C*2/*c*, *a*=11.822(2) Å, *b*=18.247(3) Å, *c*=19.388(3) Å, *β*=90.41(2)°, *V*=4182.2(11) Å^3^, Z=4, *μ*(Ag *K*_α_)=1.194 mm^−1^, *Θ*_max_=21.932°, 65058 reflections measured, 5148 independent (*R*_int_=0.0370), *R*_1_=0.0210 [*I*>2*σ*(*I*)], w*R*_2_=0.0546 (all data), res. density peaks: 1.523 to −1.219 e Å^−3^.

**[{S(*t*BuN)_2_(*t*BuNH)}_2_K_3_(tmeda)K_3_{(HN*t*Bu)(N*t*Bu)_2_S}_2_] (1)**: To potassium (0.94 g, 24.06 mmol, 2.33 eq.) in a pentane/tmeda mixture (10 mL, 1 : 1) S(*t*BuN)_2_ (2 mL, 10.32 mmol, 1 eq.) was slowly added at room temperature and stirred for 24 h. Instantaneously, a color change to dark red is observable and after 24 h a dark red solution is obtained. Filtration via a glass fritted filter (P4) over celite and crystalizing twice from pentane (3 mL) yielded the red product. Crystals suitable for X‐ray diffraction analysis were gained from a saturated solution of **1** in pentane at −30 °C. Overall yield was 0.81 g (35 %). ^1^H NMR (500 MHz, benzene‐*d*_6_, ppm) *δ*: 2.38 (m, 4H, CH_2_), 2.13 (s, 12H, N(C*H*_3_)_2_), 1.45 (s, 27H, NC(C*H*_3_)_3_)), 1.16 (s, 18H, NC(CH_3_)_3_), 1.08 (s, 9H, NHC(C*H*_3_)_3_).^13^C NMR (125 MHz, benzene‐*d*_6_, ppm) *δ*: 57.56 (2 C, *C*H_2_), 52.77 (2 C, N*C*(*C*H_3_)_3_), 52.33 (3 C, N*C*(*C*H_3_)_3_), 45.26 (4 C, N(*C*H_3_)_2_), 51.20 (1 C, NH*C*(*C*H_3_)_3_), 34.74 (9 C, NC(*C*H_3_)_3_), 33.79 (6 C, NC(*C*H_3_)_3_), 30.90 (3 C, NHC(*C*H_3_)_3_).^15^N‐HMBC NMR (40 MHz, benzene‐*d*_6_, ppm) *δ*: −227.65 (tmeda), −230.84 (‐NHC(CH_3_)_3_), −235.37 (−NC(CH_3_)_3_), −240.31 (NC(CH_3_)_3_, 18 H), Anal. Calc. for C_27_H_63_K_3_N_7_S_2_ (monomer) (M=667.26 g mol^−1^): C, 48.60; H, 9.52; N, 14.69; S, 9.61. Found: C, 49.45; H, 10.42; N, 14.13; S, 9.21.

**[S(*t*BuN)_3_(thf)_3_Na_3_SNa_3_(thf)_3_(N*t*Bu)_3_S] (2)**: To sodium (1.38 g, 60.14 mmol, 2.33 eq.) in thf (10 mL) S(*t*BuN)_2_ (5 mL, 25.81 mmol, 1 eq.) was slowly added at room temperature and stirred for 48 h. Instantaneously, a color change to dark red is observable and after 48 h an orange solution is obtained. Filtration via a glass fritted filter (P4) over celite and crystalizing twice from thf (6 mL) yielded the colorless product. Crystals suitable for X‐ray diffraction analysis were gained from a saturated solution of **2** in thf at −30 °C. **2** was obtained as a white solid in an overall yield of 6.81 g (72 %). ^1^H NMR (500 MHz, toluene‐*d*_8_, ppm) *δ*: 3.53 (m, 12H, OC*H*_2_CH_2_), 1.16 (s, 54H, NC(CH_3_)_3_), 1.43 (m, 12H, OCH_2_C*H*_2_). ^13^C NMR (125 MHz, toluene‐*d*_8_, ppm) *δ*: 68.04 (6 C, OC*H*_2_CH_2_), 53.08 (6 C, −*C*(CH_3_)_3_), 35.55 (36 C, −C(*C*H_3_)_3_), 25.66 (6 C, OCH_2_ *C*H_2_). ^15^N‐HMBC NMR (40 MHz, toluene‐*d*_8_, ppm) *δ*: −233.74 (*N*C(CH_3_)_3_. Anal. Calc. for C_48_H_102_ N_6_ Na_6_O_6_S_3_ (M=1093.50 g/mol^−1^): C, 52.72; H, 9.40; N, 7.69; S, 8.80. Found: C, 53.25; H, 9.82; N, 7.93; S, 8.34.

**[S(*t*BuN)_3_(thf)_3_Na_3_{(HN*t*Bu)(N*t*Bu)_2_S}] (3)**: **Method A**: To sodium (1.38 g, 60.14 mmol, 2.33 eq.) in thf (10 mL) S(*t*BuN)_2_ (5 mL, 25.81 mmol, 1 eq.) was slowly added at room temperature and stirred for 4 h. Immediately, a color change to dark red is observable. After 4 h filtration via a glass fritted filter (P4) over celite and dual crystallization from thf (6 mL) gave the red product. Crystals suitable for X‐ray diffraction analysis were gained from a saturated solution of **3** in thf at −30 °C. **3** was obtained as a red solid in a yield of 0.21 g (10 %). **Method B**: To sodium (0.27 g, 11.65 mmol, 2.33 eq.) in thf (5 mL) S(*t*BuN)_3_ (1.23 g, 5.00 mmol, 1 eq.) was slowly added at room temperature and stirred for 48 h. Promptly, a color change to dark blue is observable. After 48 h a red solution is obtained. Filtration via a glass fritted filter (P4) over celite and dual crystalizing from thf (3 mL) yielded the red product. Crystals suitable for X‐ray diffraction analysis were gained from a saturated solution of **3** in thf at −30 °C. **3** was obtained as a red solid in a yield of 0.94 g (48 %). ^1^H NMR (500 MHz, benzene‐*d*_6_, ppm) *δ*: 3.56 (m, 12H, OC*H*_2_CH_2_), 1.71 (s, 1H, N*H*C), 1.57 (s, 27H, −NHC(C*H*_3_)_3_), 1.37 (m, 12H, OCH_2_C*H*_2_), 1.31 (s, 18H, NC(C*H*_3_)_3_), 1.19 (s, 9H, NHC(C*H*_3_)_3_).^13^C NMR (125 MHz, benzene‐*d*_6_, ppm) *δ*: 67.67 (6 C, OC*H*_2_CH_2_), 53.33 (2 C, N*C*), 52.80 (3 C, N*C*), 52.25 (1 C, NH*C*), 35.22 (9 C, NC(*C*H_3_)_3_), 34.10 (6 C, NC(*C*H_3_)_3_), 31.12 (3 C, NHC(*C*H_3_)_3_), 25.26 (6 C, OCH_2_ *C*H_2_). ^15^N‐HMBC NMR (40 MHz, benzene‐*d*_6_, ppm) *δ*: −231.40 ((d, *NH*C(C*H*_3_)) −234.78 (*N*C(CH_3_)_3_), −241.66 (*N*C(CH_3_)_3_), Anal. Calc. for C_36_H_79_N_6_Na_3_O_6_S_3_ (M=777.16 g/mol^−1^):C, 55.64; H, 10.25; N, 10.81; S, 8.25. Found: C, 55.25; H, 9.72; N, 10.63; S, 8.64.

**[(tmeda)_3_S{Na_3_(N*t*Bu)_3_S}_2_] (4)**: To sodium (0.28 g, 12.03 mmol, 2.33 eq.) in a hexane/tmeda mixture (5 ml, 1 : 1) S(*t*BuN)_2_ (1 mL, 5.16 mmol, 1 eq.) was slowly added at room temperature and stirred for 48 h. Instantly, a color change to light red is observable, which faded after 12 h. Impurities of the crude product were extracted in cool hexane (6×4 mL) in a centrifuge (2 min, 8000 rpm). Crystals suitable for X‐ray diffraction analysis were gained from a saturated solution of **4** in hexane at −30 °C. **4** was obtained as a colorless solid in a yield of 1.05 g (67 %).^1^H NMR (500 MHz, toluene‐*d*_8_, ppm) *δ*: 2.23 (m, 12H, CH_2_), 2.04 (s, 36H, N(CH_3_)_2_), 1.47 (s, 18H, NC(CH_3_)_3_)), 1.46 (s, 36H, NC(CH_3_)_3_)). ^13^C NMR (125 MHz, toluene‐*d*_8_, ppm) *δ*: 58.70 (6 C, *C*H_2_), 53.43 (4 C, N*C*(*C*H_3_)_3_), 53.31 (2 C, N*C*(*C*H_3_)_3_), 46.38 (12 C, N(*C*H_3_)_2_), 39.95 (6 C, N*C*(*C*H_3_)_3_). ^15^N‐HMBC NMR (40 MHz, toluene‐*d*_8_, ppm) *δ*: −232.74 (NCH_3_, NCH_2_), −236.35 (NC(CH_3_)_3_), −58.52 (NC(CH_3_)_3_),). Anal. Calc. for C_42_H_102_Na_6_N_12_S_3_ (M=1009.48 g mol^−1^): C, 49.97; H, 10.01; N, 16.65; S, 9.84. Found: C, 49.75; H, 9.72; N, 16.63; S, 10.22.

**[(*t*BuNH)Dy{K(0.5 tmeda)}_2_{(N*t*Bu)_3_S}_2_]_n_ (5)**: To **1** in pentane (100 mg, 0.15 mmol, 1.0 eq.) DyCl_3_ (44 mg, 0.16 mmol, 1.1 eq.) was added at room temperature and stirred for 24 h. Promptly, a color change to dark blue was observed. After 24 h a yellow solution is obtained. Filtration and crystalizing twice from pentane (3 mL) yielded the white product. Crystals suitable for X‐ray diffraction analysis were gained from a saturated solution of **1** in pentane at −30 °C. Overall yield was 33 mg (24 %). ^1^H NMR (500 MHz, thf‐*d*_8_, ppm) *δ*: 63.97 (br, 36H, NC(C*H*_3_)_3_), 27.24 (br, 1H, NHC*H*_3_), 2.51 (s, 4H, CH_2_), 2.35 (s, 12H, N(C*H*_3_)_2_), −7.06 (br, 18H, ‐NC(C*H*_3_)_3_).^13^C NMR (125 MHz, thf‐*d*_8_, ppm) *δ*: signal detection was not possible due to strong signal broadening. Anal. Calc. for C_34_H_80_N_9_DyK_2_S_2_ (M=920.45 g mol^−1^): C, 44.39; H, 8.77; N, 13.70; S, 6.97. Found: C, 44.65; H, 8.82; N, 14.33; S, 7.41.

**[ClDy{Na(thf)}_2_{(N*t*Bu)_3_S}_2_] (6)**: To **2** in thf (100 mg, 91.5 μmol, 1.0 eq.) DyCl_3_ (25 mg, 91.5 μmol, 1.0 eq.) was added at room temperature and stirred for 24 h. Instantly, a color change to dark blue was observed. After 24 h a yellow solution is obtained. Centrifugation in a thf/toluene/pentane mixture (2 : 1 : 1) in 4 mL total volume and crystalizing from a thf/toluene mixture (1 : 1) in 1 mL yielded the white product. Crystals suitable for X‐ray diffraction analysis were gained from slow evaporation of pentane (3 mL) into a saturated thf (1 mL) solution of **5** at −30 °C. Overall yield was 37 mg (46 %). ^1^H NMR (500 MHz, thf‐*d*_8_, ppm) *δ*: 160.33 (bs, 36H, NC(C*H*_3_)_3_), 3.64 (m, 8H, OC*H*_2_CH_2_), 1.94 (m, 8H, OCH_2_C*H*_2_), −59.27 (bs, 18H, NC(C*H*_3_)_3_). ^13^C NMR (125 MHz, thf‐*d*_8_, ppm) *δ*: 300.11 (4 C, *C*(*C*H_3_)_3_), 151.18 67.54 (4 C, O*C*H_2_CH_2_), 24.72 (4 C, OCH_2_ *C*H_2_), (2 C, *C*(*C*H_3_)_3_), −17.04 (6 C, C(*C*H_3_)_3_), −41.50 (8 C, C(*C*H_3_)_3_). Anal. Calc. for C_32_H_70_ClDyN_6_Na_2_S_2_ (M=879.00 g/mol^−1^): C, 43.73; H, 8.03; N, 9.56; S, 7.29. Found: C, 43.45; H, 8.43; N, 9.79; S, 7.67.

**[ClEr{Na(thf)}_2_{(N*t*Bu)_3_S}_2_] (7)**: To **2** in thf (100 mg, 91.5 μmol, 1.0 eq.) ErCl_3_ (25 mg, 91.5 μmol, 1.0 eq.) was added at room temperature and stirred for 24 h. Instantaneously, a color change to dark blue was observed and after 24 h a yellow solution was obtained. Centrifugation in a thf/toluene/pentane mixture (2 : 1 : 1) in 4 mL total volume and crystalizing from a thf/toluene mixture (1 : 1) in 1 mL yielded the white product. Crystals suitable for X‐ray diffraction analysis were gained from slow evaporation of pentane (3 mL) into a saturated thf (1 mL) solution of **6** at −30 °C. Overall yield was 39 mg (48 %). ^1^H NMR (500 MHz, thf‐*d*_8_, ppm) *δ*: 15.60 (bs, 36H, NC(C*H*_3_)_3_), 3.55 (m, 8H, OC*H*_2_CH_2_), 1.54 (m, 8H, OCH_2_C*H*_2_), −38.06 (bs, 18H, NC(C*H*_3_)_3_). ^13^C NMR (125 MHz, thf‐*d*_8_, ppm) *δ*: 99.91 (12 C, C(*C*H_3_)_3_), 68.24 (4 C, O*C*H_2_CH_2_), 50.60 (4 C, *C*(*C*H_3_)_3_), 49.63 (2 C, *C*(*C*H_3_)_3_) 26.21 (4 C, OCH_2_ *C*H_2_), −34.94 (6 C, C(*C*H_3_)_3_. Anal. Calc. for C_32_H_70_ClErN_6_Na_2_S_2_ (M=883.38 g/mol^−1^): C, 43.73; H, 8.03; N, 9.56; S, 7.29. Found: C, 43.45; H, 8.43; N, 9.79; S, 7.67.

## Conflict of interest

The authors declare no conflict of interest.

1

## Supporting information

As a service to our authors and readers, this journal provides supporting information supplied by the authors. Such materials are peer reviewed and may be re‐organized for online delivery, but are not copy‐edited or typeset. Technical support issues arising from supporting information (other than missing files) should be addressed to the authors.

Supporting Information

## Data Availability

The data that support the findings of this study are available in the supplementary material of this article.
