# Supplementary material for: Alkali Metal Based Triimidosulfite Cages as Versatile Precursors for Single‐Molecule Magnets
Source: Chemistry. 2022 Feb 10;28(15):e202104470. doi: 10.1002/chem.202104470 (PMC9304269; doi:10.1002/chem.202104470)
Supplement: Supplementary file 1 — Supporting Information [file CHEM-28-0-s001.pdf]

Chemistry–A European Journal

Supporting Information

Alkali Metal Based Triimidosulfite Cages as Versatile Precursors for Single-Molecule Magnets

Daniel Lüert, Christina M. Legendre, Regine Herbst-Irmer, and Dietmar Stalke*

Table of contents

S1 Crystallographic data 2

Crystal structure of **1** 4

Crystal structure of **2** 10

Crystal structure of **3** 13

Crystal structure of **4** 19

Crystal structure of **5** 31

Crystal structure of **6** 37

Crystal structure of **7** 41

S2 Analytical details........ 45

Yield calculation procedure........ 45

NMR-spectroscopy data........ 45

Magnetic data 55

Table S10: Best fitting parameters for **5**........ 64

Table S11: Best fitting parameters for **6.**........ 64

S1 Crystallographic data

The diffraction data were collected using an Incoatec Mo or Ag Microsource[1] and a Bruker Apex II detector. The data were integrated with SAINT.[2] A multi-scan absorption correction was applied using SADABS[3]. The structures were solved by SHELXT[4] and refined on F^2 using SHELXL[5] in the graphical user interface ShelXle.[6] An overview of the crystallographic data for **1-4** can be found in **Table S1** and for **5-7** in **Table S2**, while individual bond lengths and angles are listed in **Table S3 – S9** for **1 – 7,** respectively.

Table S1. Crystal data and structure refinement for compounds **1 – 4** at 100 (2) K.

Compound	**1**	**2**	**3**	**4**
CCDC	2128424	2128425	2128426	2128427
Empirical Formula	$C_{54}H_{126}K_6N_{14}S_4$	$C_{52}H_{110}N_6Na_6O_7S_3$	$C_{36}H_{79}N_6Na_3O_3S_2$	$C_{42}H_{102}N_{12}Na_6S_3$
Formula weight	1334.52	1165.57	777.14	1009.47
Temperature (K)	100(2) K	100(2) K	100(2) K	100(2)
Wavelength (Å)	0.56086	0.56086	0.71073	0.71073
Crystal system	Monoclinic	Cubic	Orthorhombic	Monoclinic
Space group	$P2_1/c$	$Pa\bar{3}$	$Pna2_1$	$P2_1/c$
a (Å)	15.380(2)	18.917(2)	15.072(2)	19.880(3)
b (Å)	13.885(2)	18.917(2)	17.049(3)	17.651(2)
c (Å)	17.912(3)	18.917(2)	17.726(3)	36.973(3)
β (deg)	95.74(2)	90	90	90.00(2)
V (Å^3)	3806.0(10)	6770(2)	4554.9(13)	12974(3)
Z	2	4	4	8
μ/mm^{-1}	0.258	0.107	0.184	1.034
Crystal size(mm)	0.377 x 0.281 x 0.190	0.407 x 0.400 x 0.305	0.355 x 0.283 x 0.272	0.254 x 0.212 x 0.145
Θ max (deg)	20.252	19.994	26.715	25.693
Reflections collected	141047	32527	73524	126730
Independent reflections	7484	2148	8681	24641
R_{int}	0.0722	0.0722	0.0414	0.0655
Data/restraints/ parameters	7484 / 0 / 376	2148 / 306 / 190	8681 / 2399 / 684	24641 / 729 /127055
$R1^a$ ($I > 2\sigma(I)$)	0.0296	0.0370	0.0454	0.0456
$wR2^b$(all data)	0.0722	0.0942	0.1193	0.1078
Absolute structure parameter	-	-	0.23(11)	-
$\Delta\rho_{max}/\Delta\rho_{min}$(e Å^{-3})	0.306/-0.353	0.250/-0.225	0.397/-0.212	0.328/-0.304
Shape and color	red blocks	colorless blocks	red blocks	colorless blocks

$^aR1 = \Sigma||F_0| - |F_c||/\Sigma|F_o|$. $^bwR_2 = [\Sigma w(F_o^2 - F_c^2)^2/\Sigma(F_o^2)^2]^{1/2}$

Table S2. Crystal data and structure refinement for compounds **5 – 7** at 100 (2) K.

Compound	**5**	**6**	**7**
CCDC	2128428	2128429	2128430
Empirical Formula	$C_{78}H_{184}Dy_2K_4N_{18}S_4$	$C_{32}H_{70}$ $ClDyN_6Na_2O_2S_2$	$C_{32}H_{70}ClErN_6Na_2O_2S_2$
Formula weight	1984.06	878.99	883.75
Temperature (K)	100(2) K	100(2) K	100(2) K
Wavelength (Å)	0.56086	0.71073	0.56086
Crystal system	Triclinic	Monoclinic	Monoclinic
Space group	$P\bar{1}$	$C2$/c	$C2$/c
a (Å)	11.149(2)	11.858(2)	11.822(2)
b (Å)	12.947(2)	18.318(2)	18.247(3)
c (Å)	18.319(3)	19.385(3)	19.388(2)
α (deg)	82.43(2)	90	90
β (deg)	79.11(2)	90.26(2)	90.41(2)
γ (deg)	82.64(3)	90	90
V (Å^3)	2559.5(8)	4210.7(11)	4182.2(11)
Z	1	4	4
μ/mm^{-1}	0.930	1.992	1.194
Crystal size(mm)	0.241 x 0.231 x 0.184	0.355 x 0.138 x 0.134	0.454 x 0.430 x 0.360
Θ max (deg)	21.787	26.796	21.932
Reflections collected	150125	60115	65058
Independent reflections	12369	4490	5148
R_{int}	0.0406	0.0193	0.0370
Data/restraints/ parameters	12369 / 250 / 558	4490 / 0 / 218	5148 / 0 / 218
$R1^a$ ($I > 2\sigma(I)$)	0.0227	0.0152	0.0210
$wR2^b$(all data)	0.0524	0.0405	0.0546
$\Delta\rho_{max}/\Delta\rho_{min}$(e Å^{-3})	2.483/-1.160	0.789/-0.272	1.523/-1.219
Shape and color	colorless blocks	colorless blocks	colorless blocks

$^aR1 = \Sigma||F_o| - |F_c||/\Sigma|F_o|$. $^bwR_2 = [\Sigma w(F_o^2 - F_c^2)^2/\Sigma(F_o^2)^2]^{1/2}$

Crystal structure of **1**

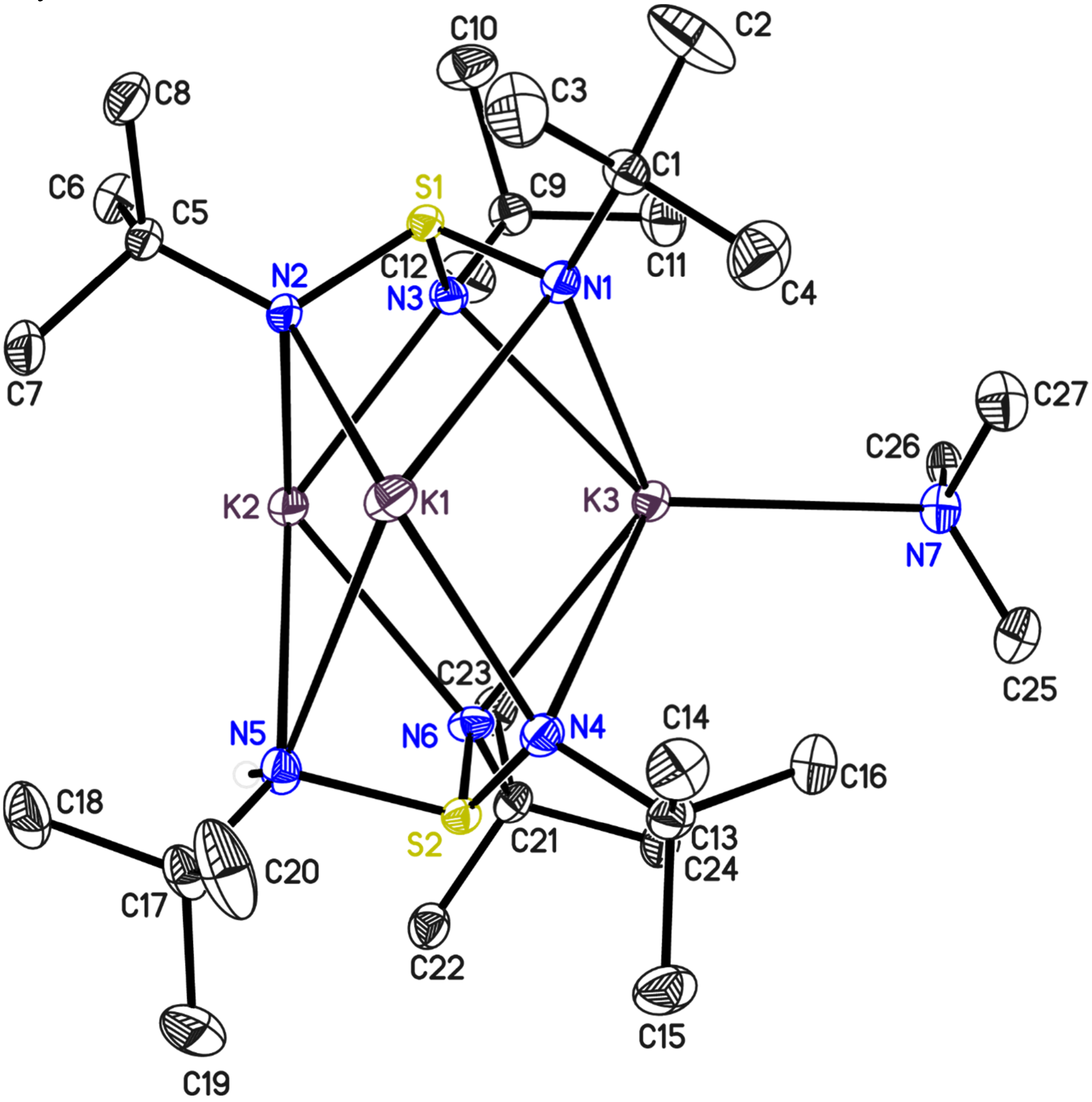

Figure S1. Asymmetric unit of **1** with thermal ellipsoids at 50% probability level. The hydrogen atoms are omitted for clarity, except the Hydrogen at N5.

Half a molecule of the bridging tetramethylethylenediamine moiety at K(3) is located in the asymmetric unit and connects two complex molecules. No disorder treatment is required for *t*Butyl groups as it mandatory for complex **2** and **3**. The hydrogen atom associated with N(5) is refined freely.

Table S3. Bond lengths [Å] and angles [°] for **1**.

K(1)-N(2)	2.6164(14)	N(7)-K(3)-C(16)	77.27(4)
K(1)-N(4)	2.6975(14)	N(4)-K(3)-C(16)	44.70(4)
K(1)-N(1)	2.8403(14)	N(1)-K(3)-C(11)	72.17(5)
K(1)-N(5)	3.2356(16)	N(6)-K(3)-C(11)	123.92(4)
K(1)-S(1)	3.4335(8)	N(3)-K(3)-C(11)	45.59(4)
K(1)-C(20)	3.530(2)	N(7)-K(3)-C(11)	78.18(4)
K(1)-S(2)	3.6783(7)	N(4)-K(3)-C(11)	160.41(4)
K(1)-K(3)	3.7519(9)	C(16)-K(3)-C(11)	151.81(4)
K(1)-K(2)	3.9473(7)	N(1)-K(3)-S(1)	27.72(3)
K(2)-N(3)	2.6001(14)	N(6)-K(3)-S(1)	103.95(3)
K(2)-N(2)	2.7482(15)	N(3)-K(3)-S(1)	28.46(3)
K(2)-N(6)	2.8025(14)	N(7)-K(3)-S(1)	131.98(3)
K(2)-C(8)#1	3.1194(18)	N(4)-K(3)-S(1)	100.99(3)
K(2)-N(5)	3.1923(15)	C(16)-K(3)-S(1)	132.97(3)
K(2)-S(1)	3.3709(7)	C(11)-K(3)-S(1)	59.86(3)
K(2)-C(5)	3.4165(17)	N(1)-K(3)-C(27)	97.20(4)
K(2)-C(6)	3.4417(18)	N(6)-K(3)-C(27)	142.17(4)
K(2)-K(3)	3.6449(7)	N(3)-K(3)-C(27)	116.53(4)
K(2)-S(2)	3.7727(9)	N(7)-K(3)-C(27)	24.54(4)
K(2)-H(1)	2.858(19)	N(4)-K(3)-C(27)	122.32(4)
K(3)-N(1)	2.6655(14)	C(16)-K(3)-C(27)	78.87(5)
K(3)-N(6)	2.8569(15)	C(11)-K(3)-C(27)	73.04(5)
K(3)-N(3)	2.9429(14)	S(1)-K(3)-C(27)	113.30(4)
K(3)-N(7)	3.0495(15)	N(1)-K(3)-S(2)	109.02(3)
K(3)-N(4)	3.0533(15)	N(6)-K(3)-S(2)	26.45(3)
K(3)-C(16)	3.381(2)	N(3)-K(3)-S(2)	107.30(3)
K(3)-C(11)	3.408(2)	N(7)-K(3)-S(2)	122.34(3)
K(3)-S(1)	3.4416(6)	N(4)-K(3)-S(2)	26.51(3)
K(3)-C(27)	3.501(2)	C(16)-K(3)-S(2)	59.66(3)
K(3)-S(2)	3.5493(7)	C(11)-K(3)-S(2)	147.89(3)
N(2)-C(5)	1.470(2)	S(1)-K(3)-S(2)	105.661(19)
N(2)-S(1)	1.6548(14)	C(27)-K(3)-S(2)	136.15(4)
N(3)-C(9)	1.468(2)	N(1)-K(3)-K(2)	79.94(3)
N(3)-S(1)	1.6422(14)	N(6)-K(3)-K(2)	49.25(3)
N(6)-C(21)	1.482(2)	N(3)-K(3)-K(2)	44.94(3)
N(6)-S(2)	1.6133(14)	N(7)-K(3)-K(2)	147.08(3)
N(7)-C(27)	1.460(2)	N(4)-K(3)-K(2)	78.94(3)

N(7)-C(25)	1.463(2)	C(16)-K(3)-K(2)	122.06(3)
N(7)-C(26)	1.464(2)	C(11)-K(3)-K(2)	86.13(4)
S(1)-N(1)	1.6454(14)	S(1)-K(3)-K(2)	56.720(14)
N(1)-C(1)	1.472(2)	C(27)-K(3)-K(2)	158.74(3)
C(1)-C(4)	1.525(2)	S(2)-K(3)-K(2)	63.239(17)
C(1)-C(2)	1.526(2)	N(1)-K(3)-K(1)	49.03(3)
C(1)-C(3)	1.526(2)	N(6)-K(3)-K(1)	77.49(3)
C(5)-C(8)	1.531(2)	N(3)-K(3)-K(1)	78.46(3)
C(5)-C(7)	1.532(2)	N(7)-K(3)-K(1)	148.36(3)
C(5)-C(6)	1.538(2)	N(4)-K(3)-K(1)	45.28(3)
C(9)-C(12)	1.525(2)	C(16)-K(3)-K(1)	79.67(3)
C(9)-C(11)	1.537(2)	C(11)-K(3)-K(1)	116.43(3)
C(9)-C(10)	1.537(2)	S(1)-K(3)-K(1)	56.822(16)
N(4)-C(13)	1.487(2)	C(27)-K(3)-K(1)	128.72(3)
N(4)-S(2)	1.5891(14)	S(2)-K(3)-K(1)	60.426(15)
C(13)-C(14)	1.525(2)	K(2)-K(3)-K(1)	64.486(17)
C(13)-C(15)	1.528(2)	C(5)-N(2)-S(1)	115.08(10)
C(13)-C(16)	1.531(2)	C(5)-N(2)-K(1)	132.96(10)
S(2)-N(5)	1.7594(15)	S(1)-N(2)-K(1)	104.75(6)
N(5)-H(1)	0.88(2)	C(5)-N(2)-K(2)	104.02(9)
N(5)-C(17)	1.492(2)	S(1)-N(2)-K(2)	96.77(6)
C(17)-C(20)	1.511(3)	K(1)-N(2)-K(2)	94.72(4)
C(17)-C(18)	1.523(2)	C(9)-N(3)-S(1)	117.65(11)
C(17)-C(19)	1.526(3)	C(9)-N(3)-K(2)	136.35(10)
C(21)-C(23)	1.523(2)	S(1)-N(3)-K(2)	102.89(6)
C(21)-C(22)	1.530(2)	C(9)-N(3)-K(3)	109.88(9)
C(21)-C(24)	1.536(2)	S(1)-N(3)-K(3)	92.89(6)
C(26)-C(26)#2	1.513(3)	K(2)-N(3)-K(3)	81.98(4)
N(2)-K(1)-N(4)	118.99(4)	C(21)-N(6)-S(2)	114.20(10)
N(2)-K(1)-N(1)	56.20(4)	C(21)-N(6)-K(2)	123.07(10)
N(4)-K(1)-N(1)	96.84(4)	S(2)-N(6)-K(2)	114.69(7)
N(2)-K(1)-N(5)	91.82(4)	C(21)-N(6)-K(3)	116.16(9)
N(4)-K(1)-N(5)	50.03(4)	S(2)-N(6)-K(3)	101.47(6)
N(1)-K(1)-N(5)	117.42(4)	K(2)-N(6)-K(3)	80.18(4)
N(2)-K(1)-S(1)	27.78(3)	C(27)-N(7)-C(25)	109.00(14)
N(4)-K(1)-S(1)	109.26(3)	C(27)-N(7)-C(26)	109.58(14)
N(1)-K(1)-S(1)	28.45(3)	C(25)-N(7)-C(26)	110.58(13)
N(5)-K(1)-S(1)	105.18(3)	C(27)-N(7)-K(3)	95.30(10)

N(2)-K(1)-C(20)	123.20(5)	C(25)-N(7)-K(3)	115.57(10)
N(4)-K(1)-C(20)	62.89(5)	C(26)-N(7)-K(3)	115.53(10)
N(1)-K(1)-C(20)	157.52(5)	N(3)-S(1)-N(1)	105.51(7)
N(5)-K(1)-C(20)	42.59(4)	N(3)-S(1)-N(2)	102.01(7)
S(1)-K(1)-C(20)	145.60(3)	N(1)-S(1)-N(2)	102.72(7)
N(2)-K(1)-S(2)	102.96(3)	N(3)-S(1)-K(2)	48.75(5)
N(4)-K(1)-S(2)	22.90(3)	N(1)-S(1)-K(2)	105.30(5)
N(1)-K(1)-S(2)	101.79(3)	N(2)-S(1)-K(2)	54.06(5)
N(5)-K(1)-S(2)	28.58(3)	N(3)-S(1)-K(1)	109.87(5)
S(1)-K(1)-S(2)	103.086(15)	N(1)-S(1)-K(1)	55.32(5)
C(20)-K(1)-S(2)	55.74(4)	N(2)-S(1)-K(1)	47.47(5)
N(2)-K(1)-K(3)	76.17(3)	K(2)-S(1)-K(1)	70.911(12)
N(4)-K(1)-K(3)	53.53(3)	N(3)-S(1)-K(3)	58.65(5)
N(1)-K(1)-K(3)	45.12(3)	N(1)-S(1)-K(3)	48.89(5)
N(5)-K(1)-K(3)	78.26(3)	N(2)-S(1)-K(3)	98.31(5)
S(1)-K(1)-K(3)	57.030(14)	K(2)-S(1)-K(3)	64.682(13)
C(20)-K(1)-K(3)	112.61(4)	K(1)-S(1)-K(3)	66.148(19)
S(2)-K(1)-K(3)	57.059(11)	C(1)-N(1)-S(1)	115.39(11)
N(2)-K(1)-K(2)	43.94(3)	C(1)-N(1)-K(3)	136.41(10)
N(4)-K(1)-K(2)	77.74(3)	S(1)-N(1)-K(3)	103.40(6)
N(1)-K(1)-K(2)	72.75(3)	C(1)-N(1)-K(1)	107.90(9)
N(5)-K(1)-K(2)	51.62(3)	S(1)-N(1)-K(1)	96.23(6)
S(1)-K(1)-K(2)	53.805(14)	K(3)-N(1)-K(1)	85.85(4)
C(20)-K(1)-K(2)	92.38(4)	N(1)-C(1)-C(4)	105.46(14)
S(2)-K(1)-K(2)	59.179(14)	N(1)-C(1)-C(2)	112.98(14)
K(3)-K(1)-K(2)	56.443(12)	C(4)-C(1)-C(2)	107.67(16)
N(3)-K(2)-N(2)	57.17(4)	N(1)-C(1)-C(3)	114.60(14)
N(3)-K(2)-N(6)	102.43(4)	C(4)-C(1)-C(3)	107.60(16)
N(2)-K(2)-N(6)	113.33(4)	C(2)-C(1)-C(3)	108.18(17)
N(3)-K(2)-C(8)#1	157.64(5)	N(2)-C(5)-C(8)	111.64(14)
N(2)-K(2)-C(8)#1	134.69(5)	N(2)-C(5)-C(7)	105.77(13)
N(6)-K(2)-C(8)#1	89.53(4)	C(8)-C(5)-C(7)	107.47(14)
N(3)-K(2)-N(5)	126.31(4)	N(2)-C(5)-C(6)	115.50(13)
N(2)-K(2)-N(5)	90.36(4)	C(8)-C(5)-C(6)	108.21(14)
N(6)-K(2)-N(5)	48.85(4)	C(7)-C(5)-C(6)	107.88(14)
C(8)#1-K(2)-N(5)	75.69(4)	N(2)-C(5)-K(2)	51.30(7)
N(3)-K(2)-S(1)	28.35(3)	C(8)-C(5)-K(2)	161.44(11)
N(2)-K(2)-S(1)	29.18(3)	C(7)-C(5)-K(2)	86.39(10)

N(6)-K(2)-S(1)	107.02(3)	C(6)-C(5)-K(2)	77.96(9)
C(8)#1-K(2)-S(1)	160.79(4)	C(5)-C(6)-K(2)	76.13(9)
N(5)-K(2)-S(1)	107.64(3)	C(5)-C(8)-K(2)#3	169.19(12)
N(3)-K(2)-C(5)	68.53(4)	N(3)-C(9)-C(12)	105.44(14)
N(2)-K(2)-C(5)	24.68(4)	N(3)-C(9)-C(11)	112.45(14)
N(6)-K(2)-C(5)	135.83(4)	C(12)-C(9)-C(11)	108.19(14)
C(8)#1-K(2)-C(5)	115.26(5)	N(3)-C(9)-C(10)	114.65(14)
N(5)-K(2)-C(5)	100.25(4)	C(12)-C(9)-C(10)	108.14(15)
S(1)-K(2)-C(5)	45.75(3)	C(11)-C(9)-C(10)	107.71(15)
N(3)-K(2)-C(6)	66.41(4)	C(9)-C(11)-K(3)	88.77(10)
N(2)-K(2)-C(6)	46.91(4)	C(13)-N(4)-S(2)	116.78(11)
N(6)-K(2)-C(6)	160.11(4)	C(13)-N(4)-K(1)	124.82(10)
C(8)#1-K(2)-C(6)	106.76(5)	S(2)-N(4)-K(1)	115.77(7)
N(5)-K(2)-C(6)	123.34(4)	C(13)-N(4)-K(3)	110.05(10)
S(1)-K(2)-C(6)	55.17(3)	S(2)-N(4)-K(3)	94.43(6)
C(5)-K(2)-C(6)	25.92(4)	K(1)-N(4)-K(3)	81.19(4)
N(3)-K(2)-K(3)	53.08(3)	N(4)-C(13)-C(14)	106.00(14)
N(2)-K(2)-K(3)	76.70(3)	N(4)-C(13)-C(15)	115.75(14)
N(6)-K(2)-K(3)	50.56(3)	C(14)-C(13)-C(15)	109.11(14)
C(8)#1-K(2)-K(3)	139.60(3)	N(4)-C(13)-C(16)	109.53(13)
N(5)-K(2)-K(3)	80.42(3)	C(14)-C(13)-C(16)	108.35(14)
S(1)-K(2)-K(3)	58.598(11)	C(15)-C(13)-C(16)	107.89(15)
C(5)-K(2)-K(3)	100.63(3)	C(13)-C(16)-K(3)	94.83(10)
C(6)-K(2)-K(3)	113.58(3)	N(4)-S(2)-N(6)	110.83(7)
N(3)-K(2)-S(2)	109.28(3)	N(4)-S(2)-N(5)	99.39(7)
N(2)-K(2)-S(2)	98.06(3)	N(6)-S(2)-N(5)	95.78(7)
N(6)-K(2)-S(2)	22.86(3)	N(4)-S(2)-K(3)	59.06(5)
C(8)#1-K(2)-S(2)	89.07(3)	N(6)-S(2)-K(3)	52.08(5)
N(5)-K(2)-S(2)	27.69(3)	N(5)-S(2)-K(3)	108.25(5)
S(1)-K(2)-S(2)	102.354(13)	N(4)-S(2)-K(1)	41.33(5)
C(5)-K(2)-S(2)	116.68(3)	N(6)-S(2)-K(1)	97.08(5)
C(6)-K(2)-S(2)	142.58(3)	N(5)-S(2)-K(1)	61.60(5)
K(3)-K(2)-S(2)	57.144(14)	K(3)-S(2)-K(1)	62.515(15)
N(3)-K(2)-K(1)	78.70(3)	N(4)-S(2)-K(2)	97.57(5)
N(2)-K(2)-K(1)	41.34(3)	N(6)-S(2)-K(2)	42.45(5)
N(6)-K(2)-K(1)	74.70(3)	N(5)-S(2)-K(2)	57.47(5)
C(8)#1-K(2)-K(1)	123.07(4)	K(3)-S(2)-K(2)	59.617(14)
N(5)-K(2)-K(1)	52.61(3)	K(1)-S(2)-K(2)	63.966(11)

S(1)-K(2)-K(1)	55.285(13)	H(1)-N(5)-C(17)	105.6(13)
C(5)-K(2)-K(1)	61.18(3)	H(1)-N(5)-S(2)	101.1(13)
C(6)-K(2)-K(1)	86.66(3)	C(17)-N(5)-S(2)	119.15(11)
K(3)-K(2)-K(1)	59.070(16)	H(1)-N(5)-K(2)	60.2(13)
S(2)-K(2)-K(1)	56.855(14)	C(17)-N(5)-K(2)	145.75(10)
N(3)-K(2)-H(1)	140.5(4)	S(2)-N(5)-K(2)	94.83(6)
N(2)-K(2)-H(1)	104.2(4)	H(1)-N(5)-K(1)	135.2(13)
N(6)-K(2)-H(1)	49.5(4)	C(17)-N(5)-K(1)	106.37(10)
C(8)#1-K(2)-H(1)	60.9(4)	S(2)-N(5)-K(1)	89.82(6)
N(5)-K(2)-H(1)	15.5(4)	K(2)-N(5)-K(1)	75.77(3)
S(1)-K(2)-H(1)	123.1(4)	H(1)-N(5)-H(1)	0(3)
C(5)-K(2)-H(1)	110.0(4)	C(17)-N(5)-H(1)	105.6(13)
C(6)-K(2)-H(1)	129.3(4)	S(2)-N(5)-H(1)	101.1(13)
K(3)-K(2)-H(1)	90.6(4)	K(2)-N(5)-H(1)	60.2(13)
S(2)-K(2)-H(1)	33.7(4)	K(1)-N(5)-H(1)	135.2(13)
K(1)-K(2)-H(1)	68.0(4)	N(5)-C(17)-C(20)	110.83(15)
N(1)-K(3)-N(6)	119.63(4)	N(5)-C(17)-C(18)	105.48(14)
N(1)-K(3)-N(3)	55.36(4)	C(20)-C(17)-C(18)	109.11(16)
N(6)-K(3)-N(3)	93.16(4)	N(5)-C(17)-C(19)	111.75(15)
N(1)-K(3)-N(7)	121.10(4)	C(20)-C(17)-C(19)	110.84(17)
N(6)-K(3)-N(7)	119.21(4)	C(18)-C(17)-C(19)	108.63(16)
N(3)-K(3)-N(7)	123.41(4)	C(17)-C(20)-K(1)	93.61(11)
N(1)-K(3)-N(4)	92.61(4)	N(6)-C(21)-C(23)	105.52(13)
N(6)-K(3)-N(4)	52.86(4)	N(6)-C(21)-C(22)	113.61(13)
N(3)-K(3)-N(4)	115.57(4)	C(23)-C(21)-C(22)	108.18(14)
N(7)-K(3)-N(4)	121.00(4)	N(6)-C(21)-C(24)	111.68(13)
N(1)-K(3)-C(16)	109.66(4)	C(23)-C(21)-C(24)	108.54(14)
N(6)-K(3)-C(16)	80.69(4)	C(22)-C(21)-C(24)	109.09(14)
N(3)-K(3)-C(16)	158.08(4)	N(7)-C(26)-C(26)#2	112.05(17)

Crystal structure of **2**

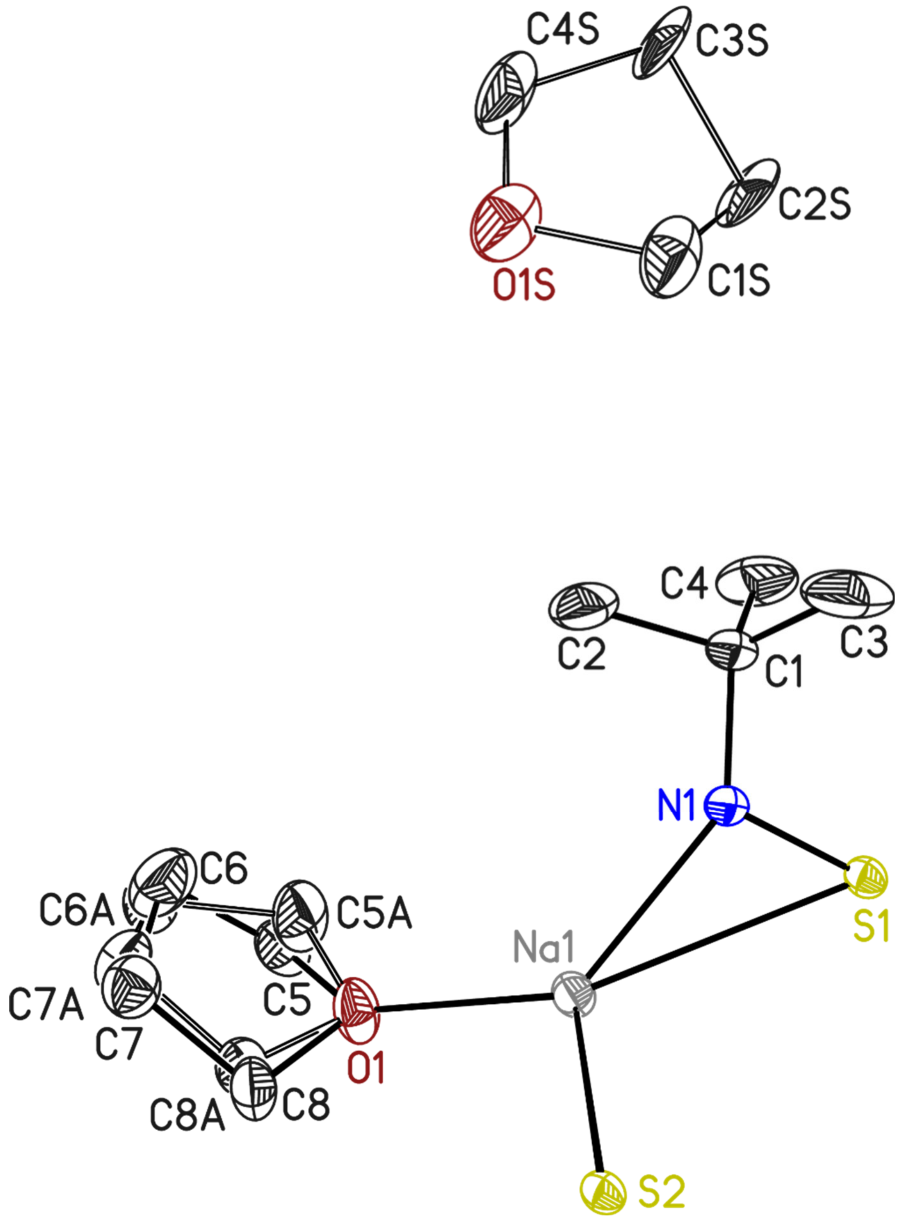

Figure S2. Asymmetric unit of **2** with thermal ellipsoids at 50% probability level. The hydrogen atoms are omitted for clarity.

The asymmetric unit shows one sixth of the entire molecule. The THF molecule is disordered over two positions and restraints on the anisotropic displacement parameters and on distances and angle distances were applied. The occupancy of the minor position refines to 0.365(16). The S2 bridges between the two triimidosulfite moieties and coordinates three sodium ions on each side. An additional THF molecule is found in the lattice (O(1S) unit), which is disordered about a $\overline{3}$ position.

Table S4. Bond lengths [Å] and angles [°] for **2**.

S(1)-N(1)	1.6510(15)	N(1)-Na(1)-Na(1)#1	50.92(4)
S(1)-Na(1)	3.0314(10)	N(1)#2-Na(1)-Na(1)#1	81.96(4)
Na(1)-O(1)	2.3135(15)	S(2)-Na(1)-Na(1)#1	55.220(11)
Na(1)-N(1)	2.3300(17)	S(1)-Na(1)-Na(1)#1	58.596(13)
Na(1)-N(1)#2	2.4756(17)	O(1)-Na(1)-Na(1)#2	140.16(5)
Na(1)-S(2)	2.7691(8)	N(1)-Na(1)-Na(1)#2	84.20(4)
Na(1)-Na(1)#1	3.1592(13)	N(1)#2-Na(1)-Na(1)#2	46.94(4)
C(1)-N(1)	1.479(2)	Na(1)#1-Na(1)-Na(1)#2	60.0
C(1)-C(2)	1.517(3)	N(1)-C(1)-C(2)	104.92(16)
C(1)-C(4)	1.521(3)	N(1)-C(1)-C(4)	113.11(18)
C(1)-C(3)	1.527(3)	C(2)-C(1)-C(4)	108.1(2)
O(1)-C(5)	1.426(6)	N(1)-C(1)-C(3)	113.47(17)
O(1)-C(5A)	1.434(10)	C(2)-C(1)-C(3)	109.2(2)
O(1)-C(8A)	1.438(12)	C(4)-C(1)-C(3)	107.78(19)
O(1)-C(8)	1.442(7)	C(1)-N(1)-S(1)	115.35(13)
C(5)-C(6)	1.559(8)	C(1)-N(1)-Na(1)	143.20(12)
C(6)-C(7)	1.530(7)	S(1)-N(1)-Na(1)	97.73(7)
C(7)-C(8)	1.517(7)	C(1)-N(1)-Na(1)#1	110.62(12)
C(5A)-C(6A)	1.509(12)	S(1)-N(1)-Na(1)#1	92.35(7)
C(6A)-C(7A)	1.523(11)	Na(1)-N(1)-Na(1)#1	82.14(6)
C(7A)-C(8A)	1.516(11)	Na(1)#3-S(2)-Na(1)#4	69.56(2)
O(1S)-C(1S)	1.456(12)	Na(1)#3-S(2)-Na(1)#2	110.44(2)
O(1S)-C(4S)	1.490(13)	Na(1)#4-S(2)-Na(1)#2	180.0
C(1S)-C(2S)	1.515(14)	Na(1)#3-S(2)-Na(1)#1	110.44(2)
C(2S)-C(3S)	1.551(15)	C(5A)-O(1)-C(8A)	106.5(8)
C(3S)-C(4S)	1.508(14)	C(5)-O(1)-C(8)	104.8(6)
N(1)-S(1)-N(1)#1	103.01(7)	C(5)-O(1)-Na(1)	139.6(3)
N(1)-S(1)-Na(1)#2	101.56(6)	C(5A)-O(1)-Na(1)	126.5(6)
N(1)-S(1)-Na(1)	49.61(6)	C(8A)-O(1)-Na(1)	117.7(10)
N(1)#1-S(1)-Na(1)	101.55(6)	C(8)-O(1)-Na(1)	113.9(6)
N(1)#2-S(1)-Na(1)	54.68(6)	O(1)-C(5)-C(6)	100.5(5)
Na(1)#1-S(1)-Na(1)	62.81(3)	C(7)-C(6)-C(5)	103.4(5)
O(1)-Na(1)-N(1)	134.21(6)	C(8)-C(7)-C(6)	104.0(5)
O(1)-Na(1)-N(1)#2	130.53(6)	O(1)-C(8)-C(7)	104.3(6)
N(1)-Na(1)-N(1)#2	64.98(7)	O(1)-C(5A)-C(6A)	110.7(8)
O(1)-Na(1)-S(2)	110.01(4)	C(5A)-C(6A)-C(7A)	102.7(8)
N(1)-Na(1)-S(2)	105.99(5)	C(8A)-C(7A)-C(6A)	102.8(10)

N(1)#2-Na(1)-S(2)	102.02(4)	O(1)-C(8A)-C(7A)	107.8(9)
O(1)-Na(1)-S(1)	147.86(5)	C(1S)-O(1S)-C(4S)	99.9(10)
N(1)-Na(1)-S(1)	32.66(4)	O(1S)-C(1S)-C(2S)	112.6(11)
N(1)#2-Na(1)-S(1)	32.97(4)	C(1S)-C(2S)-C(3S)	98.4(11)
S(2)-Na(1)-S(1)	101.81(2)	C(4S)-C(3S)-C(2S)	100.3(11)
O(1)-Na(1)-Na(1)#1	147.48(5)	O(1S)-C(4S)-C(3S)	112.4(13)

Symmetry transformations used to generate equivalent atoms:

#1 z+1/2,-x+3/2,-y+1   #2 -y+3/2,-z+1,x-1/2   #3 -x+2,-y+1,-z+1

#4 y+1/2,z,-x+3/2   #5 -z+3/2,x-1/2,y

Crystal structure of **3**

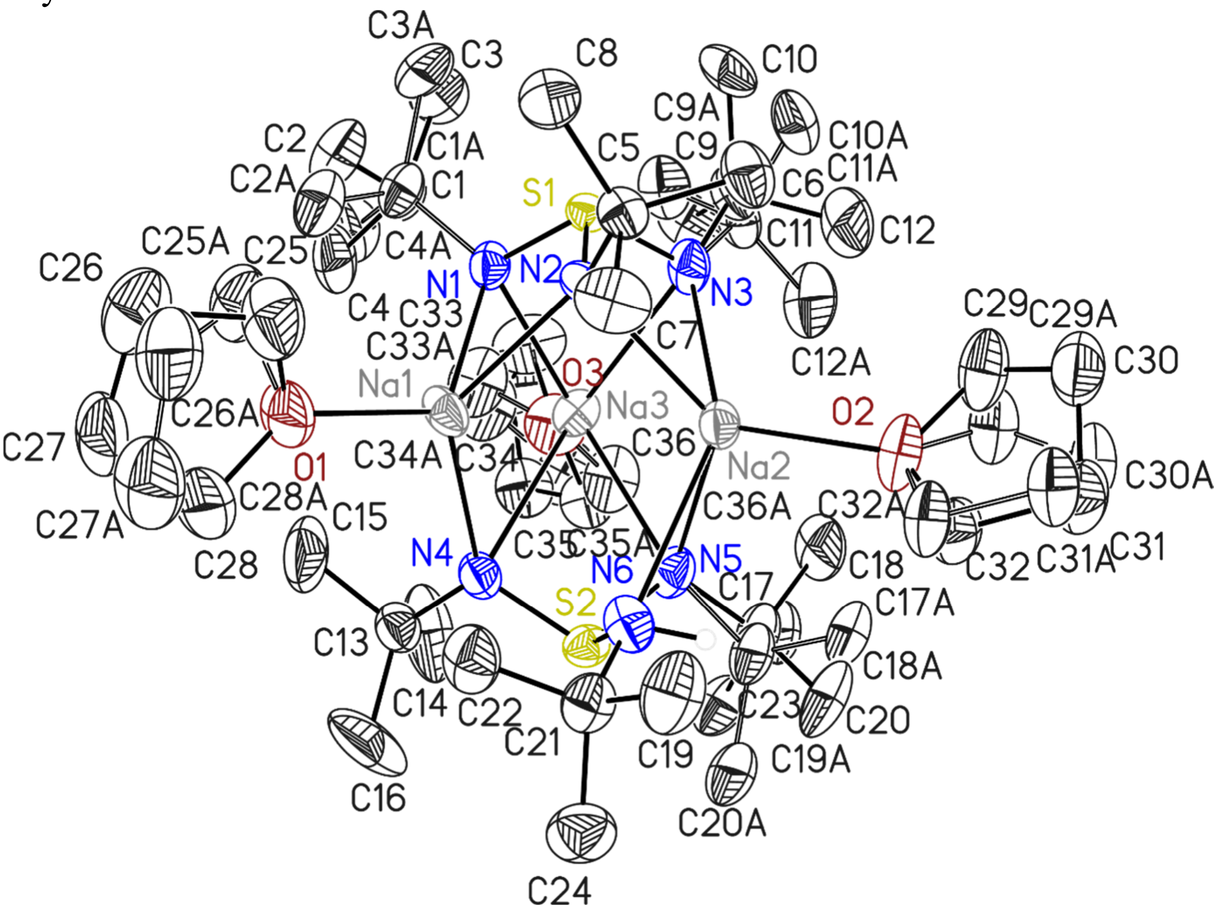

Figure S3. Asymmetric unit of **3** with thermal ellipsoids at 50% probability level. The hydrogen atoms are omitted for clarity, except the hydrogen at N6.

The asymmetric unit shows one entire molecule. Three of the six *t*Butyl-groups and all THF molecules are disordered over two positions and restraints, on the anisotropic displacement parameters and on distances and angle distances, were applied for these groups. The occupancies of the minor positions refine to 0.41(3), 0.232(10), 0.129(7), 0.380(11), 0.241(9) and 0.426(14), respectively. The hydrogen atom associated with N(6) is refined with a distance restraint.

Table S5. Bond lengths [Å] and angles [°] for **3**.

Na(1)-O(1)	2.325(3)
Na(1)-N(2)	2.340(4)
Na(1)-N(4)	2.441(4)
Na(1)-N(1)	2.575(4)
Na(1)-C(2A)	3.100(17)
Na(1)-S(1)	3.1131(18)
Na(1)-Na(3)	3.160(2)
Na(1)-Na(2)	3.335(2)
Na(2)-O(2)	2.356(3)
Na(2)-N(3)	2.440(4)
Na(2)-N(2)	2.472(4)
Na(2)-N(5)	2.612(5)
Na(2)-N(6)	2.705(4)
Na(2)-Na(3)	3.1509(19)
Na(2)-S(1)	3.155(2)
Na(2)-S(2)	3.359(2)
Na(3)-O(3)	2.384(3)
Na(3)-N(1)	2.394(4)
Na(3)-N(5)	2.538(4)
Na(3)-N(3)	2.643(4)
Na(3)-N(4)	2.789(4)
Na(3)-S(1)	3.1412(19)
Na(3)-S(2)	3.345(2)
S(1)-N(3)	1.586(4)
S(1)-N(1)	1.647(4)
S(1)-N(2)	1.657(3)
N(1)-C(1)	1.481(16)
N(1)-C(1A)	1.493(12)
C(1)-C(2)	1.514(17)
C(1)-C(4)	1.532(17)
C(1)-C(3)	1.558(17)
N(2)-C(5)	1.487(5)
C(5)-C(6)	1.508(6)
C(5)-C(7)	1.547(6)
C(5)-C(8)	1.556(6)
N(3)-C(9A)	1.466(18)
C(32)-O(2)-C(29)	108.6(5)
N(3)-C(9)	1.498(7)
C(9)-C(12)	1.510(10)
C(9)-C(11)	1.541(10)
C(9)-C(10)	1.568(9)
S(2)-N(5)	1.603(4)
S(2)-N(4)	1.604(4)
S(2)-N(6)	1.732(4)
N(4)-C(13)	1.499(6)
C(13)-C(14)	1.470(7)
C(13)-C(15)	1.504(8)
C(13)-C(16)	1.551(8)
N(5)-C(17)	1.473(6)
N(5)-C(17A)	1.537(19)
C(17)-C(19)	1.517(8)
C(17)-C(18)	1.522(8)
C(17)-C(20)	1.559(8)
N(6)-H(1)	0.94(3)
N(6)-C(21)	1.470(6)
C(21)-C(22)	1.487(7)
C(21)-C(23)	1.526(7)
C(21)-C(24)	1.574(7)
C(9A)-C(10A)	1.521(18)
C(9A)-C(12A)	1.527(18)
C(9A)-C(11A)	1.528(19)
C(1A)-C(2A)	1.513(13)
C(1A)-C(4A)	1.524(14)
C(1A)-C(3A)	1.563(14)
C(17A)-C(19A)	1.52(2)
C(17A)-C(18A)	1.54(2)
C(17A)-C(20A)	1.56(2)
O(1)-C(28A)	1.419(17)
O(1)-C(28)	1.425(12)
O(1)-C(25A)	1.473(15)
O(1)-C(25)	1.492(10)
C(25)-C(26)	1.524(13)

C(26)-C(27)	1.435(14)	N(1)-Na(1)-S(1)	31.90(8)
C(27)-C(28)	1.508(15)	C(2A)-Na(1)-S(1)	64.3(3)
O(2)-C(29A)	1.396(16)	O(1)-Na(1)-Na(3)	160.79(14)
O(2)-C(32A)	1.450(18)	N(2)-Na(1)-Na(3)	81.64(9)
O(2)-C(32)	1.460(9)	N(4)-Na(1)-Na(3)	58.05(9)
O(2)-C(29)	1.468(8)	N(1)-Na(1)-Na(3)	48.03(9)
C(29)-C(30)	1.478(9)	C(2A)-Na(1)-Na(3)	93.7(2)
C(30)-C(31)	1.541(12)	S(1)-Na(1)-Na(3)	60.10(4)
C(31)-C(32)	1.479(10)	O(1)-Na(1)-Na(2)	141.09(14)
O(3)-C(36A)	1.382(15)	N(2)-Na(1)-Na(2)	47.78(9)
O(3)-C(33A)	1.389(12)	N(4)-Na(1)-Na(2)	77.77(10)
O(3)-C(36)	1.448(11)	N(1)-Na(1)-Na(2)	79.76(9)
O(3)-C(33)	1.506(10)	C(2A)-Na(1)-Na(2)	122.8(3)
C(33)-C(34)	1.441(13)	S(1)-Na(1)-Na(2)	58.47(4)
C(34)-C(35)	1.523(14)	Na(3)-Na(1)-Na(2)	57.97(4)
C(35)-C(36)	1.507(13)	O(2)-Na(2)-N(3)	118.90(14)
C(25A)-C(26A)	1.539(17)	O(2)-Na(2)-N(2)	124.64(14)
C(26A)-C(27A)	1.479(18)	N(3)-Na(2)-N(2)	60.34(11)
C(27A)-C(28A)	1.507(18)	O(2)-Na(2)-N(5)	111.47(14)
C(29A)-C(30A)	1.484(18)	N(3)-Na(2)-N(5)	100.97(14)
C(30A)-C(31A)	1.486(19)	N(2)-Na(2)-N(5)	123.24(12)
C(31A)-C(32A)	1.479(19)	O(2)-Na(2)-N(6)	97.82(15)
C(33A)-C(34A)	1.483(15)	N(3)-Na(2)-N(6)	142.64(14)
C(34A)-C(35A)	1.486(17)	N(2)-Na(2)-N(6)	105.44(13)
C(35A)-C(36A)	1.487(16)	N(5)-Na(2)-N(6)	55.65(12)
O(1)-Na(1)-N(2)	113.01(14)	O(2)-Na(2)-Na(3)	150.09(12)
O(1)-Na(1)-N(4)	116.36(14)	N(3)-Na(2)-Na(3)	54.64(10)
N(2)-Na(1)-N(4)	124.69(12)	N(2)-Na(2)-Na(3)	79.91(9)
O(1)-Na(1)-N(1)	126.01(15)	N(5)-Na(2)-Na(3)	51.23(10)
N(2)-Na(1)-N(1)	63.34(12)	N(6)-Na(2)-Na(3)	90.24(10)
N(4)-Na(1)-N(1)	103.41(13)	O(2)-Na(2)-S(1)	131.79(12)
O(1)-Na(1)-C(2A)	76.8(3)	N(3)-Na(2)-S(1)	29.57(8)
N(2)-Na(1)-C(2A)	82.7(3)	N(2)-Na(2)-S(1)	31.37(8)
N(4)-Na(1)-C(2A)	131.3(3)	N(5)-Na(2)-S(1)	110.81(10)
N(1)-Na(1)-C(2A)	49.3(2)	N(6)-Na(2)-S(1)	125.04(10)
O(1)-Na(1)-S(1)	126.77(12)	Na(3)-Na(2)-S(1)	59.75(4)
N(2)-Na(1)-S(1)	31.52(8)	O(2)-Na(2)-Na(1)	150.88(11)
N(4)-Na(1)-S(1)	116.50(10)	N(3)-Na(2)-Na(1)	81.30(9)

N(2)-Na(2)-Na(1)	44.52(8)	N(4)-Na(3)-Na(1)	47.96(9)
N(5)-Na(2)-Na(1)	82.10(9)	S(1)-Na(3)-Na(1)	59.22(4)
N(6)-Na(2)-Na(1)	67.86(10)	Na(2)-Na(3)-Na(1)	63.80(5)
Na(3)-Na(2)-Na(1)	58.23(5)	O(3)-Na(3)-S(2)	115.42(12)
S(1)-Na(2)-Na(1)	57.25(4)	N(1)-Na(3)-S(2)	119.40(10)
O(2)-Na(2)-S(2)	116.13(12)	N(5)-Na(3)-S(2)	27.51(8)
N(3)-Na(2)-S(2)	116.38(11)	N(3)-Na(3)-S(2)	111.03(10)
N(2)-Na(2)-S(2)	108.92(9)	N(4)-Na(3)-S(2)	28.51(8)
N(5)-Na(2)-S(2)	27.72(8)	S(1)-Na(3)-S(2)	112.80(5)
N(6)-Na(2)-S(2)	30.87(9)	Na(2)-Na(3)-S(2)	62.18(4)
Na(3)-Na(2)-S(2)	61.74(5)	Na(1)-Na(3)-S(2)	66.45(5)
S(1)-Na(2)-S(2)	112.07(5)	N(3)-S(1)-N(1)	102.93(19)
Na(1)-Na(2)-S(2)	64.41(4)	N(3)-S(1)-N(2)	99.09(18)
O(3)-Na(3)-N(1)	114.66(14)	N(1)-S(1)-N(2)	103.13(18)
O(3)-Na(3)-N(5)	110.60(14)	N(3)-S(1)-Na(1)	104.09(15)
N(1)-Na(3)-N(5)	134.39(14)	N(1)-S(1)-Na(1)	55.74(14)
O(3)-Na(3)-N(3)	125.42(14)	N(2)-S(1)-Na(1)	47.57(12)
N(1)-Na(3)-N(3)	60.03(12)	N(3)-S(1)-Na(3)	57.20(15)
N(5)-Na(3)-N(3)	97.60(13)	N(1)-S(1)-Na(3)	48.57(13)
O(3)-Na(3)-N(4)	114.81(14)	N(2)-S(1)-Na(3)	93.83(12)
N(1)-Na(3)-N(4)	98.60(12)	Na(1)-S(1)-Na(3)	60.69(5)
N(5)-Na(3)-N(4)	56.01(11)	N(3)-S(1)-Na(2)	49.38(14)
N(3)-Na(3)-N(4)	119.71(12)	N(1)-S(1)-Na(2)	101.27(14)
O(3)-Na(3)-S(1)	131.68(12)	N(2)-S(1)-Na(2)	50.92(12)
N(1)-Na(3)-S(1)	31.04(9)	Na(1)-S(1)-Na(2)	64.28(5)
N(5)-Na(3)-S(1)	113.36(11)	Na(3)-S(1)-Na(2)	60.06(4)
N(3)-Na(3)-S(1)	30.30(8)	C(1)-N(1)-S(1)	115.9(8)
N(4)-Na(3)-S(1)	105.86(9)	C(1A)-N(1)-S(1)	112.4(6)
O(3)-Na(3)-Na(2)	152.61(12)	C(1)-N(1)-Na(3)	139.0(8)
N(1)-Na(3)-Na(2)	86.33(10)	C(1A)-N(1)-Na(3)	144.1(6)
N(5)-Na(3)-Na(2)	53.35(10)	S(1)-N(1)-Na(3)	100.39(16)
N(3)-Na(3)-Na(2)	48.85(9)	C(1)-N(1)-Na(1)	115.9(8)
N(4)-Na(3)-Na(2)	76.59(9)	C(1A)-N(1)-Na(1)	112.6(6)
S(1)-Na(3)-Na(2)	60.19(4)	S(1)-N(1)-Na(1)	92.36(16)
O(3)-Na(3)-Na(1)	142.73(12)	Na(3)-N(1)-Na(1)	78.87(12)
N(1)-Na(3)-Na(1)	53.10(10)	N(1)-C(1)-C(2)	111.7(14)
N(5)-Na(3)-Na(1)	86.86(9)	N(1)-C(1)-C(4)	105.1(15)
N(3)-Na(3)-Na(1)	81.89(8)	C(2)-C(1)-C(4)	109.4(17)

N(1)-C(1)-C(3)	114.8(14)	N(6)-S(2)-Na(2)	53.25(14)
C(2)-C(1)-C(3)	105.5(14)	Na(3)-S(2)-Na(2)	56.07(4)
C(4)-C(1)-C(3)	110.4(15)	C(13)-N(4)-S(2)	112.9(3)
C(5)-N(2)-S(1)	112.5(3)	C(13)-N(4)-Na(1)	123.5(3)
C(5)-N(2)-Na(1)	138.0(2)	S(2)-N(4)-Na(1)	122.4(2)
S(1)-N(2)-Na(1)	100.91(16)	C(13)-N(4)-Na(3)	112.7(3)
C(5)-N(2)-Na(2)	111.2(2)	S(2)-N(4)-Na(3)	95.38(16)
S(1)-N(2)-Na(2)	97.70(15)	Na(1)-N(4)-Na(3)	73.99(11)
Na(1)-N(2)-Na(2)	87.70(12)	C(14)-C(13)-N(4)	113.5(4)
N(2)-C(5)-C(6)	115.3(3)	C(14)-C(13)-C(15)	108.0(6)
N(2)-C(5)-C(7)	103.0(3)	N(4)-C(13)-C(15)	103.0(4)
C(6)-C(5)-C(7)	106.8(4)	C(14)-C(13)-C(16)	105.7(6)
N(2)-C(5)-C(8)	113.1(4)	N(4)-C(13)-C(16)	114.8(4)
C(6)-C(5)-C(8)	106.0(4)	C(15)-C(13)-C(16)	111.7(5)
C(7)-C(5)-C(8)	112.6(4)	C(17)-N(5)-S(2)	112.6(4)
C(9A)-N(3)-S(1)	121.4(9)	C(17A)-N(5)-S(2)	101.1(11)
C(9)-N(3)-S(1)	109.4(4)	C(17)-N(5)-Na(3)	133.3(3)
C(9A)-N(3)-Na(2)	137.5(9)	C(17A)-N(5)-Na(3)	142.9(11)
C(9)-N(3)-Na(2)	147.1(4)	S(2)-N(5)-Na(3)	105.50(17)
S(1)-N(3)-Na(2)	101.05(16)	C(17)-N(5)-Na(2)	118.9(3)
C(9A)-N(3)-Na(3)	102.4(9)	C(17A)-N(5)-Na(2)	122.9(12)
C(9)-N(3)-Na(3)	113.3(4)	S(2)-N(5)-Na(2)	103.02(17)
S(1)-N(3)-Na(3)	92.50(16)	Na(3)-N(5)-Na(2)	75.43(13)
Na(2)-N(3)-Na(3)	76.50(13)	N(5)-C(17)-C(19)	112.8(5)
N(3)-C(9)-C(12)	101.5(5)	N(5)-C(17)-C(18)	100.9(5)
N(3)-C(9)-C(11)	108.3(6)	C(19)-C(17)-C(18)	111.4(5)
C(12)-C(9)-C(11)	109.5(7)	N(5)-C(17)-C(20)	113.3(5)
N(3)-C(9)-C(10)	121.0(5)	C(19)-C(17)-C(20)	107.1(6)
C(12)-C(9)-C(10)	107.4(6)	C(18)-C(17)-C(20)	111.5(6)
C(11)-C(9)-C(10)	108.7(6)	H(1)-N(6)-C(21)	95(5)
N(5)-S(2)-N(4)	103.1(2)	H(1)-N(6)-S(2)	114(4)
N(5)-S(2)-N(6)	96.1(2)	C(21)-N(6)-S(2)	118.3(3)
N(4)-S(2)-N(6)	102.4(2)	H(1)-N(6)-Na(2)	79(5)
N(5)-S(2)-Na(3)	46.99(14)	C(21)-N(6)-Na(2)	144.4(3)
N(4)-S(2)-Na(3)	56.12(14)	S(2)-N(6)-Na(2)	95.89(18)
N(6)-S(2)-Na(3)	105.67(15)	N(6)-C(21)-C(22)	105.8(4)
N(5)-S(2)-Na(2)	49.26(15)	N(6)-C(21)-C(23)	107.0(4)
N(4)-S(2)-Na(2)	88.82(14)	C(22)-C(21)-C(23)	108.5(4)

N(6)-C(21)-C(24)	116.2(4)
C(22)-C(21)-C(24)	110.6(5)
C(23)-C(21)-C(24)	108.5(4)
N(3)-C(9A)-C(10A)	104.6(15)
N(3)-C(9A)-C(12A)	105.7(15)
C(10A)-C(9A)-C(12A)	113.5(18)
N(3)-C(9A)-C(11A)	114.0(18)
C(10A)-C(9A)-C(11A)	108.9(19)
C(12A)-C(9A)-C(11A)	110.1(18)
N(1)-C(1A)-C(2A)	107.0(10)
N(1)-C(1A)-C(4A)	105.2(11)
C(2A)-C(1A)-C(4A)	107.0(12)
N(1)-C(1A)-C(3A)	119.9(10)
C(2A)-C(1A)-C(3A)	109.5(10)
C(4A)-C(1A)-C(3A)	107.5(11)
C(1A)-C(2A)-Na(1)	89.5(7)
C(19A)-C(17A)-N(5)	111(2)
C(19A)-C(17A)-C(18A)	110(2)
N(5)-C(17A)-C(18A)	101.3(19)
C(19A)-C(17A)-C(20A)	105(2)
N(5)-C(17A)-C(20A)	123(2)
C(18A)-C(17A)-C(20A)	106(2)
C(28A)-O(1)-C(25A)	110.5(13)
C(28)-O(1)-C(25)	107.3(7)
C(28A)-O(1)-Na(1)	125.8(9)
C(28)-O(1)-Na(1)	132.2(6)
C(25A)-O(1)-Na(1)	119.9(7)
C(25)-O(1)-Na(1)	118.6(4)
O(1)-C(25)-C(26)	97.3(8)
C(27)-C(26)-C(25)	102.7(9)
C(26)-C(27)-C(28)	109.1(8)
O(1)-C(28)-C(27)	101.4(9)
C(29A)-O(2)-C(32A)	113.7(12)
C(29A)-O(2)-Na(2)	121.3(8)
C(32A)-O(2)-Na(2)	124.2(10)
C(32)-O(2)-Na(2)	135.9(4)
C(29)-O(2)-Na(2)	115.1(4)
O(2)-C(29)-C(30)	105.4(6)
C(29)-C(30)-C(31)	102.7(7)
C(32)-C(31)-C(30)	103.5(7)
O(2)-C(32)-C(31)	106.8(7)
C(36A)-O(3)-C(33A)	114.8(9)
C(36)-O(3)-C(33)	105.5(7)
C(36A)-O(3)-Na(3)	124.5(8)
C(33A)-O(3)-Na(3)	120.4(5)
C(36)-O(3)-Na(3)	127.3(6)
C(33)-O(3)-Na(3)	125.7(4)
C(34)-C(33)-O(3)	107.9(8)
C(33)-C(34)-C(35)	105.3(9)
C(36)-C(35)-C(34)	104.9(9)
O(3)-C(36)-C(35)	102.7(8)
O(1)-C(25A)-C(26A)	97.0(11)
C(27A)-C(26A)-C(25A)	106.2(12)
C(26A)-C(27A)-C(28A)	108.1(13)
O(1)-C(28A)-C(27A)	97.7(12)
O(2)-C(29A)-C(30A)	103.3(13)
C(29A)-C(30A)-C(31A)	107.8(14)
C(32A)-C(31A)-C(30A)	103.6(16)
O(2)-C(32A)-C(31A)	102.3(16)
O(3)-C(33A)-C(34A)	106.4(10)
C(33A)-C(34A)-C(35A)	105.5(12)
C(34A)-C(35A)-C(36A)	107.4(12)
O(3)-C(36A)-C(35A)	105.3(11)

Crystal structure of **4**

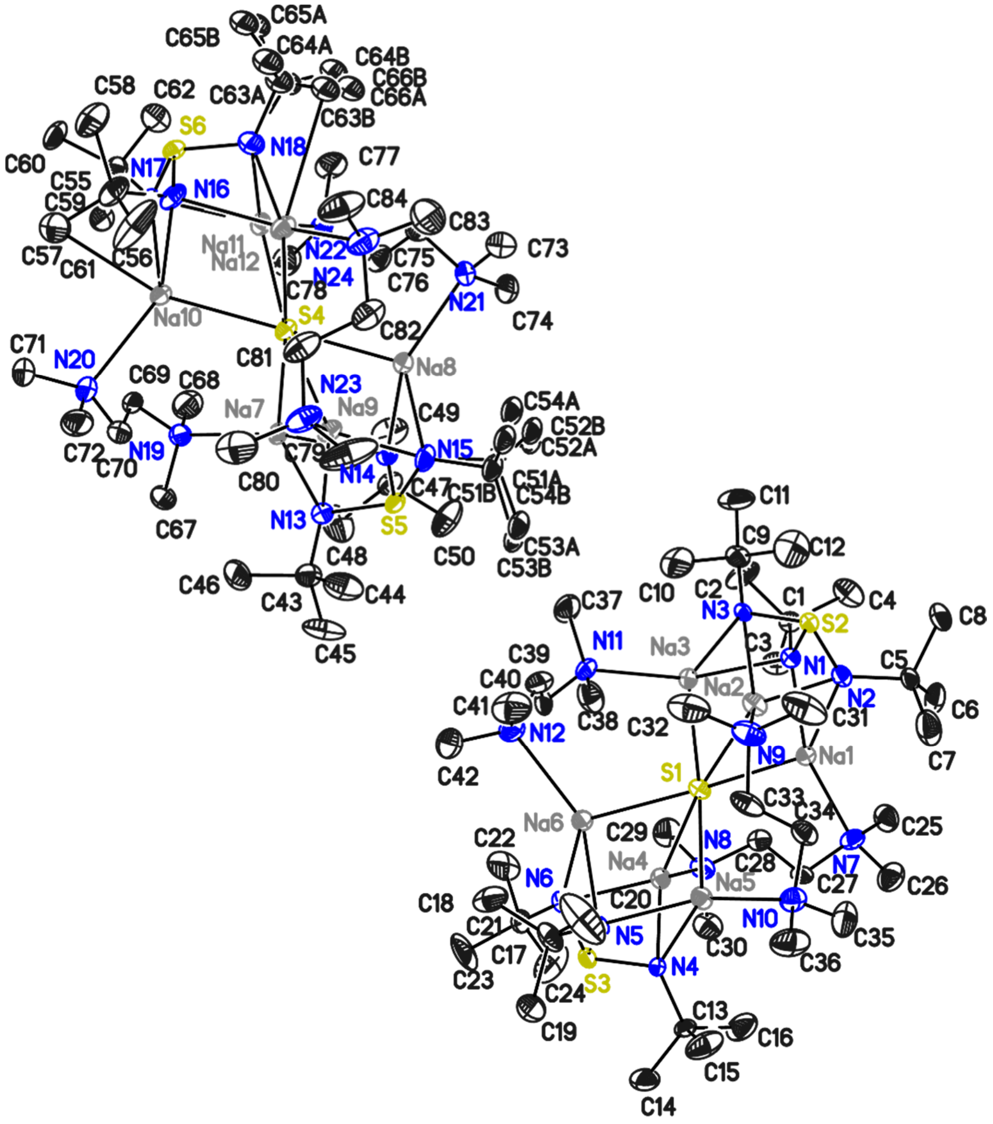

Figure S4. Asymmetric unit of **4** with thermal ellipsoids at 50% probability level.

The asymmetric unit display two complex molecules and two (N15-, N18-unit) of the twelve *t*Butyl-groups were disordered over two positions and restraints, on the anisotropic displacement parameters and on distances and angle distances, were applied. The occupancies of the minor positions refine to 0.389(8), and 0.147(4), respectively. The measured crystal was pseudo-merohedrally twinned with the twin law -1 0 0 0 -1 0 0 0 1, with the fractional contribution refined to 0.4166(10).

Table S6. Bond lengths [Å] and angles [°] for **4.**

S(1)-N(1)	1.645(3)	Na(4)-N(6)	2.468(3)
S(1)-N(2)	1.654(3)	Na(4)-N(8)	2.551(3)
S(1)-N(3)	1.655(3)	Na(4)-Na(5)	3.186(2)
S(1)-Na(3)	3.0330(17)	Na(4)-Na(6)	3.208(2)
S(1)-Na(1)	3.049(2)	Na(5)-N(5)	2.331(3)
S(1)-Na(2)	3.0513(18)	Na(5)-N(4)	2.484(3)
S(3)-N(5)	1.648(3)	Na(5)-N(10)	2.504(3)
S(3)-N(6)	1.653(3)	Na(5)-Na(6)	3.151(2)
S(3)-N(4)	1.658(3)	S(5)-Na(9)	2.7235(19)
S(3)-Na(5)	3.0316(18)	S(5)-Na(11)	2.7300(18)
S(3)-Na(6)	3.032(2)	S(5)-Na(7)	2.7323(18)
S(3)-Na(4)	3.0454(18)	S(5)-Na(12)	2.7393(19)
S(4)-N(15)	1.650(3)	S(5)-Na(10)	2.7528(17)
S(4)-N(13)	1.652(3)	S(5)-Na(8)	2.7623(17)
S(4)-N(14)	1.662(3)	Na(6)-N(6)	2.348(4)
S(4)-Na(9)	3.0316(18)	Na(6)-N(5)	2.477(4)
S(4)-Na(8)	3.033(2)	Na(6)-N(12)	2.537(4)
S(4)-Na(7)	3.0470(17)	Na(7)-N(13)	2.343(4)
Na(1)-N(1)	2.359(4)	Na(7)-N(14)	2.481(3)
Na(1)-N(2)	2.489(4)	Na(7)-N(19)	2.549(3)
Na(1)-N(7)	2.562(4)	Na(7)-Na(8)	3.187(2)
Na(1)-S(2)	2.7447(17)	Na(7)-Na(9)	3.189(2)
Na(1)-Na(3)	3.154(2)	Na(8)-N(14)	2.350(4)
Na(1)-Na(2)	3.228(2)	Na(8)-N(15)	2.462(4)
Na(2)-N(2)	2.342(3)	Na(8)-N(21)	2.523(4)
Na(2)-N(3)	2.464(4)	Na(8)-Na(9)	3.169(2)
Na(2)-N(9)	2.551(4)	Na(9)-N(15)	2.336(3)
Na(2)-S(2)	2.7470(18)	Na(9)-N(13)	2.459(4)
Na(2)-Na(3)	3.164(2)	Na(9)-N(23)	2.496(3)
S(2)-Na(3)	2.7232(18)	Na(10)-N(17)	2.349(3)
S(2)-Na(5)	2.7271(19)	Na(10)-N(16)	2.492(4)
S(2)-Na(4)	2.7375(18)	Na(10)-N(20)	2.556(4)
S(2)-Na(6)	2.7607(17)	Na(10)-S(6)	3.047(2)
Na(3)-N(3)	2.342(3)	Na(10)-C(57)	3.072(4)
Na(3)-N(1)	2.485(3)	Na(10)-Na(11)	3.165(2)
Na(3)-N(11)	2.492(3)	Na(10)-Na(12)	3.211(2)
Na(4)-N(4)	2.342(4)	Na(11)-N(18B)	2.31(3)

Na(11)-N(18A)	2.323(6)	N(16)-S(6)	1.657(3)
Na(11)-N(17)	2.483(3)	N(17)-C(59)	1.476(5)
Na(11)-N(22)	2.504(3)	N(17)-S(6)	1.663(3)
Na(11)-S(6)	3.0320(17)	N(19)-C(67)	1.466(5)
Na(11)-Na(12)	3.183(2)	N(19)-C(68)	1.470(6)
Na(12)-N(16)	2.345(3)	N(19)-C(69)	1.470(5)
Na(12)-N(18A)	2.451(9)	N(20)-C(71)	1.469(5)
Na(12)-N(18B)	2.50(5)	N(20)-C(72)	1.469(5)
Na(12)-N(24)	2.557(4)	N(20)-C(70)	1.476(5)
Na(12)-C(64B)	2.95(2)	N(21)-C(73)	1.453(5)
Na(12)-S(6)	3.0455(18)	N(21)-C(75)	1.473(5)
N(1)-C(1)	1.482(5)	N(21)-C(74)	1.478(5)
N(2)-C(5)	1.489(5)	N(22)-C(77)	1.471(5)
N(3)-C(9)	1.479(5)	N(22)-C(78)	1.474(5)
N(4)-C(13)	1.478(5)	N(22)-C(76)	1.475(5)
N(5)-C(17)	1.484(4)	N(23)-C(80)	1.462(6)
N(6)-C(21)	1.483(5)	N(23)-C(79)	1.468(5)
N(7)-C(26)	1.453(5)	N(23)-C(81)	1.476(6)
N(7)-C(25)	1.474(5)	N(24)-C(83)	1.463(6)
N(7)-C(27)	1.481(5)	N(24)-C(84)	1.472(5)
N(8)-C(29)	1.454(5)	N(24)-C(82)	1.483(6)
N(8)-C(28)	1.475(5)	C(1)-C(3)	1.512(5)
N(8)-C(30)	1.476(5)	C(1)-C(2)	1.531(5)
N(9)-C(32)	1.460(5)	C(1)-C(4)	1.533(5)
N(9)-C(33)	1.471(5)	C(5)-C(7)	1.522(6)
N(9)-C(31)	1.474(5)	C(5)-C(6)	1.526(6)
N(10)-C(35)	1.465(5)	C(5)-C(8)	1.544(6)
N(10)-C(36)	1.467(5)	C(9)-C(10)	1.507(5)
N(10)-C(34)	1.472(5)	C(9)-C(11)	1.527(6)
N(11)-C(38)	1.457(6)	C(9)-C(12)	1.541(5)
N(11)-C(37)	1.465(5)	C(13)-C(15)	1.518(5)
N(11)-C(39)	1.473(5)	C(13)-C(16)	1.527(5)
N(12)-C(41)	1.457(6)	C(13)-C(14)	1.527(6)
N(12)-C(42)	1.473(6)	C(17)-C(18)	1.518(5)
N(12)-C(40)	1.480(5)	C(17)-C(20)	1.522(5)
N(13)-C(43)	1.487(5)	C(17)-C(19)	1.550(5)
N(14)-C(47)	1.471(4)	C(21)-C(22)	1.510(6)
N(16)-C(55)	1.483(5)	C(21)-C(23)	1.532(5)

C(21)-C(24)	1.541(6)	N(1)-S(1)-N(3)	103.52(15)
C(27)-C(28)	1.506(5)	N(2)-S(1)-N(3)	102.04(16)
C(33)-C(34)	1.523(5)	N(1)-S(1)-Na(3)	54.96(11)
C(39)-C(40)	1.504(6)	N(2)-S(1)-Na(3)	100.59(12)
C(43)-C(46)	1.520(5)	N(3)-S(1)-Na(3)	50.01(11)
C(43)-C(45)	1.526(6)	N(1)-S(1)-Na(1)	50.07(12)
C(43)-C(44)	1.533(6)	N(2)-S(1)-Na(1)	54.64(12)
C(47)-C(48)	1.519(5)	N(3)-S(1)-Na(1)	101.69(13)
C(47)-C(50)	1.524(5)	Na(3)-S(1)-Na(1)	62.47(5)
C(47)-C(49)	1.532(5)	N(1)-S(1)-Na(2)	102.87(12)
N(15)-C(51B)	1.467(10)	N(2)-S(1)-Na(2)	49.47(11)
N(15)-C(51A)	1.496(7)	N(3)-S(1)-Na(2)	53.70(12)
C(51A)-C(53A)	1.536(8)	Na(3)-S(1)-Na(2)	62.67(4)
C(51A)-C(52A)	1.538(8)	Na(1)-S(1)-Na(2)	63.90(5)
C(51A)-C(54A)	1.549(8)	N(5)-S(3)-N(6)	103.82(17)
C(51B)-C(52B)	1.535(11)	N(5)-S(3)-N(4)	103.15(15)
C(51B)-C(54B)	1.542(11)	N(6)-S(3)-N(4)	102.43(16)
C(51B)-C(53B)	1.555(11)	N(5)-S(3)-Na(5)	49.62(10)
C(55)-C(58)	1.517(6)	N(6)-S(3)-Na(5)	101.21(12)
C(55)-C(57)	1.528(6)	N(4)-S(3)-Na(5)	54.97(12)
C(55)-C(56)	1.531(5)	N(5)-S(3)-Na(6)	54.69(12)
C(59)-C(61)	1.536(5)	N(6)-S(3)-Na(6)	50.22(12)
C(59)-C(60)	1.543(5)	N(4)-S(3)-Na(6)	102.05(13)
C(59)-C(62)	1.548(5)	Na(5)-S(3)-Na(6)	62.61(5)
S(6)-N(18B)	1.649(15)	N(5)-S(3)-Na(4)	102.46(12)
S(6)-N(18A)	1.653(4)	N(6)-S(3)-Na(4)	54.01(11)
N(18A)-C(63A)	1.477(6)	N(4)-S(3)-Na(4)	49.64(12)
C(63A)-C(66A)	1.515(6)	Na(5)-S(3)-Na(4)	63.23(4)
C(63A)-C(65A)	1.537(7)	Na(6)-S(3)-Na(4)	63.72(5)
C(63A)-C(64A)	1.540(6)	N(15)-S(4)-N(13)	102.76(16)
N(18B)-C(63B)	1.472(14)	N(15)-S(4)-N(14)	103.23(18)
C(63B)-C(64B)	1.535(14)	N(13)-S(4)-N(14)	102.82(16)
C(63B)-C(65B)	1.535(14)	N(15)-S(4)-Na(9)	49.80(11)
C(63B)-C(66B)	1.546(13)	N(13)-S(4)-Na(9)	54.11(12)
C(69)-C(70)	1.508(5)	N(14)-S(4)-Na(9)	102.07(12)
C(75)-C(76)	1.510(6)	N(15)-S(4)-Na(8)	54.18(13)
C(81)-C(82)	1.498(6)	N(13)-S(4)-Na(8)	101.33(14)
N(1)-S(1)-N(2)	103.54(17)	N(14)-S(4)-Na(8)	50.31(12)

Na(9)-S(4)-Na(8)	63.00(5)
N(15)-S(4)-Na(7)	101.83(12)
N(13)-S(4)-Na(7)	49.59(12)
N(14)-S(4)-Na(7)	54.42(11)
Na(9)-S(4)-Na(7)	63.29(4)
Na(8)-S(4)-Na(7)	63.22(5)
N(1)-Na(1)-N(2)	64.56(11)
N(1)-Na(1)-N(7)	126.60(12)
N(2)-Na(1)-N(7)	137.17(12)
N(1)-Na(1)-S(2)	105.33(9)
N(2)-Na(1)-S(2)	100.07(9)
N(7)-Na(1)-S(2)	112.82(10)
N(1)-Na(1)-S(1)	32.33(8)
N(2)-Na(1)-S(1)	32.82(8)
N(7)-Na(1)-S(1)	146.28(10)
S(2)-Na(1)-S(1)	100.48(6)
N(1)-Na(1)-Na(3)	51.14(8)
N(2)-Na(1)-Na(3)	81.34(9)
N(7)-Na(1)-Na(3)	140.20(9)
S(2)-Na(1)-Na(3)	54.45(4)
S(1)-Na(1)-Na(3)	58.52(5)
N(1)-Na(1)-Na(2)	83.47(9)
N(2)-Na(1)-Na(2)	46.17(8)
N(7)-Na(1)-Na(2)	149.63(10)
S(2)-Na(1)-Na(2)	54.03(4)
S(1)-Na(1)-Na(2)	58.09(4)
Na(3)-Na(1)-Na(2)	59.43(5)
N(2)-Na(2)-N(3)	64.65(11)
N(2)-Na(2)-N(9)	127.57(12)
N(3)-Na(2)-N(9)	137.12(13)
N(2)-Na(2)-S(2)	103.87(10)
N(3)-Na(2)-S(2)	101.29(9)
N(9)-Na(2)-S(2)	112.07(10)
N(2)-Na(2)-S(1)	32.46(8)
N(3)-Na(2)-S(1)	32.77(7)
N(9)-Na(2)-S(1)	147.05(10)
S(2)-Na(2)-S(1)	100.37(5)
N(2)-Na(2)-Na(3)	83.34(9)
N(3)-Na(2)-Na(3)	47.19(8)
N(9)-Na(2)-Na(3)	148.94(10)
S(2)-Na(2)-Na(3)	54.31(4)
S(1)-Na(2)-Na(3)	58.38(4)
N(2)-Na(2)-Na(1)	50.04(9)
N(3)-Na(2)-Na(1)	81.27(8)
N(9)-Na(2)-Na(1)	140.38(11)
S(2)-Na(2)-Na(1)	53.97(4)
S(1)-Na(2)-Na(1)	58.01(4)
Na(3)-Na(2)-Na(1)	59.12(5)
Na(3)-S(2)-Na(5)	171.58(5)
Na(3)-S(2)-Na(4)	113.74(6)
Na(5)-S(2)-Na(4)	71.32(5)
Na(3)-S(2)-Na(1)	70.45(5)
Na(5)-S(2)-Na(1)	115.69(6)
Na(4)-S(2)-Na(1)	103.41(5)
Na(3)-S(2)-Na(2)	70.68(5)
Na(5)-S(2)-Na(2)	105.07(6)
Na(4)-S(2)-Na(2)	172.58(6)
Na(1)-S(2)-Na(2)	72.00(5)
Na(3)-S(2)-Na(6)	104.59(6)
Na(5)-S(2)-Na(6)	70.08(5)
Na(4)-S(2)-Na(6)	71.40(5)
Na(1)-S(2)-Na(6)	171.09(6)
Na(2)-S(2)-Na(6)	113.90(5)
N(3)-Na(3)-N(1)	64.86(11)
N(3)-Na(3)-N(11)	129.75(13)
N(1)-Na(3)-N(11)	137.93(12)
N(3)-Na(3)-S(2)	105.29(10)
N(1)-Na(3)-S(2)	102.50(9)
N(11)-Na(3)-S(2)	108.29(10)
N(3)-Na(3)-S(1)	32.77(8)
N(1)-Na(3)-S(1)	32.81(7)
N(11)-Na(3)-S(1)	150.00(11)
S(2)-Na(3)-S(1)	101.38(5)
N(3)-Na(3)-Na(1)	84.73(9)
N(1)-Na(3)-Na(1)	47.65(8)
N(11)-Na(3)-Na(1)	145.46(11)

S(2)-Na(3)-Na(1)	55.09(4)
S(1)-Na(3)-Na(1)	59.01(4)
N(3)-Na(3)-Na(2)	50.50(9)
N(1)-Na(3)-Na(2)	82.93(8)
N(11)-Na(3)-Na(2)	138.55(10)
S(2)-Na(3)-Na(2)	55.01(5)
S(1)-Na(3)-Na(2)	58.95(4)
Na(1)-Na(3)-Na(2)	61.45(4)
N(4)-Na(4)-N(6)	64.86(11)
N(4)-Na(4)-N(8)	127.38(13)
N(6)-Na(4)-N(8)	136.29(12)
N(4)-Na(4)-S(2)	104.68(9)
N(6)-Na(4)-S(2)	101.14(9)
N(8)-Na(4)-S(2)	112.42(10)
N(4)-Na(4)-S(3)	32.66(8)
N(6)-Na(4)-S(3)	32.83(8)
N(8)-Na(4)-S(3)	146.66(10)
S(2)-Na(4)-S(3)	100.58(5)
N(4)-Na(4)-Na(5)	50.63(8)
N(6)-Na(4)-Na(5)	81.34(8)
N(8)-Na(4)-Na(5)	141.22(9)
S(2)-Na(4)-Na(5)	54.19(4)
S(3)-Na(4)-Na(5)	58.17(4)
N(4)-Na(4)-Na(6)	83.38(9)
N(6)-Na(4)-Na(6)	46.65(8)
N(8)-Na(4)-Na(6)	148.92(10)
S(2)-Na(4)-Na(6)	54.64(4)
S(3)-Na(4)-Na(6)	57.94(4)
Na(5)-Na(4)-Na(6)	59.05(5)
N(5)-Na(5)-N(4)	64.99(11)
N(5)-Na(5)-N(10)	129.01(12)
N(4)-Na(5)-N(10)	137.76(13)
N(5)-Na(5)-S(2)	106.26(10)
N(4)-Na(5)-S(2)	101.16(9)
N(10)-Na(5)-S(2)	109.26(10)
N(5)-Na(5)-S(3)	32.58(7)
N(4)-Na(5)-S(3)	33.14(7)
N(10)-Na(5)-S(3)	149.31(11)
S(2)-Na(5)-S(3)	101.17(5)
N(5)-Na(5)-Na(6)	51.09(9)
N(4)-Na(5)-Na(6)	82.47(9)
N(10)-Na(5)-Na(6)	139.16(11)
S(2)-Na(5)-Na(6)	55.46(5)
S(3)-Na(5)-Na(6)	58.71(4)
N(5)-Na(5)-Na(4)	84.48(8)
N(4)-Na(5)-Na(4)	46.79(8)
N(10)-Na(5)-Na(4)	146.38(10)
S(2)-Na(5)-Na(4)	54.49(4)
S(3)-Na(5)-Na(4)	58.60(4)
Na(6)-Na(5)-Na(4)	60.83(4)
Na(9)-S(5)-Na(11)	171.38(5)
Na(9)-S(5)-Na(7)	71.54(5)
Na(11)-S(5)-Na(7)	113.17(6)
Na(9)-S(5)-Na(12)	104.97(6)
Na(11)-S(5)-Na(12)	71.17(5)
Na(7)-S(5)-Na(12)	172.68(6)
Na(9)-S(5)-Na(10)	116.09(6)
Na(11)-S(5)-Na(10)	70.52(5)
Na(7)-S(5)-Na(10)	103.88(5)
Na(12)-S(5)-Na(10)	71.56(5)
Na(9)-S(5)-Na(8)	70.56(5)
Na(11)-S(5)-Na(8)	103.65(6)
Na(7)-S(5)-Na(8)	70.90(5)
Na(12)-S(5)-Na(8)	114.44(6)
Na(10)-S(5)-Na(8)	170.36(6)
N(6)-Na(6)-N(5)	65.09(11)
N(6)-Na(6)-N(12)	125.04(13)
N(5)-Na(6)-N(12)	136.52(12)
N(6)-Na(6)-S(2)	103.65(9)
N(5)-Na(6)-S(2)	101.27(9)
N(12)-Na(6)-S(2)	114.07(10)
N(6)-Na(6)-S(3)	32.76(8)
N(5)-Na(6)-S(3)	32.87(7)
N(12)-Na(6)-S(3)	144.77(11)
S(2)-Na(6)-S(3)	100.37(6)
N(6)-Na(6)-Na(5)	83.91(9)

N(5)-Na(6)-Na(5)	47.08(8)
N(12)-Na(6)-Na(5)	150.79(11)
S(2)-Na(6)-Na(5)	54.46(4)
S(3)-Na(6)-Na(5)	58.68(5)
N(6)-Na(6)-Na(4)	49.85(8)
N(5)-Na(6)-Na(4)	81.77(8)
N(12)-Na(6)-Na(4)	139.56(11)
S(2)-Na(6)-Na(4)	53.97(4)
S(3)-Na(6)-Na(4)	58.34(5)
Na(5)-Na(6)-Na(4)	60.12(5)
N(13)-Na(7)-N(14)	64.90(11)
N(13)-Na(7)-N(19)	128.81(13)
N(14)-Na(7)-N(19)	135.46(12)
N(13)-Na(7)-S(5)	103.94(10)
N(14)-Na(7)-S(5)	101.77(9)
N(19)-Na(7)-S(5)	112.02(10)
N(13)-Na(7)-S(4)	32.48(8)
N(14)-Na(7)-S(4)	33.03(7)
N(19)-Na(7)-S(4)	147.14(10)
S(5)-Na(7)-S(4)	100.59(5)
N(13)-Na(7)-Na(8)	83.27(9)
N(14)-Na(7)-Na(8)	46.97(8)
N(19)-Na(7)-Na(8)	147.54(10)
S(5)-Na(7)-Na(8)	54.99(4)
S(4)-Na(7)-Na(8)	58.17(4)
N(13)-Na(7)-Na(9)	49.97(9)
N(14)-Na(7)-Na(9)	81.78(8)
N(19)-Na(7)-Na(9)	141.81(9)
S(5)-Na(7)-Na(9)	54.10(4)
S(4)-Na(7)-Na(9)	58.12(4)
Na(8)-Na(7)-Na(9)	59.60(4)
N(14)-Na(8)-N(15)	65.26(11)
N(14)-Na(8)-N(21)	126.05(12)
N(15)-Na(8)-N(21)	135.62(12)
N(14)-Na(8)-S(5)	104.42(9)
N(15)-Na(8)-S(5)	100.91(9)
N(21)-Na(8)-S(5)	113.91(10)
N(14)-Na(8)-S(4)	32.98(8)
N(15)-Na(8)-S(4)	32.91(8)
N(21)-Na(8)-S(4)	145.44(10)
S(5)-Na(8)-S(4)	100.25(6)
N(14)-Na(8)-Na(9)	84.22(9)
N(15)-Na(8)-Na(9)	46.97(8)
N(21)-Na(8)-Na(9)	149.31(11)
S(5)-Na(8)-Na(9)	54.15(4)
S(4)-Na(8)-Na(9)	58.48(5)
N(14)-Na(8)-Na(7)	50.51(8)
N(15)-Na(8)-Na(7)	82.13(9)
N(21)-Na(8)-Na(7)	140.64(10)
S(5)-Na(8)-Na(7)	54.11(4)
S(4)-Na(8)-Na(7)	58.61(5)
Na(9)-Na(8)-Na(7)	60.24(5)
N(15)-Na(9)-N(13)	65.04(11)
N(15)-Na(9)-N(23)	128.56(12)
N(13)-Na(9)-N(23)	136.91(14)
N(15)-Na(9)-S(5)	105.48(10)
N(13)-Na(9)-S(5)	101.06(9)
N(23)-Na(9)-S(5)	110.66(10)
N(15)-Na(9)-S(4)	32.65(8)
N(13)-Na(9)-S(4)	32.97(8)
N(23)-Na(9)-S(4)	147.92(10)
S(5)-Na(9)-S(4)	101.18(5)
N(15)-Na(9)-Na(8)	50.41(9)
N(13)-Na(9)-Na(8)	81.91(9)
N(23)-Na(9)-Na(8)	140.34(11)
S(5)-Na(9)-Na(8)	55.29(4)
S(4)-Na(9)-Na(8)	58.52(4)
N(15)-Na(9)-Na(7)	84.00(9)
N(13)-Na(9)-Na(7)	46.84(8)
N(23)-Na(9)-Na(7)	147.23(10)
S(5)-Na(9)-Na(7)	54.36(4)
S(4)-Na(9)-Na(7)	58.59(4)
Na(8)-Na(9)-Na(7)	60.17(4)
N(17)-Na(10)-N(16)	65.03(11)
N(17)-Na(10)-N(20)	125.99(12)
N(16)-Na(10)-N(20)	138.54(12)

N(17)-Na(10)-S(5)	105.12(9)
N(16)-Na(10)-S(5)	100.33(9)
N(20)-Na(10)-S(5)	111.89(10)
N(17)-Na(10)-S(6)	32.77(8)
N(16)-Na(10)-S(6)	32.93(8)
N(20)-Na(10)-S(6)	147.20(10)
S(5)-Na(10)-S(6)	100.21(6)
N(17)-Na(10)-C(57)	79.62(13)
N(16)-Na(10)-C(57)	52.24(12)
N(20)-Na(10)-C(57)	88.22(12)
S(5)-Na(10)-C(57)	148.04(11)
S(6)-Na(10)-C(57)	66.42(10)
N(17)-Na(10)-Na(11)	50.95(8)
N(16)-Na(10)-Na(11)	81.69(9)
N(20)-Na(10)-Na(11)	138.41(10)
S(5)-Na(10)-Na(11)	54.40(4)
S(6)-Na(10)-Na(11)	58.40(5)
C(57)-Na(10)-Na(11)	124.53(11)
N(17)-Na(10)-Na(12)	84.04(9)
N(16)-Na(10)-Na(12)	46.46(8)
N(20)-Na(10)-Na(12)	149.88(10)
S(5)-Na(10)-Na(12)	54.02(4)
S(6)-Na(10)-Na(12)	58.17(5)
C(57)-Na(10)-Na(12)	96.12(10)
Na(11)-Na(10)-Na(12)	59.88(5)
N(18B)-Na(11)-N(17)	65.0(4)
N(18A)-Na(11)-N(17)	65.31(13)
N(18B)-Na(11)-N(22)	128.2(7)
N(18A)-Na(11)-N(22)	128.90(18)
N(17)-Na(11)-N(22)	138.01(12)
N(18B)-Na(11)-S(5)	105.7(12)
N(18A)-Na(11)-S(5)	104.3(2)
N(17)-Na(11)-S(5)	102.12(9)
N(22)-Na(11)-S(5)	109.35(10)
N(18B)-Na(11)-S(6)	32.5(4)
N(18A)-Na(11)-S(6)	32.67(10)
N(17)-Na(11)-S(6)	33.24(7)
N(22)-Na(11)-S(6)	149.03(11)
S(5)-Na(11)-S(6)	101.10(5)
N(18B)-Na(11)-Na(10)	84.8(7)
N(18A)-Na(11)-Na(10)	84.10(15)
N(17)-Na(11)-Na(10)	47.27(8)
N(22)-Na(11)-Na(10)	146.96(11)
S(5)-Na(11)-Na(10)	55.08(4)
S(6)-Na(11)-Na(10)	58.85(4)
N(18B)-Na(11)-Na(12)	51.3(12)
N(18A)-Na(11)-Na(12)	49.9(2)
N(17)-Na(11)-Na(12)	82.61(8)
N(22)-Na(11)-Na(12)	138.60(10)
S(5)-Na(11)-Na(12)	54.55(5)
S(6)-Na(11)-Na(12)	58.63(4)
Na(10)-Na(11)-Na(12)	60.78(5)
N(16)-Na(12)-N(18A)	64.88(13)
N(16)-Na(12)-N(18B)	64.9(4)
N(16)-Na(12)-N(24)	126.60(13)
N(18A)-Na(12)-N(24)	137.68(17)
N(18B)-Na(12)-N(24)	138.0(6)
N(16)-Na(12)-S(5)	104.63(10)
N(18A)-Na(12)-S(5)	100.61(15)
N(18B)-Na(12)-S(5)	100.2(7)
N(24)-Na(12)-S(5)	112.27(10)
N(16)-Na(12)-C(64B)	92.7(4)
N(18B)-Na(12)-C(64B)	53.2(5)
N(24)-Na(12)-C(64B)	84.9(4)
S(5)-Na(12)-C(64B)	138.1(5)
N(16)-Na(12)-S(6)	32.64(8)
N(18A)-Na(12)-S(6)	32.78(10)
N(18B)-Na(12)-S(6)	32.8(3)
N(24)-Na(12)-S(6)	146.63(10)
S(5)-Na(12)-S(6)	100.54(5)
C(64B)-Na(12)-S(6)	74.2(4)
N(16)-Na(12)-Na(11)	83.54(9)
N(18A)-Na(12)-Na(11)	46.49(15)
N(18B)-Na(12)-Na(11)	46.1(7)
N(24)-Na(12)-Na(11)	149.70(10)
S(5)-Na(12)-Na(11)	54.28(4)

C(64B)-Na(12)-Na(11)	91.3(4)	S(3)-N(5)-Na(6)	92.43(14)
S(6)-Na(12)-Na(11)	58.21(4)	Na(5)-N(5)-Na(6)	81.83(11)
N(16)-Na(12)-Na(10)	50.39(9)	C(21)-N(6)-S(3)	115.0(3)
N(18A)-Na(12)-Na(10)	81.17(12)	C(21)-N(6)-Na(6)	143.2(3)
N(18B)-Na(12)-Na(10)	80.9(5)	S(3)-N(6)-Na(6)	97.02(14)
N(24)-Na(12)-Na(10)	139.80(11)	C(21)-N(6)-Na(4)	110.8(2)
S(5)-Na(12)-Na(10)	54.41(4)	S(3)-N(6)-Na(4)	93.16(13)
C(64B)-Na(12)-Na(10)	131.9(4)	Na(6)-N(6)-Na(4)	83.50(11)
S(6)-Na(12)-Na(10)	58.20(4)	C(26)-N(7)-C(25)	109.1(3)
Na(11)-Na(12)-Na(10)	59.34(5)	C(26)-N(7)-C(27)	108.7(3)
C(1)-N(1)-S(1)	115.8(3)	C(25)-N(7)-C(27)	110.8(3)
C(1)-N(1)-Na(1)	142.6(2)	C(26)-N(7)-Na(1)	109.3(2)
S(1)-N(1)-Na(1)	97.60(15)	C(25)-N(7)-Na(1)	113.9(3)
C(1)-N(1)-Na(3)	112.1(2)	C(27)-N(7)-Na(1)	104.9(2)
S(1)-N(1)-Na(3)	92.23(13)	C(29)-N(8)-C(28)	108.4(3)
Na(1)-N(1)-Na(3)	81.21(11)	C(29)-N(8)-C(30)	109.5(3)
C(5)-N(2)-S(1)	116.3(3)	C(28)-N(8)-C(30)	111.1(3)
C(5)-N(2)-Na(2)	143.4(2)	C(29)-N(8)-Na(4)	112.7(2)
S(1)-N(2)-Na(2)	98.08(14)	C(28)-N(8)-Na(4)	106.7(2)
C(5)-N(2)-Na(1)	105.8(2)	C(30)-N(8)-Na(4)	108.4(2)
S(1)-N(2)-Na(1)	92.54(13)	C(32)-N(9)-C(33)	109.6(4)
Na(2)-N(2)-Na(1)	83.79(11)	C(32)-N(9)-C(31)	108.6(4)
C(9)-N(3)-S(1)	116.0(3)	C(33)-N(9)-C(31)	111.4(4)
C(9)-N(3)-Na(3)	142.9(2)	C(32)-N(9)-Na(2)	110.3(3)
S(1)-N(3)-Na(3)	97.22(14)	C(33)-N(9)-Na(2)	104.4(2)
C(9)-N(3)-Na(2)	110.0(2)	C(31)-N(9)-Na(2)	112.5(3)
S(1)-N(3)-Na(2)	93.53(15)	C(35)-N(10)-C(36)	110.5(4)
Na(3)-N(3)-Na(2)	82.31(12)	C(35)-N(10)-C(34)	108.3(3)
C(13)-N(4)-S(3)	115.9(2)	C(36)-N(10)-C(34)	110.8(3)
C(13)-N(4)-Na(4)	144.2(2)	C(35)-N(10)-Na(5)	102.0(2)
S(3)-N(4)-Na(4)	97.70(15)	C(36)-N(10)-Na(5)	117.6(3)
C(13)-N(4)-Na(5)	106.9(2)	C(34)-N(10)-Na(5)	107.1(2)
S(3)-N(4)-Na(5)	91.89(14)	C(38)-N(11)-C(37)	109.2(4)
Na(4)-N(4)-Na(5)	82.57(12)	C(38)-N(11)-C(39)	108.8(4)
C(17)-N(5)-S(3)	117.1(2)	C(37)-N(11)-C(39)	111.0(3)
C(17)-N(5)-Na(5)	142.1(2)	C(38)-N(11)-Na(3)	104.1(2)
S(3)-N(5)-Na(5)	97.80(13)	C(37)-N(11)-Na(3)	113.4(3)
C(17)-N(5)-Na(6)	109.2(2)	C(39)-N(11)-Na(3)	110.0(2)

C(41)-N(12)-C(42)	110.4(4)	C(72)-N(20)-C(70)	108.1(3)
C(41)-N(12)-C(40)	109.0(4)	C(71)-N(20)-Na(10)	113.9(3)
C(42)-N(12)-C(40)	110.9(4)	C(72)-N(20)-Na(10)	109.8(3)
C(41)-N(12)-Na(6)	111.3(3)	C(70)-N(20)-Na(10)	105.9(2)
C(42)-N(12)-Na(6)	111.4(3)	C(73)-N(21)-C(75)	109.1(4)
C(40)-N(12)-Na(6)	103.6(2)	C(73)-N(21)-C(74)	108.9(3)
C(43)-N(13)-S(4)	115.5(3)	C(75)-N(21)-C(74)	110.7(4)
C(43)-N(13)-Na(7)	142.9(3)	C(73)-N(21)-Na(8)	109.1(3)
S(4)-N(13)-Na(7)	97.93(16)	C(75)-N(21)-Na(8)	103.2(2)
C(43)-N(13)-Na(9)	109.1(2)	C(74)-N(21)-Na(8)	115.6(3)
S(4)-N(13)-Na(9)	92.92(15)	C(77)-N(22)-C(78)	109.3(4)
Na(7)-N(13)-Na(9)	83.19(12)	C(77)-N(22)-C(76)	111.8(3)
C(47)-N(14)-S(4)	115.6(3)	C(78)-N(22)-C(76)	108.2(3)
C(47)-N(14)-Na(8)	144.4(2)	C(77)-N(22)-Na(11)	111.3(3)
S(4)-N(14)-Na(8)	96.71(14)	C(78)-N(22)-Na(11)	106.5(2)
C(47)-N(14)-Na(7)	109.0(2)	C(76)-N(22)-Na(11)	109.6(2)
S(4)-N(14)-Na(7)	92.55(13)	C(80)-N(23)-C(79)	108.7(4)
Na(8)-N(14)-Na(7)	82.51(11)	C(80)-N(23)-C(81)	110.2(3)
C(55)-N(16)-S(6)	116.8(3)	C(79)-N(23)-C(81)	110.6(4)
C(55)-N(16)-Na(12)	142.9(2)	C(80)-N(23)-Na(9)	103.6(3)
S(6)-N(16)-Na(12)	97.62(15)	C(79)-N(23)-Na(9)	116.8(3)
C(55)-N(16)-Na(10)	107.4(3)	C(81)-N(23)-Na(9)	106.7(2)
S(6)-N(16)-Na(10)	92.26(14)	C(83)-N(24)-C(84)	109.1(4)
Na(12)-N(16)-Na(10)	83.14(11)	C(83)-N(24)-C(82)	108.2(4)
C(59)-N(17)-S(6)	114.7(3)	C(84)-N(24)-C(82)	110.6(4)
C(59)-N(17)-Na(10)	143.7(2)	C(83)-N(24)-Na(12)	109.4(3)
S(6)-N(17)-Na(10)	97.35(14)	C(84)-N(24)-Na(12)	113.8(3)
C(59)-N(17)-Na(11)	112.3(2)	C(82)-N(24)-Na(12)	105.7(3)
S(6)-N(17)-Na(11)	91.81(13)	N(1)-C(1)-C(3)	105.6(3)
Na(10)-N(17)-Na(11)	81.79(11)	N(1)-C(1)-C(2)	112.7(3)
C(67)-N(19)-C(68)	107.6(4)	C(3)-C(1)-C(2)	108.1(3)
C(67)-N(19)-C(69)	111.9(3)	N(1)-C(1)-C(4)	112.8(3)
C(68)-N(19)-C(69)	108.7(3)	C(3)-C(1)-C(4)	108.8(3)
C(67)-N(19)-Na(7)	109.4(3)	C(2)-C(1)-C(4)	108.7(4)
C(68)-N(19)-Na(7)	112.7(2)	N(2)-C(5)-C(7)	104.4(3)
C(69)-N(19)-Na(7)	106.6(2)	N(2)-C(5)-C(6)	114.6(3)
C(71)-N(20)-C(72)	108.1(3)	C(7)-C(5)-C(6)	108.2(4)
C(71)-N(20)-C(70)	110.9(3)	N(2)-C(5)-C(8)	112.3(3)

C(7)-C(5)-C(8)	109.6(4)	N(14)-C(47)-C(48)	114.1(3)
C(6)-C(5)-C(8)	107.6(3)	N(14)-C(47)-C(50)	113.5(3)
N(3)-C(9)-C(10)	105.5(3)	C(48)-C(47)-C(50)	108.0(4)
N(3)-C(9)-C(11)	112.0(3)	N(14)-C(47)-C(49)	105.5(3)
C(10)-C(9)-C(11)	109.3(4)	C(48)-C(47)-C(49)	108.2(4)
N(3)-C(9)-C(12)	113.1(3)	C(50)-C(47)-C(49)	107.2(3)
C(10)-C(9)-C(12)	109.2(3)	C(51B)-N(15)-S(4)	116.5(5)
C(11)-C(9)-C(12)	107.7(4)	C(51A)-N(15)-S(4)	116.1(4)
N(4)-C(13)-C(15)	114.7(3)	C(51B)-N(15)-Na(9)	139.8(5)
N(4)-C(13)-C(16)	105.4(3)	C(51A)-N(15)-Na(9)	144.8(3)
C(15)-C(13)-C(16)	108.6(4)	S(4)-N(15)-Na(9)	97.55(14)
N(4)-C(13)-C(14)	113.3(3)	C(51B)-N(15)-Na(8)	114.4(5)
C(15)-C(13)-C(14)	106.5(4)	C(51A)-N(15)-Na(8)	104.7(3)
C(16)-C(13)-C(14)	108.0(4)	S(4)-N(15)-Na(8)	92.91(14)
N(5)-C(17)-C(18)	113.2(3)	Na(9)-N(15)-Na(8)	82.61(12)
N(5)-C(17)-C(20)	104.4(3)	N(15)-C(51A)-C(53A)	110.7(6)
C(18)-C(17)-C(20)	111.2(4)	N(15)-C(51A)-C(52A)	116.1(5)
N(5)-C(17)-C(19)	113.2(3)	C(53A)-C(51A)-C(52A)	110.0(6)
C(18)-C(17)-C(19)	107.4(3)	N(15)-C(51A)-C(54A)	104.5(5)
C(20)-C(17)-C(19)	107.4(4)	C(53A)-C(51A)-C(54A)	106.6(5)
N(6)-C(21)-C(22)	106.1(3)	C(52A)-C(51A)-C(54A)	108.3(5)
N(6)-C(21)-C(23)	112.6(4)	N(15)-C(51B)-C(52B)	109.9(8)
C(22)-C(21)-C(23)	108.3(4)	N(15)-C(51B)-C(54B)	106.3(8)
N(6)-C(21)-C(24)	112.9(3)	C(52B)-C(51B)-C(54B)	108.0(8)
C(22)-C(21)-C(24)	108.4(4)	N(15)-C(51B)-C(53B)	114.7(9)
C(23)-C(21)-C(24)	108.4(4)	C(52B)-C(51B)-C(53B)	107.9(9)
N(7)-C(27)-C(28)	113.5(3)	C(54B)-C(51B)-C(53B)	109.9(8)
N(8)-C(28)-C(27)	113.0(3)	N(16)-C(55)-C(58)	114.1(4)
N(9)-C(33)-C(34)	112.8(4)	N(16)-C(55)-C(57)	112.5(3)
N(10)-C(34)-C(33)	112.0(3)	C(58)-C(55)-C(57)	108.5(4)
N(11)-C(39)-C(40)	113.8(4)	N(16)-C(55)-C(56)	104.3(3)
N(12)-C(40)-C(39)	112.2(4)	C(58)-C(55)-C(56)	108.1(4)
N(13)-C(43)-C(46)	105.2(3)	C(57)-C(55)-C(56)	109.1(4)
N(13)-C(43)-C(45)	113.0(3)	C(55)-C(57)-Na(10)	83.0(2)
C(46)-C(43)-C(45)	108.9(4)	N(17)-C(59)-C(61)	105.4(3)
N(13)-C(43)-C(44)	113.2(4)	N(17)-C(59)-C(60)	112.5(3)
C(46)-C(43)-C(44)	108.6(4)	C(61)-C(59)-C(60)	108.2(3)
C(45)-C(43)-C(44)	107.9(4)	N(17)-C(59)-C(62)	114.2(3)

C(61)-C(59)-C(62)	108.7(3)
C(60)-C(59)-C(62)	107.6(3)
N(18B)-S(6)-N(16)	104.0(17)
N(18A)-S(6)-N(16)	102.1(3)
N(18B)-S(6)-N(17)	102.3(11)
N(18A)-S(6)-N(17)	103.1(2)
N(16)-S(6)-N(17)	103.37(17)
N(18B)-S(6)-Na(11)	48.9(10)
N(18A)-S(6)-Na(11)	49.3(2)
N(16)-S(6)-Na(11)	101.53(12)
N(17)-S(6)-Na(11)	54.95(11)
N(18B)-S(6)-Na(12)	55.3(16)
N(18A)-S(6)-Na(12)	53.4(3)
N(16)-S(6)-Na(12)	49.74(11)
N(17)-S(6)-Na(12)	102.68(12)
Na(11)-S(6)-Na(12)	63.16(4)
N(18B)-S(6)-Na(10)	101.7(11)
N(18A)-S(6)-Na(10)	100.8(2)
N(16)-S(6)-Na(10)	54.82(13)
N(17)-S(6)-Na(10)	49.88(12)
Na(11)-S(6)-Na(10)	62.76(5)
Na(12)-S(6)-Na(10)	63.62(5)
C(63A)-N(18A)-S(6)	114.7(4)
C(63A)-N(18A)-Na(11)	143.0(4)
S(6)-N(18A)-Na(11)	98.0(2)
C(63A)-N(18A)-Na(12)	109.7(4)
S(6)-N(18A)-Na(12)	93.8(3)
Na(11)-N(18A)-Na(12)	83.6(2)
N(18A)-C(63A)-C(66A)	106.0(4)
N(18A)-C(63A)-C(65A)	113.2(5)
C(66A)-C(63A)-C(65A)	107.8(5)
N(18A)-C(63A)-C(64A)	113.4(5)
C(66A)-C(63A)-C(64A)	107.5(4)
C(65A)-C(63A)-C(64A)	108.7(5)
C(63B)-N(18B)-S(6)	122.8(16)
C(63B)-N(18B)-Na(11)	136.1(15)
S(6)-N(18B)-Na(11)	98.5(10)
C(63B)-N(18B)-Na(12)	108(2)
S(6)-N(18B)-Na(12)	92.0(17)
Na(11)-N(18B)-Na(12)	82.6(10)
N(18B)-C(63B)-C(64B)	110.9(18)
N(18B)-C(63B)-C(65B)	112.7(15)
C(64B)-C(63B)-C(65B)	107.4(15)
N(18B)-C(63B)-C(66B)	109.6(16)
C(64B)-C(63B)-C(66B)	108.8(14)
C(65B)-C(63B)-C(66B)	107.2(15)
C(63B)-C(64B)-Na(12)	87.6(11)
N(19)-C(69)-C(70)	112.4(3)
N(20)-C(70)-C(69)	112.8(4)
N(21)-C(75)-C(76)	111.5(4)
N(22)-C(76)-C(75)	112.8(4)
N(23)-C(81)-C(82)	112.4(4)
N(24)-C(82)-C(81)	112.5(4)

Crystal structure of **5**

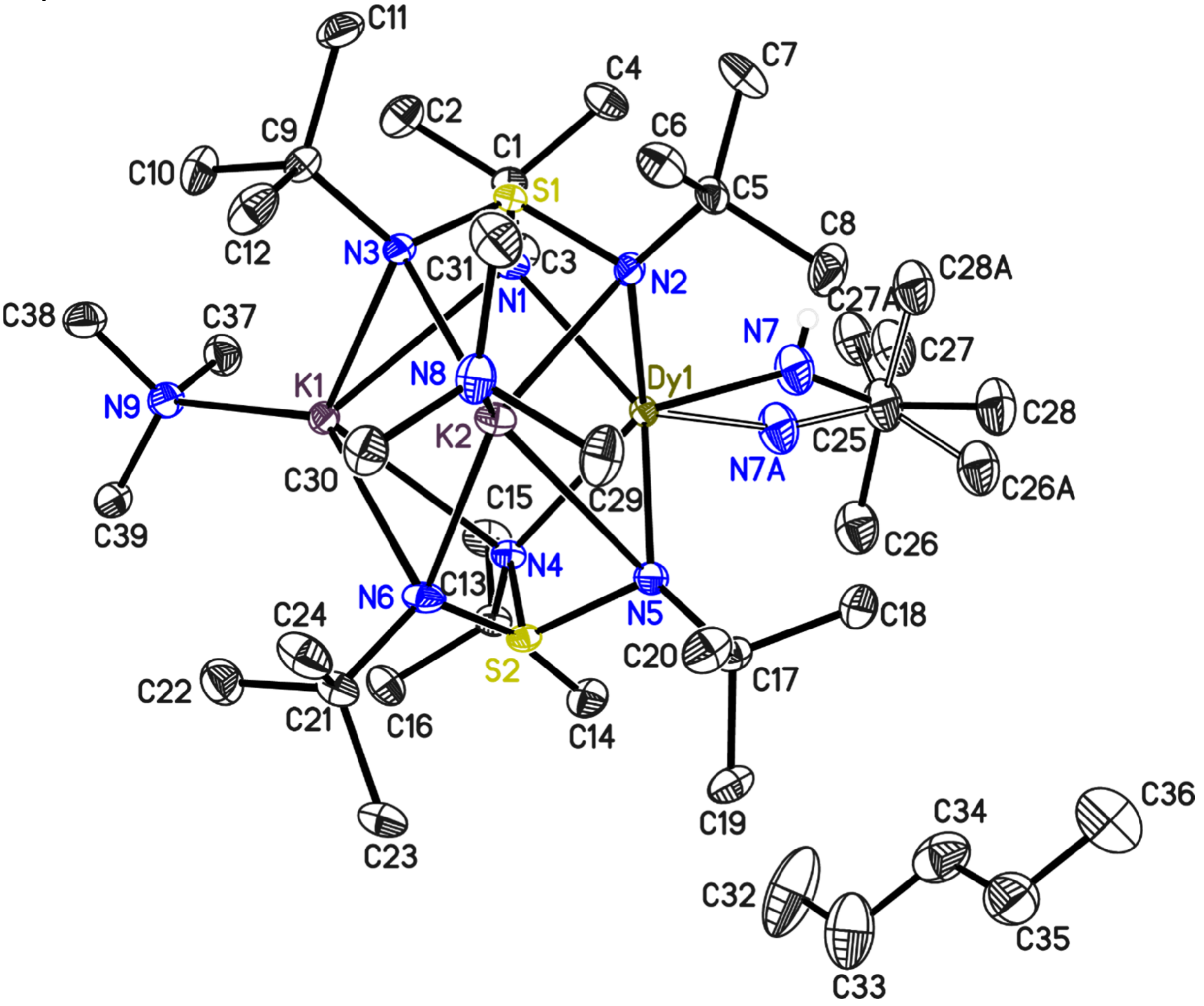

Figure S5. Asymmetric unit of **5** with thermal ellipsoids at 50% probability level. The hydrogen atoms are omitted for clarity, except the hydrogen atom at N(7).

The asymmetric unit shows one entire molecule, while the protonated *t*Butyl-group (N(7)) is disordered over two positions and restraints, on the anisotropic displacement parameters and on distances and angle distances, were applied. The occupancy of the minor position refines to 0.202(4). The hydrogen atom associated with N(7) was treated freely**,** for N(7A) no electron density can be found to place a hydrogen atom. Half a molecule of the bridging tetramethylethylenediamine (tmeda) moiety at K(1) and K(2) is located in the asymmetric unit and each connects two complex molecules. One additional pentane molecule is found in the lattice.

Table S7. Bond lengths [Å] and angles [°] for **5**.

Dy(1)-N(7A)	2.194(9)	N(3)-S(1)-K(2)	48.43(6)
Dy(1)-N(7)	2.199(2)	N(2)-S(1)-K(2)	56.37(6)
Dy(1)-N(5)	2.3239(17)	N(1)-S(1)-K(2)	109.64(6)
Dy(1)-N(2)	2.3297(17)	Dy(1)-S(1)-K(2)	72.85(2)
Dy(1)-N(1)	2.3810(17)	N(3)-S(1)-K(1)	52.85(6)
Dy(1)-N(4)	2.3865(17)	N(2)-S(1)-K(1)	104.44(6)
Dy(1)-S(2)	3.0909(8)	N(1)-S(1)-K(1)	55.18(6)
Dy(1)-S(1)	3.0931(11)	Dy(1)-S(1)-K(1)	69.160(19)
Dy(1)-K(1)	3.7491(9)	K(2)-S(1)-K(1)	70.24(2)
Dy(1)-K(2)	3.9188(10)	N(6)-K(2)-N(3)	92.94(5)
C(1)-N(1)	1.489(2)	N(6)-K(2)-N(2)	102.02(5)
C(1)-C(3)	1.524(3)	N(3)-K(2)-N(2)	55.04(5)
C(1)-C(4)	1.532(3)	N(6)-K(2)-N(8)	132.19(5)
C(1)-C(2)	1.534(3)	N(3)-K(2)-N(8)	117.81(5)
N(1)-S(1)	1.6760(17)	N(2)-K(2)-N(8)	125.36(5)
N(1)-K(1)	2.8807(18)	N(6)-K(2)-N(5)	50.53(5)
K(1)-N(4)	2.7687(18)	N(3)-K(2)-N(5)	102.03(5)
K(1)-N(3)	2.8239(18)	N(2)-K(2)-N(5)	68.31(4)
K(1)-N(6)	2.9942(18)	N(8)-K(2)-N(5)	138.46(5)
K(1)-N(9)	3.0488(19)	N(6)-K(2)-C(20)	71.59(6)
K(1)-C(10)	3.401(2)	N(3)-K(2)-C(20)	144.74(5)
K(1)-S(1)	3.4878(11)	N(2)-K(2)-C(20)	96.36(5)
K(1)-S(2)	3.5431(11)	N(8)-K(2)-C(20)	95.00(5)
K(1)-K(2)	4.0115(11)	N(5)-K(2)-C(20)	43.64(5)
S(1)-N(3)	1.6097(16)	N(6)-K(2)-C(29)	125.44(6)
S(1)-N(2)	1.6724(17)	N(3)-K(2)-C(29)	139.02(6)
S(1)-K(2)	3.4850(9)	N(2)-K(2)-C(29)	119.60(6)
K(2)-N(6)	2.6898(18)	N(8)-K(2)-C(29)	25.70(5)
K(2)-N(3)	2.7004(17)	N(5)-K(2)-C(29)	113.10(5)
K(2)-N(2)	2.9133(18)	C(20)-K(2)-C(29)	70.30(6)
K(2)-N(8)	3.0743(19)	N(6)-K(2)-C(12)	107.66(6)
K(2)-N(5)	3.336(2)	N(3)-K(2)-C(12)	44.06(5)
K(2)-C(20)	3.365(2)	N(2)-K(2)-C(12)	93.09(5)
K(2)-C(29)	3.372(2)	N(8)-K(2)-C(12)	78.41(5)
K(2)-C(12)	3.423(2)	N(5)-K(2)-C(12)	143.08(5)
K(2)-S(2)	3.6599(12)	C(20)-K(2)-C(12)	170.46(6)
C(29)-N(8)	1.463(3)	C(29)-K(2)-C(12)	103.81(6)

N(2)-C(5)	1.484(2)	N(6)-K(2)-S(1)	97.92(4)
S(2)-N(6)	1.6101(17)	N(3)-K(2)-S(1)	26.49(3)
S(2)-N(5)	1.6709(17)	N(2)-K(2)-S(1)	28.55(3)
S(2)-N(4)	1.6719(16)	N(8)-K(2)-S(1)	126.26(4)
N(3)-C(9)	1.476(2)	N(5)-K(2)-S(1)	85.19(4)
C(39)-C(38)	1.507(4)	C(20)-K(2)-S(1)	122.30(4)
C(38)-C(37)	1.517(4)	C(29)-K(2)-S(1)	135.46(5)
C(37)-C(36)	1.491(5)	C(12)-K(2)-S(1)	67.20(4)
C(36)-C(35)	1.521(5)	N(6)-K(2)-S(2)	23.63(4)
N(6)-C(21)	1.479(2)	N(3)-K(2)-S(2)	95.43(4)
C(6)-C(5)	1.531(3)	N(2)-K(2)-S(2)	84.23(4)
N(5)-C(17)	1.475(2)	N(8)-K(2)-S(2)	143.93(4)
C(5)-C(8)	1.519(3)	N(5)-K(2)-S(2)	27.13(3)
C(5)-C(7)	1.531(3)	C(20)-K(2)-S(2)	58.01(4)
N(4)-C(13)	1.486(2)	C(29)-K(2)-S(2)	125.41(5)
N(8)-C(30)	1.465(3)	C(12)-K(2)-S(2)	124.56(5)
N(8)-C(31)	1.466(3)	S(1)-K(2)-S(2)	89.72(2)
N(9)-C(32)	1.465(3)	N(6)-K(2)-Dy(1)	67.20(4)
N(9)-C(34)	1.466(3)	N(3)-K(2)-Dy(1)	68.42(4)
N(9)-C(33)	1.468(3)	N(2)-K(2)-Dy(1)	36.24(3)
C(9)-C(11)	1.527(3)	N(8)-K(2)-Dy(1)	155.63(4)
C(9)-C(12)	1.529(3)	N(5)-K(2)-Dy(1)	36.25(3)
C(9)-C(10)	1.534(3)	C(20)-K(2)-Dy(1)	76.35(4)
C(13)-C(15)	1.526(3)	C(29)-K(2)-Dy(1)	135.23(4)
C(13)-C(16)	1.530(3)	C(12)-K(2)-Dy(1)	112.41(4)
C(13)-C(14)	1.532(3)	S(1)-K(2)-Dy(1)	48.957(18)
C(17)-C(18)	1.529(3)	S(2)-K(2)-Dy(1)	47.99(2)
C(17)-C(20)	1.531(3)	N(6)-K(2)-K(1)	48.28(4)
C(17)-C(19)	1.536(3)	N(3)-K(2)-K(1)	44.66(4)
C(21)-C(23)	1.529(3)	N(2)-K(2)-K(1)	73.32(4)
C(21)-C(24)	1.531(3)	N(8)-K(2)-K(1)	145.59(4)
C(21)-C(22)	1.535(3)	N(5)-K(2)-K(1)	72.74(4)
C(25)-N(7)	1.448(4)	C(20)-K(2)-K(1)	112.61(4)
C(25)-C(27)	1.517(5)	C(29)-K(2)-K(1)	166.85(4)
C(25)-C(28)	1.527(5)	C(12)-K(2)-K(1)	71.39(5)
C(25)-C(26)	1.530(5)	S(1)-K(2)-K(1)	54.912(18)
C(25A)-N(7A)	1.456(15)	S(2)-K(2)-K(1)	54.78(2)
C(25A)-C(26A)	1.512(13)	Dy(1)-K(2)-K(1)	56.413(17)

C(25A)-C(27A)	1.519(13)	N(8)-C(29)-K(2)	65.69(11)
C(25A)-C(28A)	1.534(13)	C(5)-N(2)-S(1)	114.96(13)
C(30)-C(30)#1	1.518(4)	C(5)-N(2)-Dy(1)	138.07(12)
C(33)-C(33)#2	1.513(4)	S(1)-N(2)-Dy(1)	99.92(8)
N(7A)-Dy(1)-N(5)	105.9(3)	C(5)-N(2)-K(2)	102.89(11)
N(7)-Dy(1)-N(5)	117.01(8)	S(1)-N(2)-K(2)	95.07(7)
N(7A)-Dy(1)-N(2)	125.8(3)	Dy(1)-N(2)-K(2)	96.10(6)
N(7)-Dy(1)-N(2)	106.15(10)	N(6)-S(2)-N(5)	106.97(9)
N(5)-Dy(1)-N(2)	98.52(6)	N(6)-S(2)-N(4)	106.75(9)
N(7A)-Dy(1)-N(1)	117.8(3)	N(5)-S(2)-N(4)	95.30(8)
N(7)-Dy(1)-N(1)	107.59(8)	N(6)-S(2)-Dy(1)	103.22(6)
N(5)-Dy(1)-N(1)	135.22(6)	N(5)-S(2)-Dy(1)	47.75(6)
N(2)-Dy(1)-N(1)	63.93(6)	N(4)-S(2)-Dy(1)	49.92(6)
N(7A)-Dy(1)-N(4)	108.5(3)	N(6)-S(2)-K(1)	57.20(6)
N(7)-Dy(1)-N(4)	127.94(9)	N(5)-S(2)-K(1)	109.40(6)
N(5)-Dy(1)-N(4)	63.25(6)	N(4)-S(2)-K(1)	49.57(6)
N(2)-Dy(1)-N(4)	125.73(6)	Dy(1)-S(2)-K(1)	68.43(2)
N(1)-Dy(1)-N(4)	92.81(6)	N(6)-S(2)-K(2)	42.05(6)
N(7A)-Dy(1)-S(2)	117.1(3)	N(5)-S(2)-K(2)	65.56(6)
N(7)-Dy(1)-S(2)	135.57(8)	N(4)-S(2)-K(2)	102.93(6)
N(5)-Dy(1)-S(2)	32.16(4)	Dy(1)-S(2)-K(2)	70.393(19)
N(2)-Dy(1)-S(2)	109.28(4)	K(1)-S(2)-K(2)	67.66(2)
N(1)-Dy(1)-S(2)	111.59(4)	C(9)-N(3)-S(1)	118.78(13)
N(4)-Dy(1)-S(2)	32.42(4)	C(9)-N(3)-K(2)	121.69(12)
N(7A)-Dy(1)-S(1)	133.4(3)	S(1)-N(3)-K(2)	105.08(8)
N(7)-Dy(1)-S(1)	115.07(8)	C(9)-N(3)-K(1)	113.23(12)
N(5)-Dy(1)-S(1)	116.24(5)	S(1)-N(3)-K(1)	100.13(8)
N(2)-Dy(1)-S(1)	32.18(4)	K(2)-N(3)-K(1)	93.10(5)
N(1)-Dy(1)-S(1)	32.47(4)	C(39)-C(38)-C(37)	114.5(3)
N(4)-Dy(1)-S(1)	107.70(4)	C(36)-C(37)-C(38)	114.5(3)
S(2)-Dy(1)-S(1)	109.215(19)	C(37)-C(36)-C(35)	113.8(3)
N(7A)-Dy(1)-K(1)	141.6(3)	C(21)-N(6)-S(2)	118.67(13)
N(7)-Dy(1)-K(1)	148.61(7)	C(21)-N(6)-K(2)	116.92(12)
N(5)-Dy(1)-K(1)	89.42(5)	S(2)-N(6)-K(2)	114.32(9)
N(2)-Dy(1)-K(1)	84.70(5)	C(21)-N(6)-K(1)	115.45(12)
N(1)-Dy(1)-K(1)	50.21(4)	S(2)-N(6)-K(1)	95.93(7)
N(4)-Dy(1)-K(1)	47.52(4)	K(2)-N(6)-K(1)	89.61(5)
S(2)-Dy(1)-K(1)	61.51(2)	C(17)-N(5)-S(2)	116.26(13)

S(1)-Dy(1)-K(1)	60.39(2)	C(17)-N(5)-Dy(1)	142.72(13)
N(7A)-Dy(1)-K(2)	154.1(3)	S(2)-N(5)-Dy(1)	100.10(8)
N(7)-Dy(1)-K(2)	144.60(8)	C(17)-N(5)-K(2)	103.20(11)
N(5)-Dy(1)-K(2)	58.08(5)	S(2)-N(5)-K(2)	87.30(7)
N(2)-Dy(1)-K(2)	47.66(5)	Dy(1)-N(5)-K(2)	85.67(5)
N(1)-Dy(1)-K(2)	83.40(4)	N(2)-C(5)-C(8)	105.94(16)
N(4)-Dy(1)-K(2)	83.48(4)	N(2)-C(5)-C(7)	112.50(17)
S(2)-Dy(1)-K(2)	61.62(2)	C(8)-C(5)-C(7)	108.82(19)
S(1)-Dy(1)-K(2)	58.19(2)	N(2)-C(5)-C(6)	113.27(16)
K(1)-Dy(1)-K(2)	63.042(17)	C(8)-C(5)-C(6)	108.43(18)
N(1)-C(1)-C(3)	106.40(15)	C(7)-C(5)-C(6)	107.73(17)
N(1)-C(1)-C(4)	113.35(16)	C(13)-N(4)-S(2)	112.67(12)
C(3)-C(1)-C(4)	108.15(17)	C(13)-N(4)-Dy(1)	132.12(12)
N(1)-C(1)-C(2)	111.94(16)	S(2)-N(4)-Dy(1)	97.66(7)
C(3)-C(1)-C(2)	108.75(17)	C(13)-N(4)-K(1)	113.69(11)
C(4)-C(1)-C(2)	108.11(17)	S(2)-N(4)-K(1)	103.06(7)
C(1)-N(1)-S(1)	112.59(12)	Dy(1)-N(4)-K(1)	93.02(5)
C(1)-N(1)-Dy(1)	129.42(12)	C(29)-N(8)-C(30)	108.54(16)
S(1)-N(1)-Dy(1)	97.84(8)	C(29)-N(8)-C(31)	109.07(18)
C(1)-N(1)-K(1)	123.25(12)	C(30)-N(8)-C(31)	110.82(16)
S(1)-N(1)-K(1)	96.28(7)	C(29)-N(8)-K(2)	88.60(12)
Dy(1)-N(1)-K(1)	90.37(5)	C(30)-N(8)-K(2)	118.24(12)
N(4)-K(1)-N(3)	105.65(5)	C(31)-N(8)-K(2)	118.48(13)
N(4)-K(1)-N(1)	75.32(5)	C(32)-N(9)-C(34)	108.73(17)
N(3)-K(1)-N(1)	55.49(5)	C(32)-N(9)-C(33)	111.29(17)
N(4)-K(1)-N(6)	54.23(5)	C(34)-N(9)-C(33)	108.05(17)
N(3)-K(1)-N(6)	84.34(5)	C(32)-N(9)-K(1)	100.50(12)
N(1)-K(1)-N(6)	102.65(5)	C(34)-N(9)-K(1)	110.91(12)
N(4)-K(1)-N(9)	114.51(5)	C(33)-N(9)-K(1)	117.00(12)
N(3)-K(1)-N(9)	131.93(5)	N(3)-C(9)-C(11)	115.54(17)
N(1)-K(1)-N(9)	110.24(5)	N(3)-C(9)-C(12)	105.54(16)
N(6)-K(1)-N(9)	140.86(5)	C(11)-C(9)-C(12)	108.26(18)
N(4)-K(1)-C(10)	150.87(5)	N(3)-C(9)-C(10)	111.01(16)
N(3)-K(1)-C(10)	45.81(5)	C(11)-C(9)-C(10)	108.37(17)
N(1)-K(1)-C(10)	81.07(5)	C(12)-C(9)-C(10)	107.82(18)
N(6)-K(1)-C(10)	116.66(5)	C(9)-C(10)-K(1)	87.35(11)
N(9)-K(1)-C(10)	89.45(5)	C(9)-C(12)-K(2)	87.81(11)
N(4)-K(1)-S(1)	89.76(4)	N(4)-C(13)-C(15)	105.88(15)

N(3)-K(1)-S(1)	27.02(3)	N(4)-C(13)-C(16)	112.12(16)
N(1)-K(1)-S(1)	28.53(3)	C(15)-C(13)-C(16)	108.11(17)
N(6)-K(1)-S(1)	92.29(4)	N(4)-C(13)-C(14)	113.00(16)
N(9)-K(1)-S(1)	126.58(4)	C(15)-C(13)-C(14)	108.69(17)
C(10)-K(1)-S(1)	61.80(4)	C(16)-C(13)-C(14)	108.85(17)
N(4)-K(1)-S(2)	27.37(3)	N(5)-C(17)-C(18)	106.53(16)
N(3)-K(1)-S(2)	95.83(4)	N(5)-C(17)-C(20)	111.91(16)
N(1)-K(1)-S(2)	89.40(4)	C(18)-C(17)-C(20)	109.09(17)
N(6)-K(1)-S(2)	26.87(3)	N(5)-C(17)-C(19)	113.29(16)
N(9)-K(1)-S(2)	131.72(4)	C(18)-C(17)-C(19)	107.23(17)
C(10)-K(1)-S(2)	138.12(4)	C(20)-C(17)-C(19)	108.60(17)
S(1)-K(1)-S(2)	91.61(2)	C(17)-C(20)-K(2)	100.54(12)
N(4)-K(1)-Dy(1)	39.47(3)	N(6)-C(21)-C(23)	115.69(17)
N(3)-K(1)-Dy(1)	70.22(4)	N(6)-C(21)-C(24)	106.11(16)
N(1)-K(1)-Dy(1)	39.43(4)	C(23)-C(21)-C(24)	108.21(18)
N(6)-K(1)-Dy(1)	67.48(4)	N(6)-C(21)-C(22)	110.43(17)
N(9)-K(1)-Dy(1)	131.41(4)	C(23)-C(21)-C(22)	108.49(17)
C(10)-K(1)-Dy(1)	112.20(5)	C(24)-C(21)-C(22)	107.59(18)
S(1)-K(1)-Dy(1)	50.45(3)	N(7)-C(25)-C(27)	112.6(3)
S(2)-K(1)-Dy(1)	50.061(18)	N(7)-C(25)-C(28)	111.5(3)
N(4)-K(1)-K(2)	77.45(4)	C(27)-C(25)-C(28)	108.3(3)
N(3)-K(1)-K(2)	42.24(4)	N(7)-C(25)-C(26)	106.7(3)
N(1)-K(1)-K(2)	76.21(4)	C(27)-C(25)-C(26)	109.1(3)
N(6)-K(1)-K(2)	42.11(3)	C(28)-C(25)-C(26)	108.6(3)
N(9)-K(1)-K(2)	167.25(4)	C(25)-N(7)-Dy(1)	147.4(3)
C(10)-K(1)-K(2)	80.58(4)	N(7A)-C(25A)-C(26A)	111.8(11)
S(1)-K(1)-K(2)	54.85(2)	N(7A)-C(25A)-C(27A)	110.6(12)
S(2)-K(1)-K(2)	57.56(2)	C(26A)-C(25A)-C(27A)	111.3(13)
Dy(1)-K(1)-K(2)	60.55(2)	N(7A)-C(25A)-C(28A)	107.0(11)
N(3)-S(1)-N(2)	104.80(9)	C(26A)-C(25A)-C(28A)	108.9(12)
N(3)-S(1)-N(1)	107.85(9)	C(27A)-C(25A)-C(28A)	107.0(13)
N(2)-S(1)-N(1)	96.30(8)	C(25A)-N(7A)-Dy(1)	147.1(9)
N(3)-S(1)-Dy(1)	105.77(6)	N(8)-C(30)-C(30)#1	112.9(2)
N(2)-S(1)-Dy(1)	47.90(6)	N(9)-C(33)-C(33)#2	113.0(2)
N(1)-S(1)-Dy(1)	49.69(6)		

Crystal structure of **6**

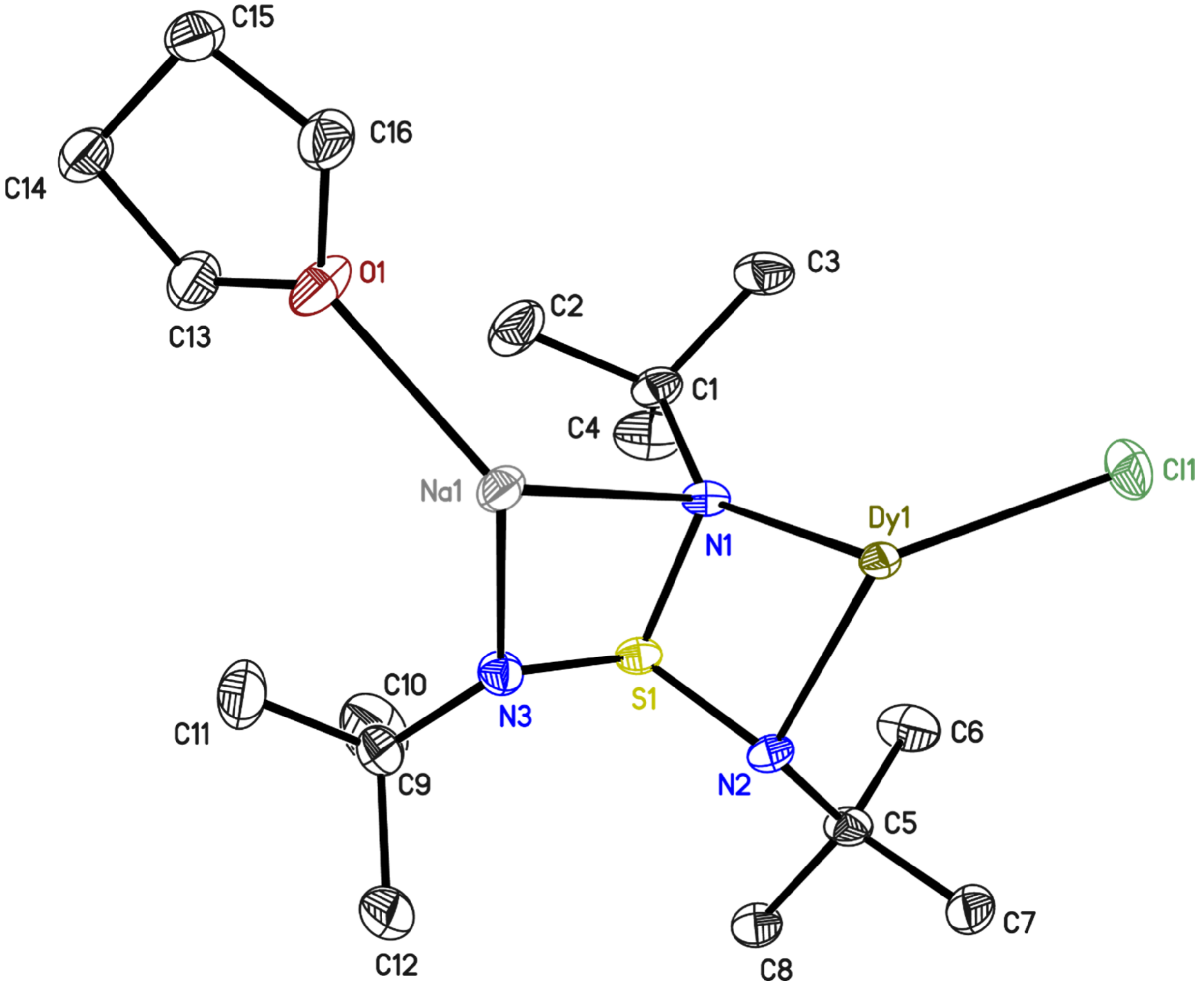

Figure S6. Asymmetric unit of **6** with thermal ellipsoids at 50% probability level. The hydrogen atoms are omitted for clarity.

The asymmetric unit shows half a molecule, while none of the *t*Butyl-group or THF molecules are disordered. Dy(1) and Cl(1) are located on a 2-fold axis.

Table S8. Bond lengths [Å] and angles [°] for **6**.

Dy(1)-N(1)	2.2985(13)	N(3)-Na(1)-S(1)	27.06(3)
Dy(1)-N(2)	2.3702(13)	N(2)#1-Na(1)-S(1)	95.14(4)
Dy(1)-Cl(1)	2.5564(6)	N(3)#1-Na(1)-S(1)	97.43(4)
Dy(1)-S(1)	3.0476(6)	N(1)-Na(1)-S(1)	29.98(2)
Dy(1)-Na(1)	3.5151(7)	C(12)#1-Na(1)-S(1)	141.84(4)
O(1)-C(13)	1.431(2)	S(1)#1-Na(1)-S(1)	97.94(2)
O(1)-C(16)	1.437(2)	O(1)-Na(1)-Na(1)#1	157.47(4)
O(1)-Na(1)	2.3690(13)	N(3)-Na(1)-Na(1)#1	49.45(4)
Na(1)-N(3)	2.4002(14)	N(2)#1-Na(1)-Na(1)#1	82.28(3)
Na(1)-N(2)#1	2.4736(14)	N(3)#1-Na(1)-Na(1)#1	43.85(3)
Na(1)-N(3)#1	2.6325(15)	N(1)-Na(1)-Na(1)#1	77.11(3)
Na(1)-N(1)	3.0012(15)	C(12)#1-Na(1)-Na(1)#1	87.39(4)
Na(1)-C(12)#1	3.067(2)	S(1)#1-Na(1)-Na(1)#1	60.02(2)
Na(1)-S(1)#1	3.1737(8)	S(1)-Na(1)-Na(1)#1	55.733(19)
Na(1)-S(1)	3.3263(9)	O(1)-Na(1)-Dy(1)	142.00(4)
Na(1)-Na(1)#1	3.4590(14)	N(3)-Na(1)-Dy(1)	75.68(3)
C(1)-N(1)	1.4853(18)	N(2)#1-Na(1)-Dy(1)	42.33(3)
C(1)-C(3)	1.530(2)	N(3)#1-Na(1)-Dy(1)	73.22(3)
C(1)-C(2)	1.532(2)	N(1)-Na(1)-Dy(1)	40.35(3)
C(1)-C(4)	1.533(2)	C(12)#1-Na(1)-Dy(1)	120.47(4)
N(1)-S(1)	1.6667(13)	S(1)#1-Na(1)-Dy(1)	53.916(14)
S(1)-N(3)	1.6141(13)	S(1)-Na(1)-Dy(1)	52.819(13)
S(1)-N(2)	1.6875(13)	Na(1)#1-Na(1)-Dy(1)	60.527(11)
N(3)-C(9)	1.485(2)	N(1)-C(1)-C(3)	106.18(13)
N(2)-C(5)	1.4960(18)	N(1)-C(1)-C(2)	112.07(13)
C(16)-C(15)	1.516(2)	C(3)-C(1)-C(2)	108.85(14)
C(15)-C(14)	1.518(2)	N(1)-C(1)-C(4)	112.77(13)
C(14)-C(13)	1.505(2)	C(3)-C(1)-C(4)	107.94(14)
C(12)-C(9)	1.535(2)	C(2)-C(1)-C(4)	108.86(15)
C(11)-C(9)	1.515(3)	C(1)-N(1)-S(1)	116.01(10)
C(10)-C(9)	1.540(3)	C(1)-N(1)-Dy(1)	144.23(10)
C(5)-C(7)	1.525(2)	S(1)-N(1)-Dy(1)	99.22(6)
C(5)-C(8)	1.532(2)	C(1)-N(1)-Na(1)	105.52(9)
C(5)-C(6)	1.539(2)	S(1)-N(1)-Na(1)	85.87(5)
N(1)-Dy(1)-N(1)#1	138.66(6)	Dy(1)-N(1)-Na(1)	81.94(4)
N(1)-Dy(1)-N(2)	64.82(4)	N(3)-S(1)-N(1)	105.21(7)
N(1)#1-Dy(1)-N(2)	94.80(5)	N(3)-S(1)-N(2)	106.44(7)

N(2)-Dy(1)-N(2)#1	122.09(6)
N(1)-Dy(1)-Cl(1)	110.67(3)
N(2)-Dy(1)-Cl(1)	118.96(3)
N(1)-Dy(1)-S(1)#1	115.00(3)
N(2)-Dy(1)-S(1)#1	105.05(3)
Cl(1)-Dy(1)-S(1)#1	126.426(9)
N(1)-Dy(1)-S(1)	32.67(3)
N(2)-Dy(1)-S(1)	33.42(3)
Cl(1)-Dy(1)-S(1)	126.426(9)
S(1)#1-Dy(1)-S(1)	107.147(19)
N(1)-Dy(1)-Na(1)	57.71(3)
N(1)#1-Dy(1)-Na(1)	85.39(3)
N(2)-Dy(1)-Na(1)	82.44(3)
N(2)#1-Dy(1)-Na(1)	44.64(3)
Cl(1)-Dy(1)-Na(1)	150.527(11)
S(1)#1-Dy(1)-Na(1)	57.310(14)
S(1)-Dy(1)-Na(1)	60.409(15)
Na(1)-Dy(1)-Na(1)#1	58.95(2)
C(13)-O(1)-C(16)	109.21(12)
C(13)-O(1)-Na(1)	124.16(10)
C(16)-O(1)-Na(1)	126.52(10)
O(1)-Na(1)-N(3)	124.73(5)
O(1)-Na(1)-N(2)#1	114.80(5)
N(3)-Na(1)-N(2)#1	116.43(5)
O(1)-Na(1)-N(3)#1	129.49(5)
N(3)-Na(1)-N(3)#1	92.57(5)
N(2)#1-Na(1)-N(3)#1	62.30(4)
O(1)-Na(1)-N(1)	119.61(5)
N(3)-Na(1)-N(1)	56.40(4)
N(2)#1-Na(1)-N(1)	77.11(4)
N(3)#1-Na(1)-N(1)	108.99(4)
O(1)-Na(1)-C(12)#1	78.92(5)
N(3)-Na(1)-C(12)#1	121.22(5)
N(2)#1-Na(1)-C(12)#1	88.46(5)
N(3)#1-Na(1)-C(12)#1	51.34(5)
N(1)-Na(1)-C(12)#1	160.00(5)
O(1)-Na(1)-S(1)#1	127.69(4)
N(3)-Na(1)-S(1)#1	106.89(4)
N(1)-S(1)-N(2)	96.53(6)
N(3)-S(1)-Dy(1)	102.03(5)
N(1)-S(1)-Dy(1)	48.11(4)
N(2)-S(1)-Dy(1)	50.68(4)
N(3)-S(1)-Na(1)#1	55.88(5)
N(1)-S(1)-Na(1)#1	109.27(5)
N(2)-S(1)-Na(1)#1	50.58(5)
Dy(1)-S(1)-Na(1)#1	68.773(17)
N(3)-S(1)-Na(1)	42.57(5)
N(1)-S(1)-Na(1)	64.15(5)
N(2)-S(1)-Na(1)	99.59(5)
Dy(1)-S(1)-Na(1)	66.772(18)
Na(1)#1-S(1)-Na(1)	64.25(2)
C(9)-N(3)-S(1)	116.65(10)
C(9)-N(3)-Na(1)	129.45(10)
S(1)-N(3)-Na(1)	110.37(7)
C(9)-N(3)-Na(1)#1	107.21(9)
S(1)-N(3)-Na(1)#1	93.62(6)
Na(1)-N(3)-Na(1)#1	86.70(5)
C(5)-N(2)-S(1)	112.02(10)
C(5)-N(2)-Dy(1)	121.87(9)
S(1)-N(2)-Dy(1)	95.90(5)
C(5)-N(2)-Na(1)#1	129.47(9)
S(1)-N(2)-Na(1)#1	97.62(6)
Dy(1)-N(2)-Na(1)#1	93.03(5)
O(1)-C(16)-C(15)	107.31(13)
C(16)-C(15)-C(14)	103.00(13)
C(13)-C(14)-C(15)	102.80(14)
O(1)-C(13)-C(14)	106.64(14)
C(9)-C(12)-Na(1)#1	88.10(10)
N(3)-C(9)-C(11)	106.16(14)
N(3)-C(9)-C(12)	111.71(14)
C(11)-C(9)-C(12)	108.01(16)
N(3)-C(9)-C(10)	114.57(15)
C(11)-C(9)-C(10)	109.66(18)
C(12)-C(9)-C(10)	106.58(16)
N(2)-C(5)-C(7)	106.14(12)
N(2)-C(5)-C(8)	111.57(13)

N(2)#1-Na(1)-S(1)#1	31.80(3)	C(7)-C(5)-C(8)	109.24(13)
N(3)#1-Na(1)-S(1)#1	30.50(3)	N(2)-C(5)-C(6)	113.22(12)
N(1)-Na(1)-S(1)#1	94.25(3)	C(7)-C(5)-C(6)	107.90(14)
C(12)#1-Na(1)-S(1)#1	66.78(4)	C(8)-C(5)-C(6)	108.63(14)
O(1)-Na(1)-S(1)	131.70(4)		

Symmetry transformations used to generate equivalent atoms:

#1 -x+1,y,-z+1/2

Crystal structure of **7**

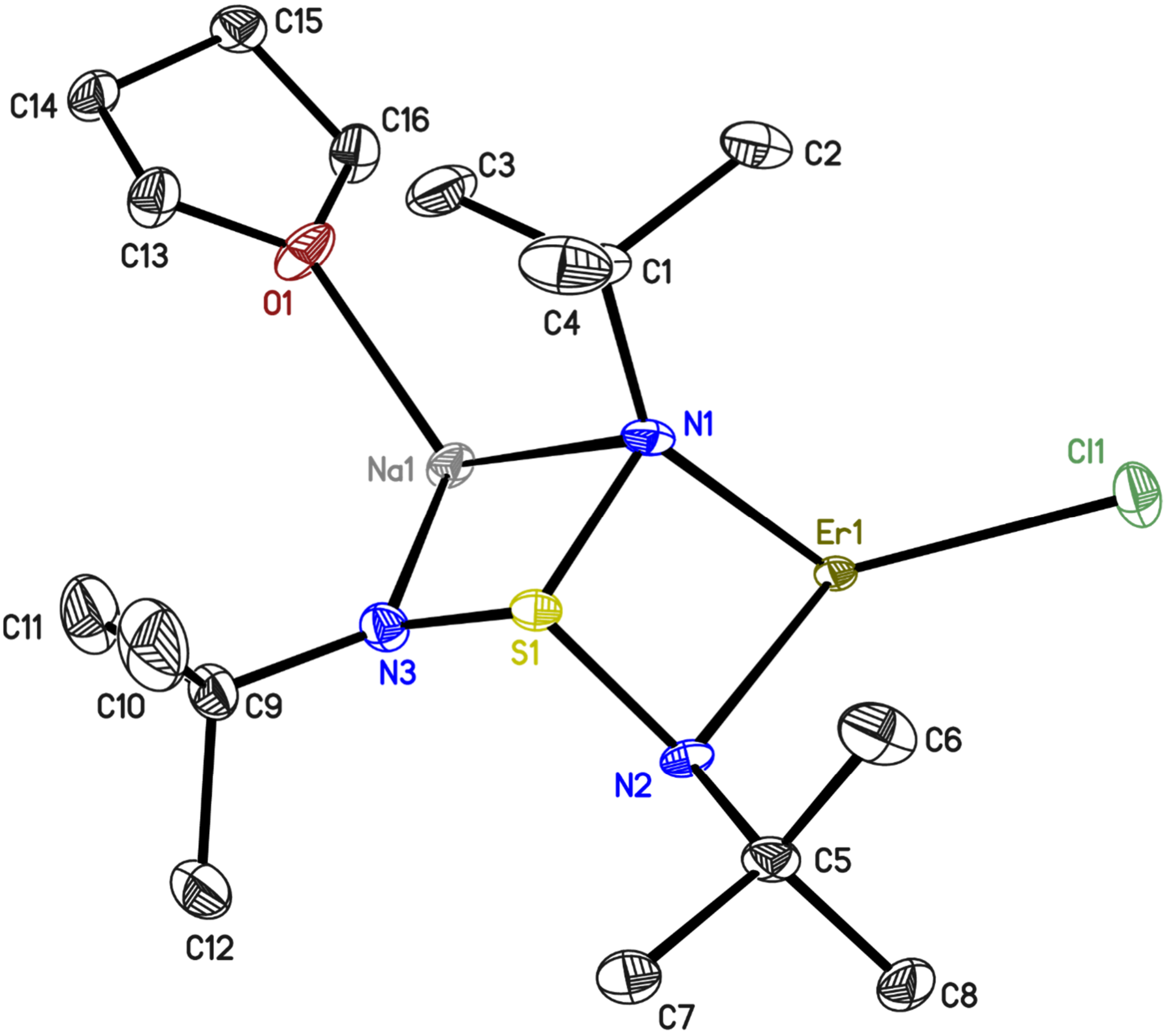

Figure S7. Asymmetric unit of **7** with thermal ellipsoids at 50% probability level. The hydrogen atoms are omitted for clarity.

The asymmetric unit of **7** shows half a molecule, while none of the *t*Butyl-group or THF molecules are disordered. Er(1) and Cl(1) are located on a 2-fold axis.

Table S9. Bond lengths [Å] and angles [°] for **7**.

Cl(1)-Er(1)	2.5203(8)	N(3)#1-Na(1)-S(1)	97.47(4)
Er(1)-N(1)	2.2868(16)	N(1)-Na(1)-S(1)	30.05(3)
Er(1)-N(2)	2.3403(16)	C(12)#1-Na(1)-S(1)	141.84(5)
Er(1)-S(1)	3.0262(6)	S(1)#1-Na(1)-S(1)	97.71(3)
Er(1)-Na(1)	3.4965(9)	O(1)-Na(1)-Na(1)#1	157.15(5)
Na(1)-O(1)	2.3714(16)	N(3)-Na(1)-Na(1)#1	49.36(4)
Na(1)-N(3)	2.3982(17)	N(2)#1-Na(1)-Na(1)#1	82.02(4)
Na(1)-N(2)#1	2.4740(18)	N(3)#1-Na(1)-Na(1)#1	43.86(4)
Na(1)-N(3)#1	2.6267(18)	N(1)-Na(1)-Na(1)#1	77.25(3)
Na(1)-N(1)	2.9836(18)	C(12)#1-Na(1)-Na(1)#1	87.32(5)
Na(1)-C(12)#1	3.064(3)	S(1)#1-Na(1)-Na(1)#1	59.83(3)
Na(1)-S(1)#1	3.1697(9)	S(1)-Na(1)-Na(1)#1	55.79(2)
Na(1)-S(1)	3.3135(10)	O(1)-Na(1)-Er(1)	142.46(5)
Na(1)-Na(1)#1	3.4559(16)	N(3)-Na(1)-Er(1)	75.55(4)
O(1)-C(13)	1.431(2)	N(2)#1-Na(1)-Er(1)	41.94(4)
O(1)-C(16)	1.438(2)	N(3)#1-Na(1)-Er(1)	73.13(4)
C(1)-N(1)	1.483(2)	N(1)-Na(1)-Er(1)	40.36(3)
C(1)-C(2)	1.528(3)	C(12)#1-Na(1)-Er(1)	120.43(5)
C(1)-C(3)	1.529(3)	S(1)#1-Na(1)-Er(1)	53.726(16)
C(1)-C(4)	1.532(3)	S(1)-Na(1)-Er(1)	52.684(15)
N(1)-S(1)	1.6632(15)	Na(1)#1-Na(1)-Er(1)	60.384(14)
S(1)-N(3)	1.6102(16)	C(13)-O(1)-C(16)	109.26(15)
S(1)-N(2)	1.6892(16)	C(13)-O(1)-Na(1)	124.31(12)
N(3)-C(9)	1.482(2)	C(16)-O(1)-Na(1)	126.25(12)
N(2)-C(5)	1.493(2)	N(1)-C(1)-C(2)	106.30(16)
C(5)-C(8)	1.524(3)	N(1)-C(1)-C(3)	112.15(16)
C(5)-C(7)	1.529(3)	C(2)-C(1)-C(3)	108.82(17)
C(5)-C(6)	1.537(3)	N(1)-C(1)-C(4)	112.83(15)
C(15)-C(16)	1.516(3)	C(2)-C(1)-C(4)	107.76(17)
C(15)-C(14)	1.518(3)	C(3)-C(1)-C(4)	108.80(19)
C(14)-C(13)	1.509(3)	C(1)-N(1)-S(1)	115.88(13)
C(12)-C(9)	1.534(3)	C(1)-N(1)-Er(1)	144.78(12)
C(11)-C(9)	1.518(3)	S(1)-N(1)-Er(1)	98.79(7)
C(10)-C(9)	1.542(3)	C(1)-N(1)-Na(1)	105.35(10)
N(1)-Er(1)-N(1)#1	139.10(8)	S(1)-N(1)-Na(1)	86.02(6)
N(1)-Er(1)-N(2)#1	94.80(6)	Er(1)-N(1)-Na(1)	81.97(5)
N(1)-Er(1)-N(2)	65.32(5)	N(3)-S(1)-N(1)	105.32(8)

N(2)#1-Er(1)-N(2)	122.93(8)	N(3)-S(1)-N(2)	106.47(8)
N(1)-Er(1)-Cl(1)	110.45(4)	N(1)-S(1)-N(2)	96.32(8)
N(2)-Er(1)-Cl(1)	118.54(4)	N(3)-S(1)-Er(1)	102.10(6)
N(1)-Er(1)-S(1)	32.90(4)	N(1)-S(1)-Er(1)	48.31(5)
N(1)#1-Er(1)-S(1)	115.27(4)	N(2)-S(1)-Er(1)	50.27(5)
N(2)#1-Er(1)-S(1)	105.50(4)	N(3)-S(1)-Na(1)#1	55.78(6)
N(2)-Er(1)-S(1)	33.72(4)	N(1)-S(1)-Na(1)#1	109.27(6)
Cl(1)-Er(1)-S(1)	126.216(10)	N(2)-S(1)-Na(1)#1	50.71(6)
N(1)-Er(1)-S(1)#1	115.26(4)	Er(1)-S(1)-Na(1)#1	68.66(2)
S(1)-Er(1)-S(1)#1	107.57(2)	N(3)-S(1)-Na(1)	42.87(6)
N(1)-Er(1)-Na(1)	57.67(4)	N(1)-S(1)-Na(1)	63.93(6)
N(1)#1-Er(1)-Na(1)	85.84(4)	N(2)-S(1)-Na(1)	99.55(6)
N(2)#1-Er(1)-Na(1)	44.95(4)	Er(1)-S(1)-Na(1)	66.76(2)
N(2)-Er(1)-Na(1)	82.94(4)	Na(1)#1-S(1)-Na(1)	64.38(3)
Cl(1)-Er(1)-Na(1)	150.384(14)	C(9)-N(3)-S(1)	116.94(13)
S(1)-Er(1)-Na(1)	60.552(18)	C(9)-N(3)-Na(1)	129.39(12)
S(1)#1-Er(1)-Na(1)	57.610(17)	S(1)-N(3)-Na(1)	109.95(8)
N(1)-Er(1)-Na(1)#1	85.84(4)	C(9)-N(3)-Na(1)#1	107.41(11)
Na(1)-Er(1)-Na(1)#1	59.23(3)	S(1)-N(3)-Na(1)#1	93.77(7)
O(1)-Na(1)-N(3)	124.81(6)	Na(1)-N(3)-Na(1)#1	86.78(6)
O(1)-Na(1)-N(2)#1	115.28(6)	C(5)-N(2)-S(1)	111.83(12)
N(3)-Na(1)-N(2)#1	115.96(6)	C(5)-N(2)-Er(1)	122.71(12)
O(1)-Na(1)-N(3)#1	129.22(6)	S(1)-N(2)-Er(1)	96.01(7)
N(3)-Na(1)-N(3)#1	92.49(6)	C(5)-N(2)-Na(1)#1	128.88(11)
N(2)#1-Na(1)-N(3)#1	62.35(5)	S(1)-N(2)-Na(1)#1	97.38(7)
O(1)-Na(1)-N(1)	119.83(6)	Er(1)-N(2)-Na(1)#1	93.11(6)
N(3)-Na(1)-N(1)	56.59(5)	N(2)-C(5)-C(8)	106.21(15)
N(2)#1-Na(1)-N(1)	76.60(5)	N(2)-C(5)-C(7)	111.73(16)
N(3)#1-Na(1)-N(1)	109.06(5)	C(8)-C(5)-C(7)	109.17(15)
O(1)-Na(1)-C(12)#1	78.67(6)	N(2)-C(5)-C(6)	113.22(15)
N(3)-Na(1)-C(12)#1	121.06(7)	C(8)-C(5)-C(6)	107.98(17)
N(2)#1-Na(1)-C(12)#1	88.87(6)	C(7)-C(5)-C(6)	108.39(16)
N(3)#1-Na(1)-C(12)#1	51.30(6)	C(16)-C(15)-C(14)	103.04(16)
N(1)-Na(1)-C(12)#1	160.02(6)	C(13)-C(14)-C(15)	102.44(16)
O(1)-Na(1)-S(1)#1	127.84(5)	O(1)-C(13)-C(14)	106.33(16)
N(3)-Na(1)-S(1)#1	106.60(5)	C(9)-C(12)-Na(1)#1	88.08(12)
N(2)#1-Na(1)-S(1)#1	31.91(4)	N(3)-C(9)-C(11)	106.33(17)
N(3)#1-Na(1)-S(1)#1	30.46(4)	N(3)-C(9)-C(12)	111.62(16)

N(1)-Na(1)-S(1)#1	94.08(4)	C(11)-C(9)-C(12)	108.17(19)
C(12)#1-Na(1)-S(1)#1	66.93(5)	N(3)-C(9)-C(10)	114.62(18)
O(1)-Na(1)-S(1)	131.88(5)	C(11)-C(9)-C(10)	109.0(2)
N(3)-Na(1)-S(1)	27.18(4)	C(12)-C(9)-C(10)	106.91(19)
N(2)#1-Na(1)-S(1)	94.62(4)	O(1)-C(16)-C(15)	107.15(16)

Symmetry transformations used to generate equivalent atoms:

#1 -x+1,y,-z+3/2

S2 Analytical details

Yield calculation procedure

The yield determination is exemplified for **2** and was performed in the following way:

3.0 eq. of *t*BuNSN*t*Bu are required to form 1.0 eq. of [S(*t*BuN)$_3$(thf)$_3$-Na$_3$SNa$_3$(thf)$_3$(N*t*Bu)$_3$S] (**2**), which allows a maximum theoretical yield of 33.3 %. The actual yield was determined to be 24 %, which was applied in the following equation to calculate the overall yield:

$$yield\ (\%) = \frac{actual\ yield}{theoretical\ yield} \cdot 100\% = \frac{24}{33.3} \cdot 100\% = 72\ \%$$

NMR-spectroscopy data

The ^{1}H and ^{13}C NMR spectroscopic data were recorded at our Central Analytics Department, , located at the organic chemistry institute at University of Göttingen. All measurements were performed on a Bruker Avance 500 MHz, a Bruker Avance 400 MHz and a Bruker Avance 300 MHz spectrometer and referenced to a deuterated solvent signal (thf-*d*$_8$), (benzene-*d*$_6$), and (toluene-*d*$_8$).

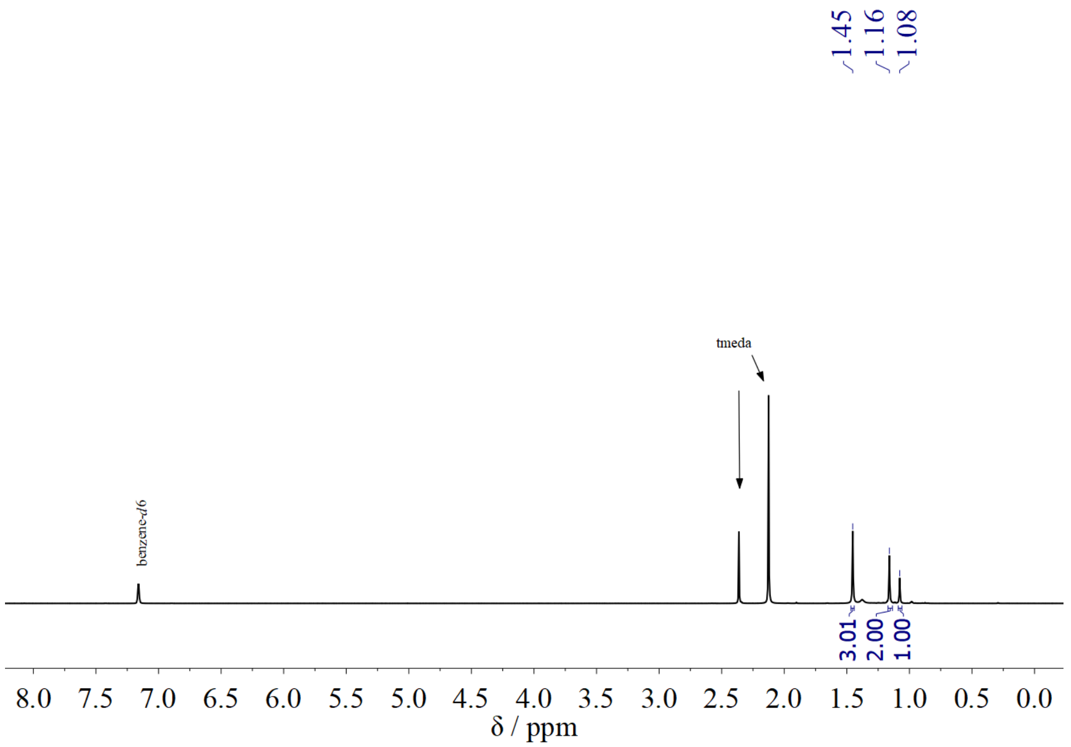

Figure S8. ^{1}H-NMR data for **1** in benzene-*d*$_6$ at 298 K. Signal distribution of 3:2:1, referring to one, two and three equal *t*butyl-groups. The hydrogen atom at the nitrogen could not be detected due to fast exchange processes.

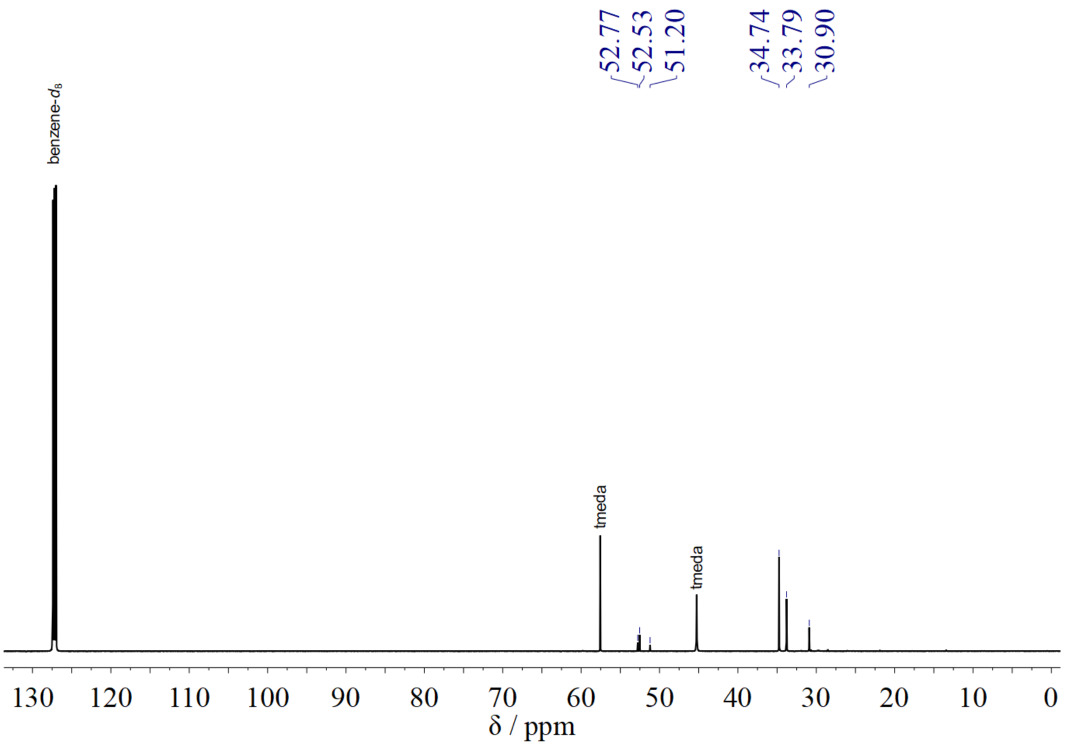

Figure S9. ^{13}C-NMR data for **1** in benzene-d_6 at 298 K. The Residual solvent signals are labeled with # and grease is labeled with *.

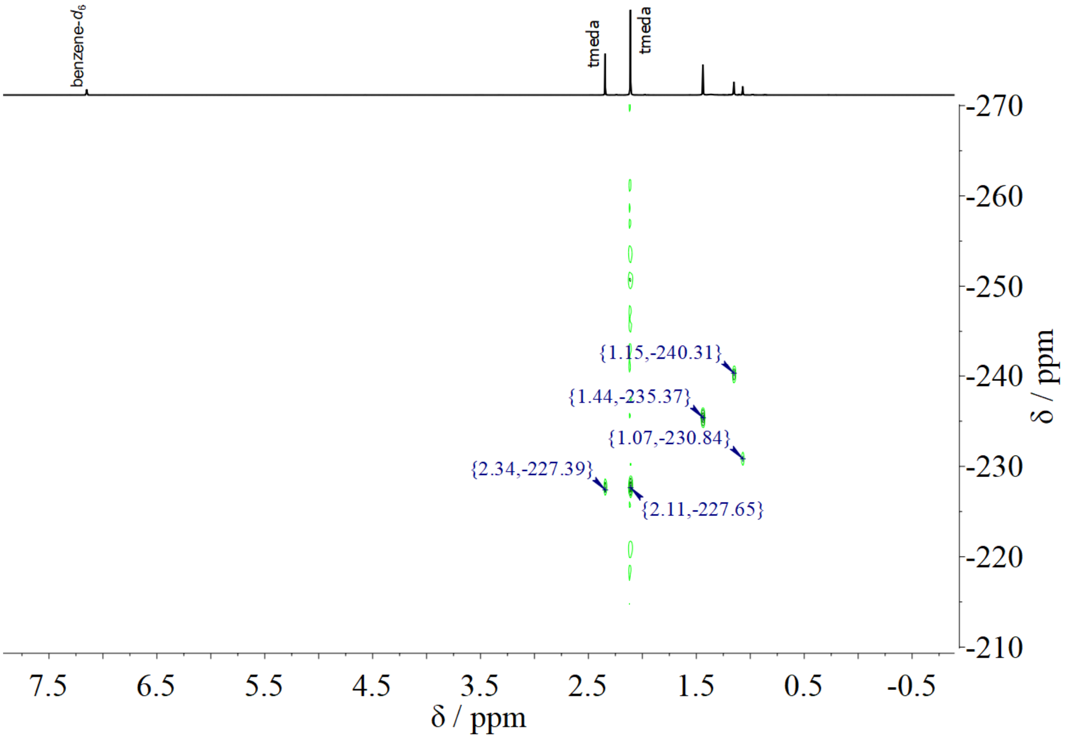

Figure S10. $^{15}N\{H\}$-HMBC-NMR data for **1** in benzene-d_6 at 298 K.

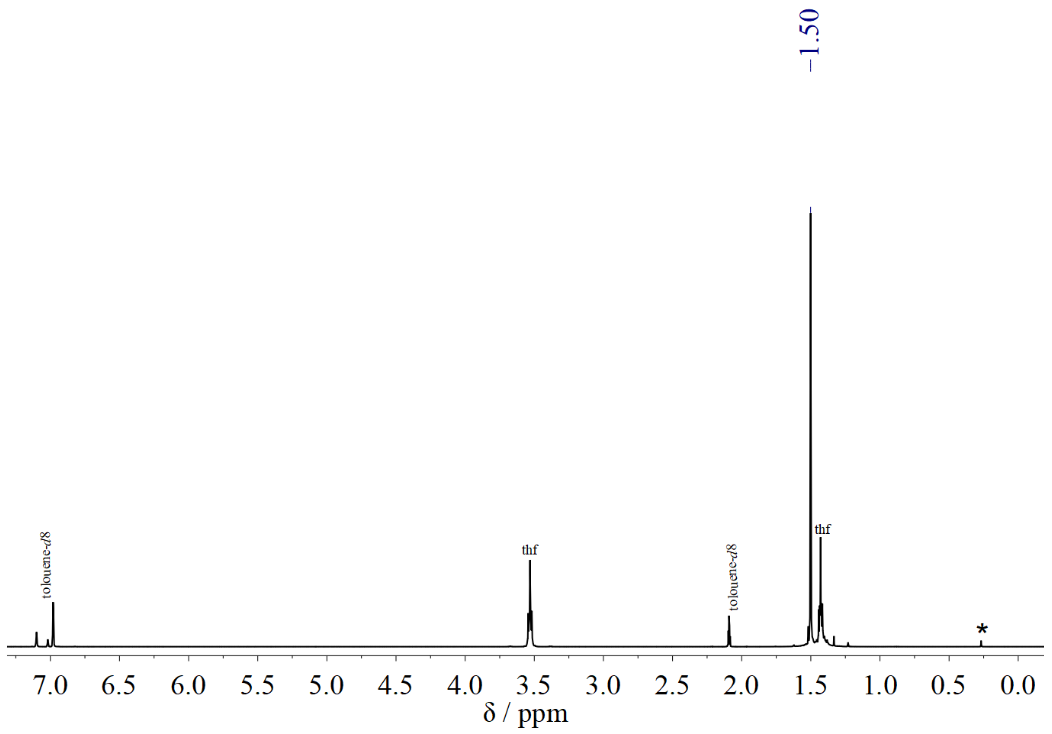

Figure S11. ^{1}H-NMR data for **2** in toluene-d_8 at 298 K. The Residual solvent signals are referred to coordinated thf. The remaining grease is labeled with *.

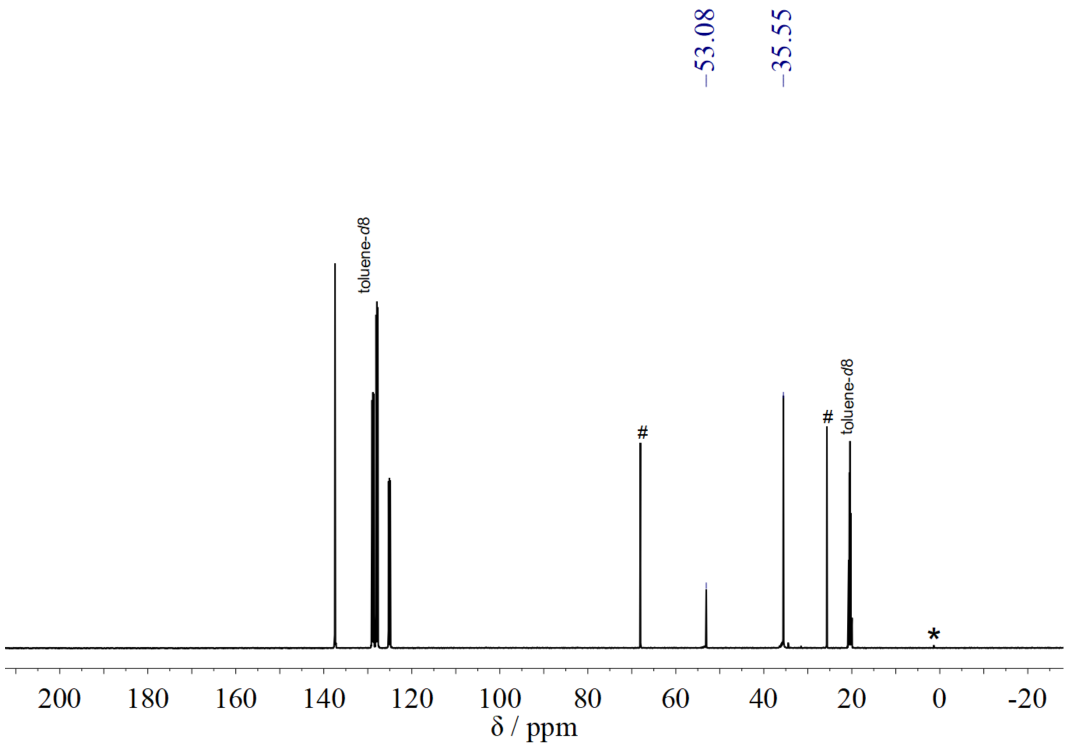

Figure S12. ^{13}C-NMR data for **2** in toluene-d_6 at 298 K. The Residual solvent signals are labeled with # and referred to coordinated thf. The remaining grease is labeled with *.

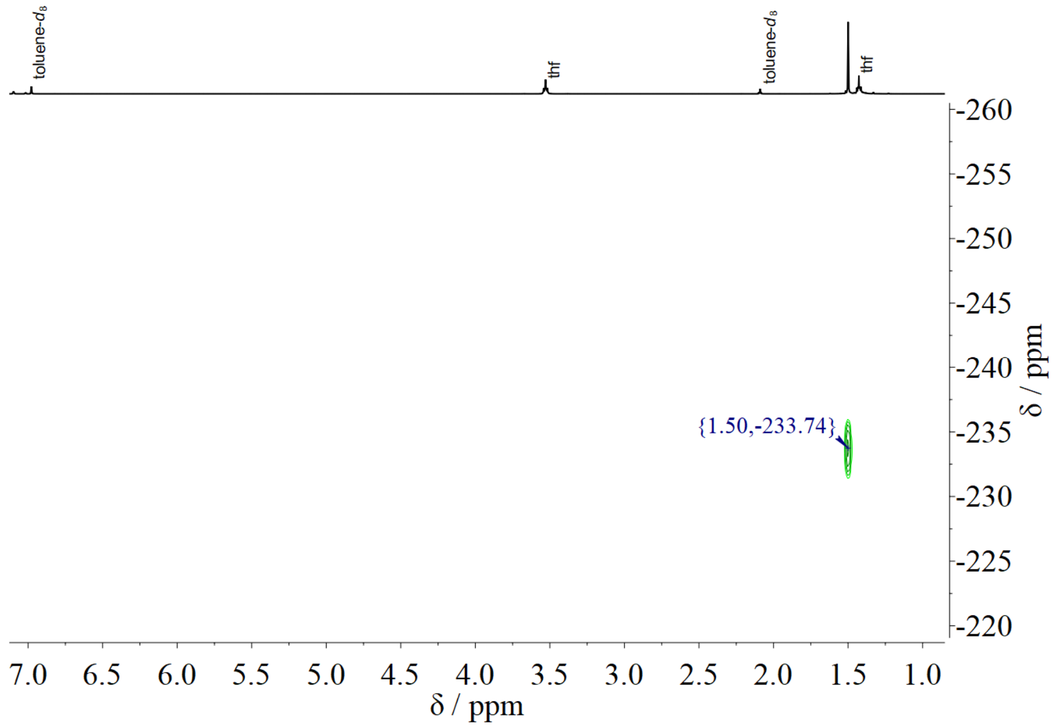

Figure S13. $^{15}N\{H\}$-HMBC-NMR data for **2** in toluene-d_8 at 298 K.

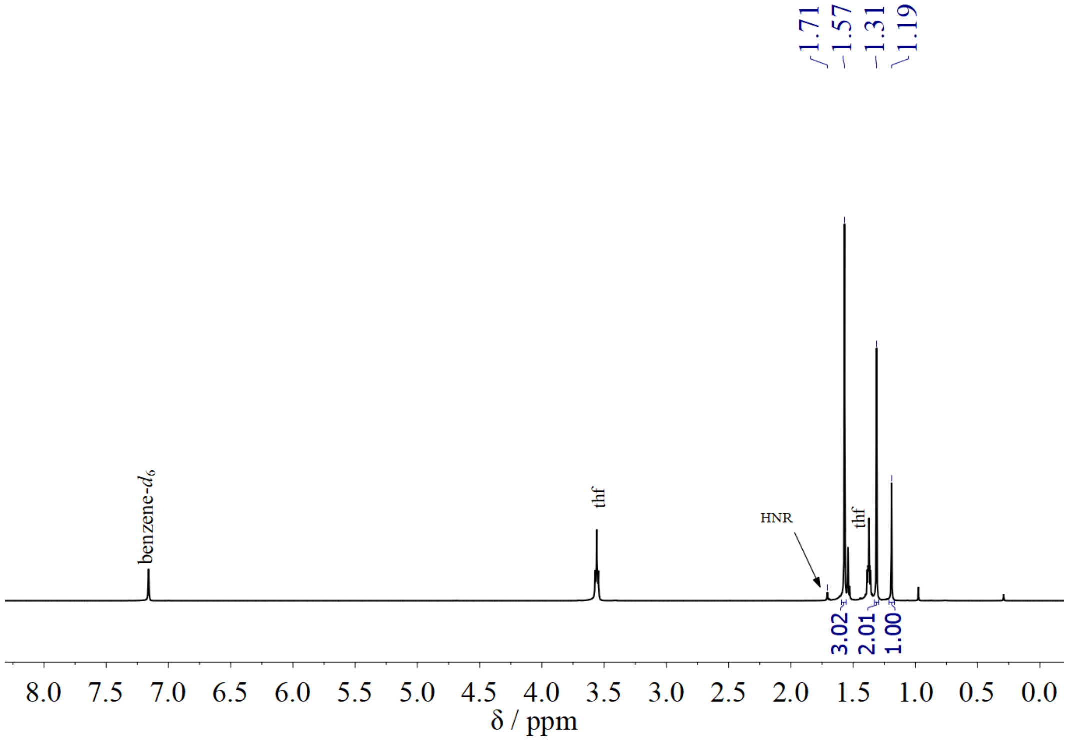

Figure S14. ^1H-NMR data for **3** in benzene-d_6 at 298 K. The residual solvent signals are referred to coordinated thf.

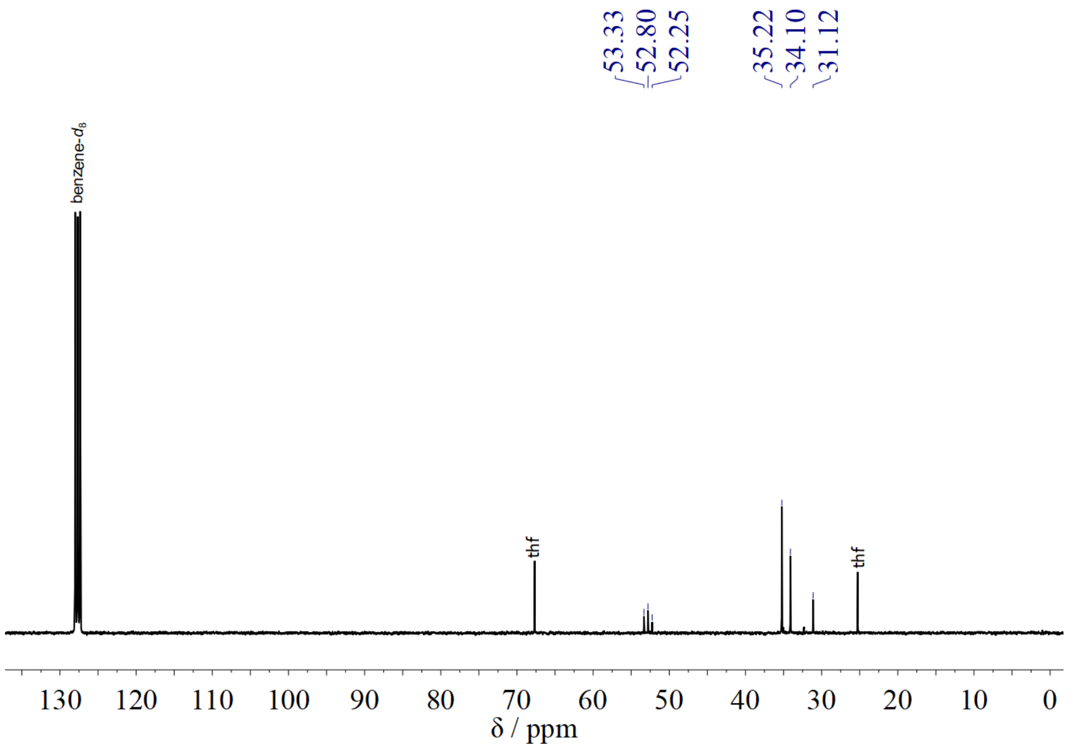

Figure S15. ^{13}C-NMR data for **3** in benzene-d_6 at 298 K. The Residual solvent signals are referred to coordinated thf. The remaining grease is labeled with *.

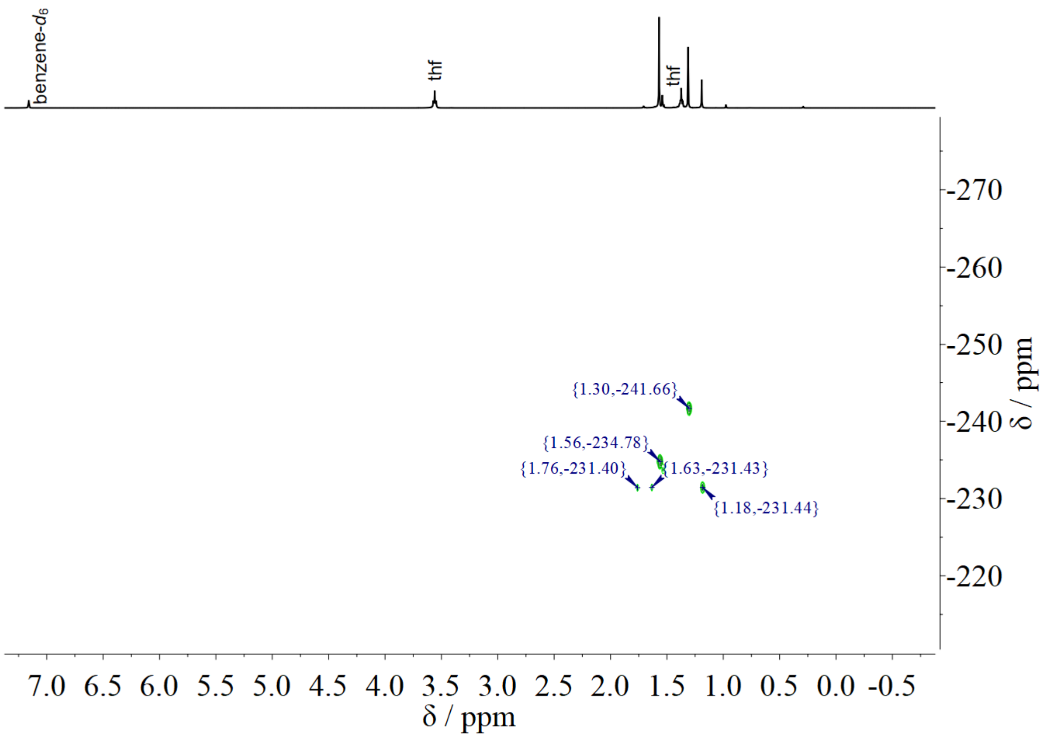

Figure S16. ^{15}N{H}-HMBC-NMR data for **3** in benzene-d_6 at 298 K. The Residual solvent signals are referred to coordinated thf. bis hier Zahlen/Benennung überarbeitet.

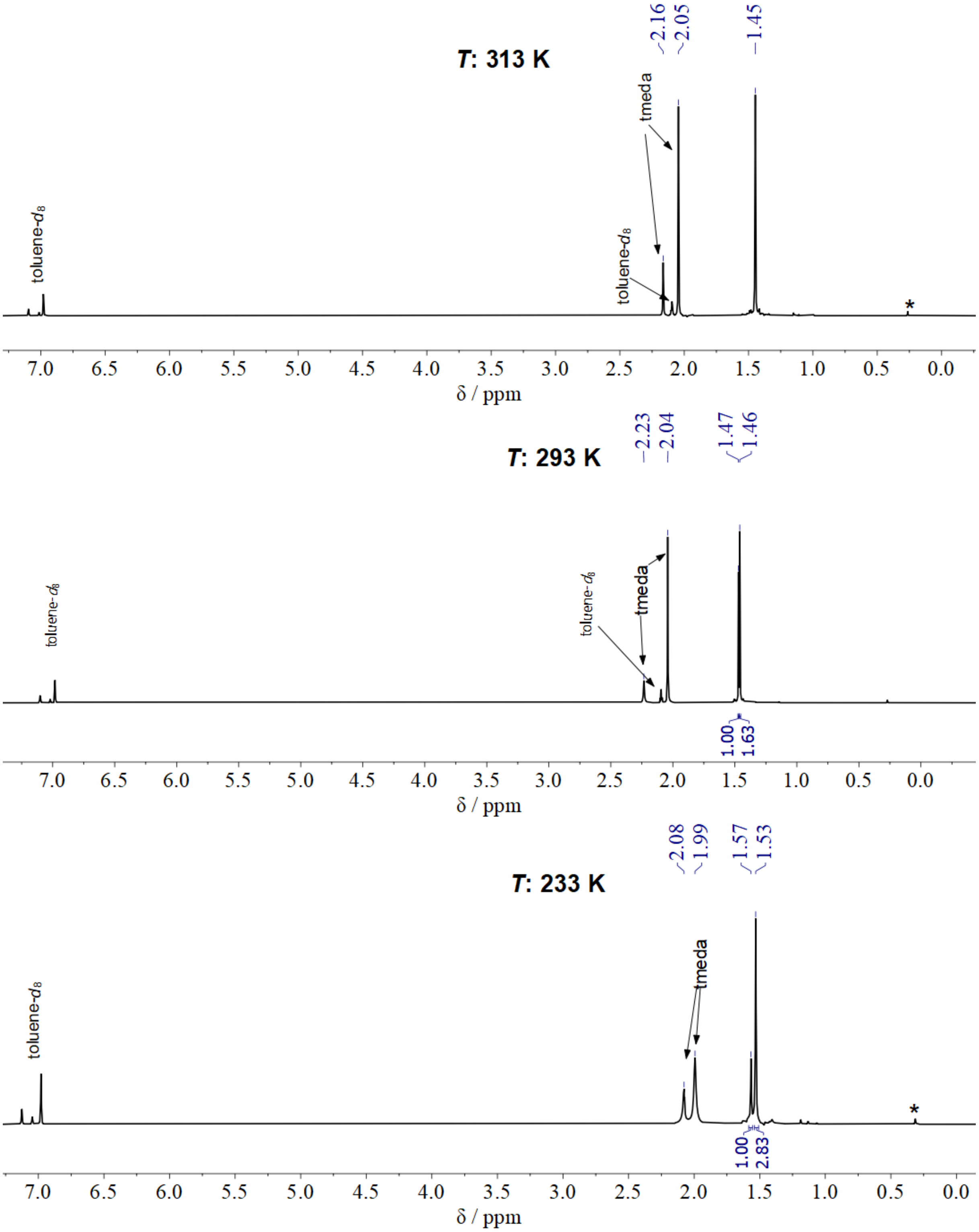

Figure S17. ^{1}H-NMR data for **4** in toluene-*d*$_8$ at 313 K, 293 K, and 233 K, grease is labeled with *. Interestingly, at high temperatures all *t*Bu-groups are equal and giving rise to only one signal. At lower temperatures, there are two signals detected. From 293 K to 233 K the ratio of the integrals varies and suggest a dynamic of coordinated and free tmeda.

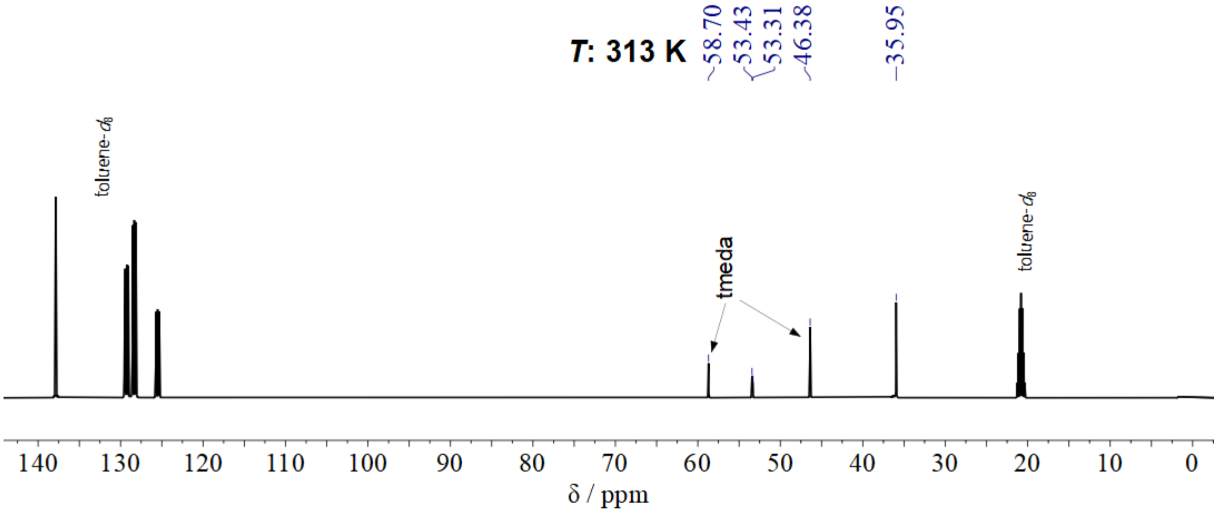

Figure S18. ^{13}C-NMR data for **4** in toluene-d_8 at 293 K.

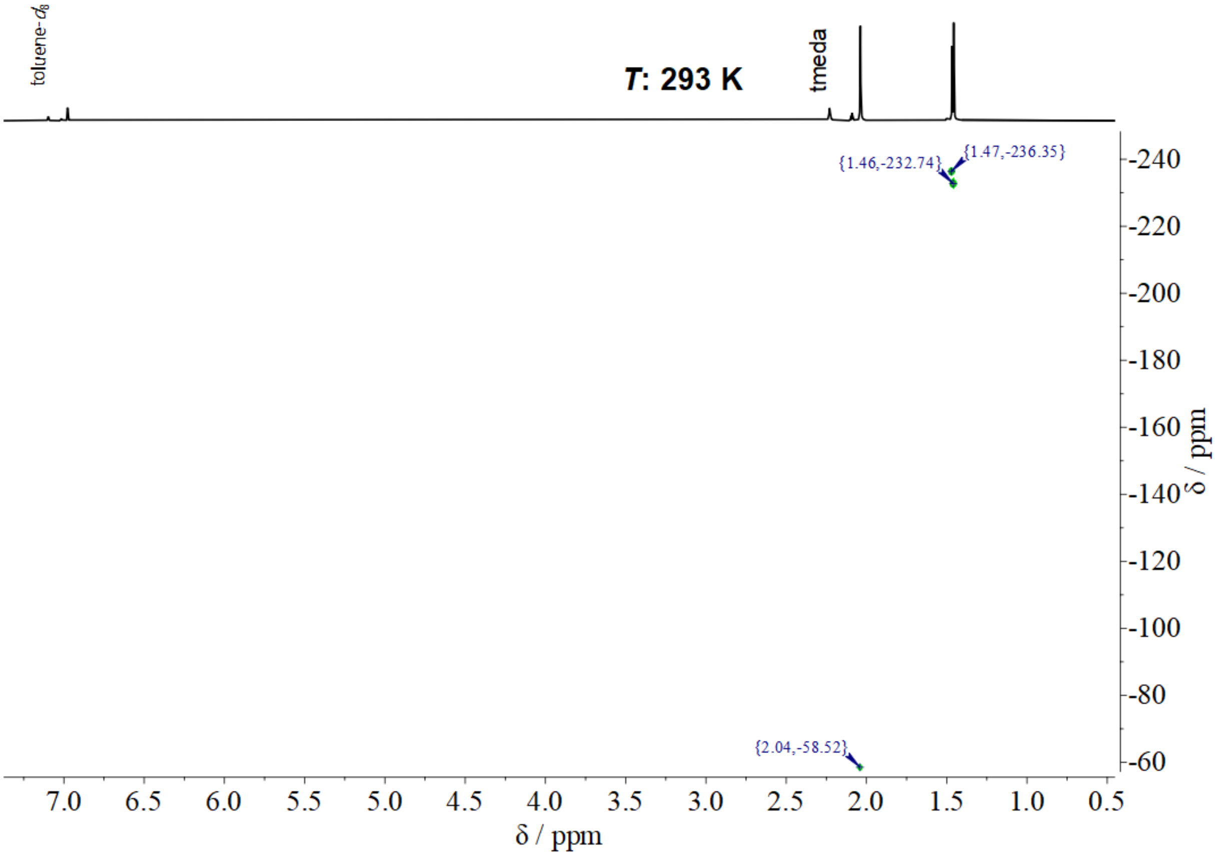

Figure S19. ^{15}N{H}-HMBC-NMR data for **4** in toluene-d_8 at 293 K.

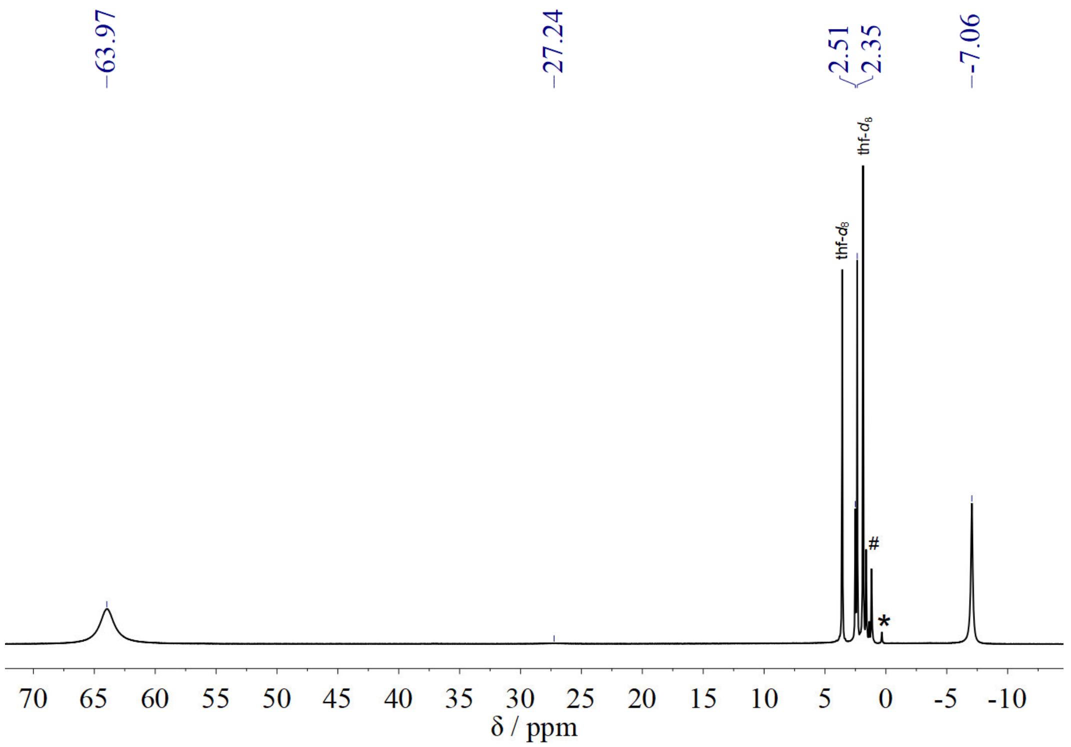

Figure S20. ^{1}H-NMR data for **5** in thf-d_8 at 298 K. The Residual solvent signals are labeled with # and grease is labeled with *.

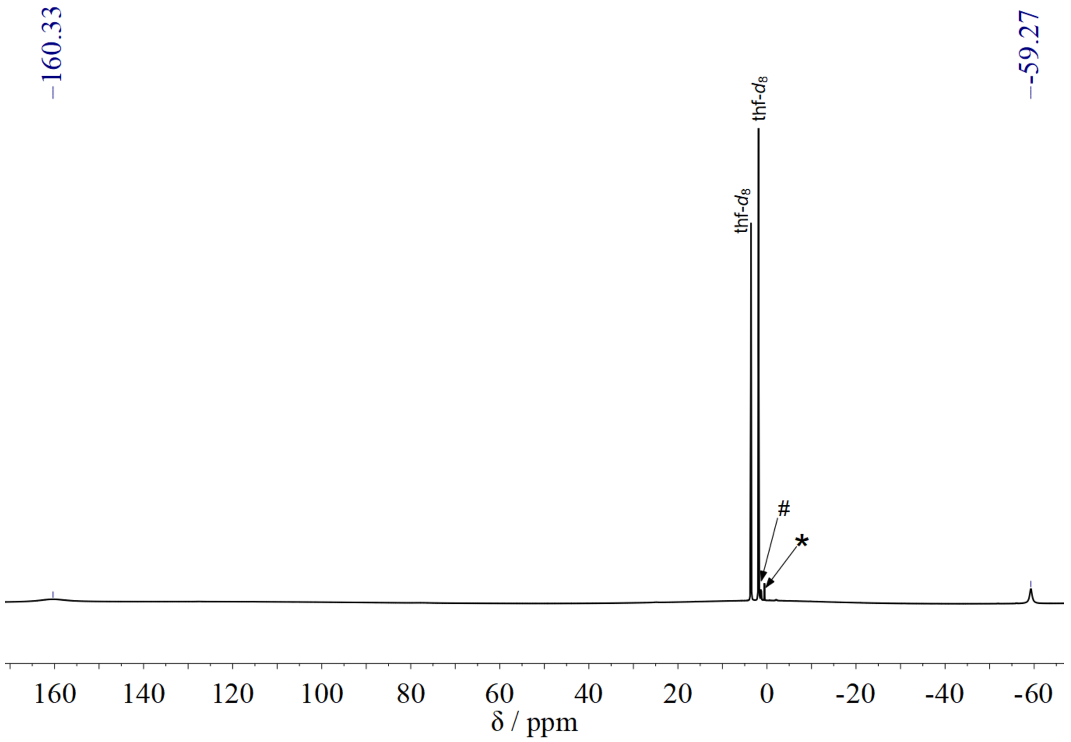

Figure S21. ^{1}H-NMR data for **6** in thf-d_8 at 298 K. The Residual solvent signals are labeled with # and grease is labeled with *.

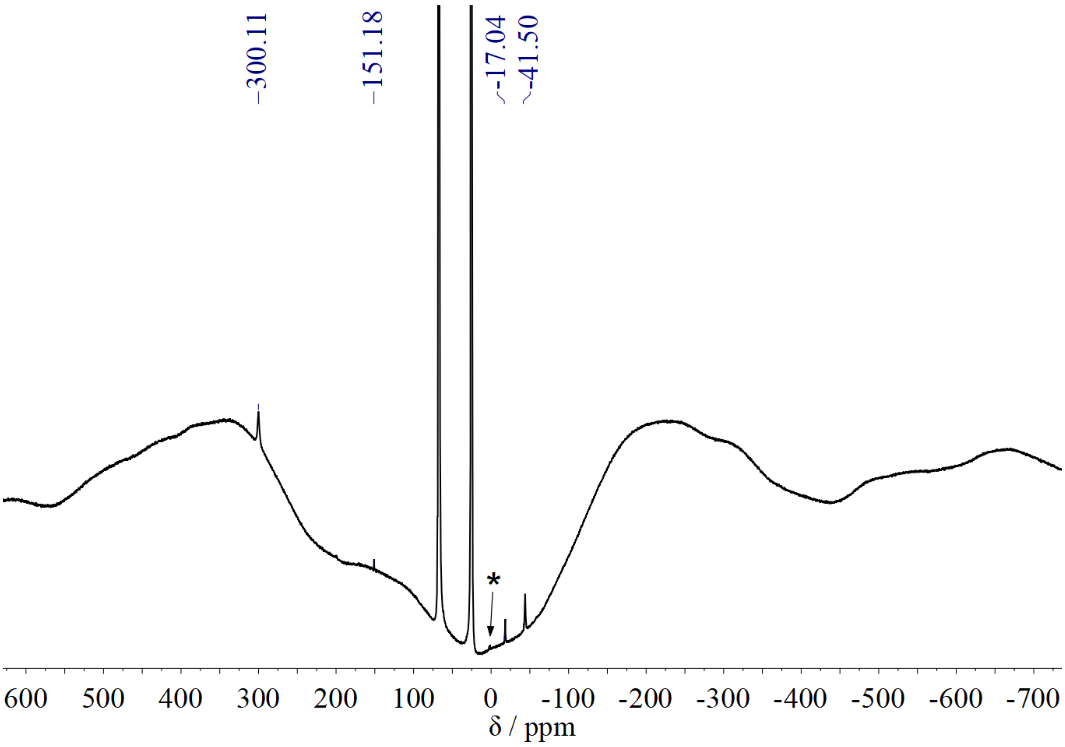

Figure S22. ^{13}C-NMR data for **6** in thf-d_8 at 298 K. The remaining grease is labeled with *.

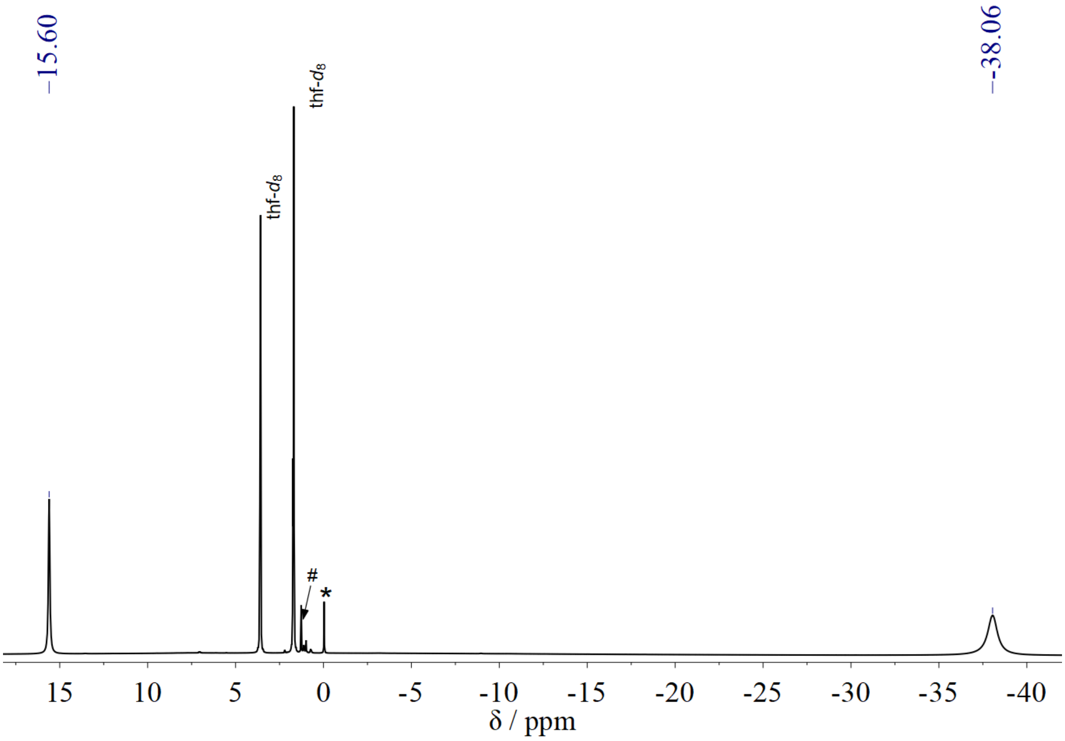

Figure S23. ^{1}H-NMR data for **7** in thf-d_8 at 298 K. The Residual solvent signals are labeled with # and grease is labeled with *.

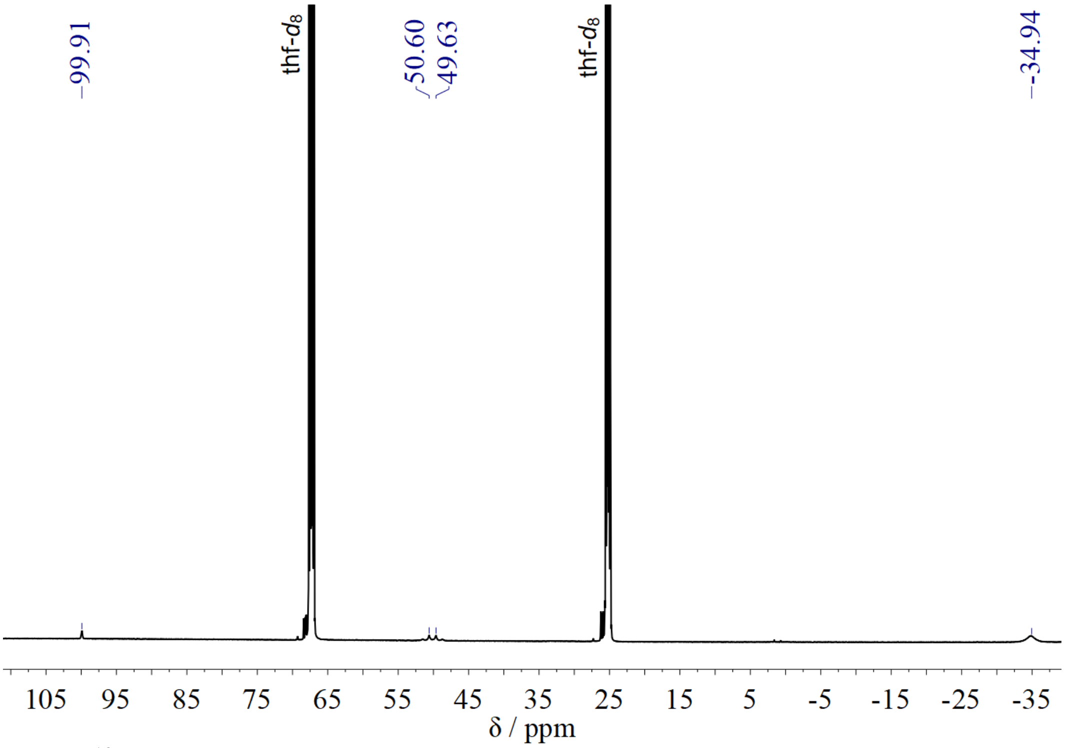

Figure S24. ^{13}C-NMR data for **7** in thf-d_8 at 298 K.

Magnetic data

All magnetic data were recorded on a MPMS-XL-5 or a MPMS 3 magnetometer and processed with the OriginPro 8.5. Samples were prepared in an argon glovebox and the crystalline materials were crushed, covered with Fomblin oil in gelatine caps and inserted in plastic straws. The data were corrected for the diamagnetic contribution of the sample holder according to $M_{dia} = \chi_g \cdot m \cdot H$, with experimentally obtained gram susceptibility of gelatin bucket ($\chi_g = -5.70 \cdot 10^{-7}$ emu/(g·Oe) and of the oil ($\chi_g = -3.51 \cdot 10^{-7}$ emu/(g·Oe)). The molar susceptibility data were corrected for the diamagnetic contribution according to $\chi_{M,dia}$(sample) = $-0.5 \cdot M \cdot 10^{-6}$ $cm^3 \cdot mol^{-1}$.

Ac data:

Occurrence of slow magnetic relaxation was first explored under 0 Oe and 1000 Oe from 2K to 20K or 60K for each complex at maximal frequency (1488 Hz). For maxima in the out-of-phase signal at a temperature *T*, the best optimal field is tested at *T* by screening the applied dc field. Additionally, screening for for SMM behavior, the smallest possible applied magnetic fields are anticipated. Subsequently, ac data was recorded from 2K to *T* at H_{dc} = 100 Oe.

The dependency of the temperature of the received relaxation times for the main process (Orbach process) were processed according to the Arrhenius law applying:

$$\tau = \tau_0 \cdot exp\left(\frac{U_{\text{eff}}}{k_B T}\right) \quad \text{Eq. S1}$$

The full fits for the energy barrier were operated according to the following equation:

$$\tau^{-1} = \tau_0{}^{-1} \cdot e^{-U_{\text{eff}}/k_B T} + CT^n + \tau_{\text{QTM}}^{-1} \quad \text{Eq. S2}$$

taking Orbach, Raman processes and, if necessary, QTM into account.

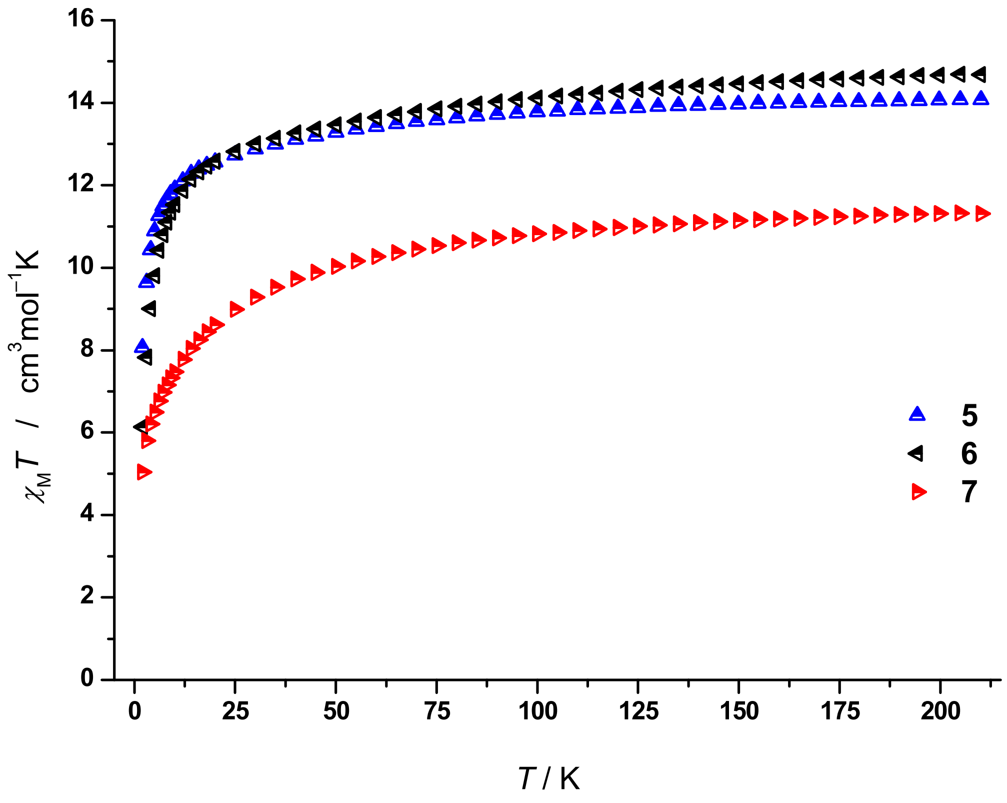

Figure S25. $\chi_M T$ plotted versus the temperature for **5** to **7**.

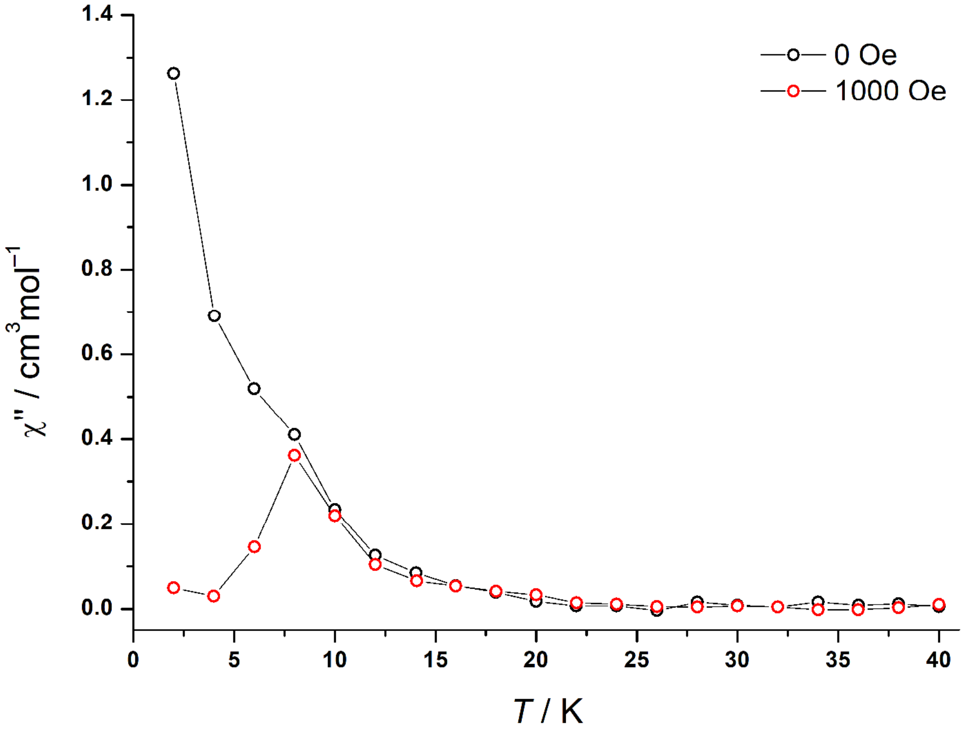

Figure S26. χ'' versus T for **5** with 0 Oe and 1000 Oe applied dc field.

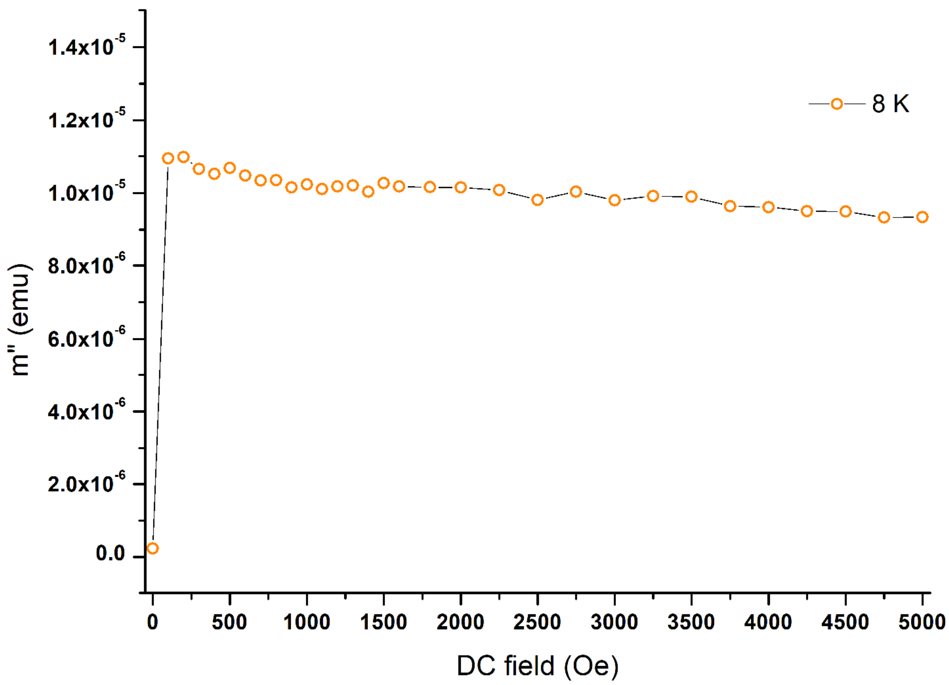

Figure S27. Determination of the optimal dc field: *m*'' versus dc field for **5** with various fields at 8 K, which is the highest temperature were SMM behavior is present under an applied field. A field of 100 Oe was chosen.

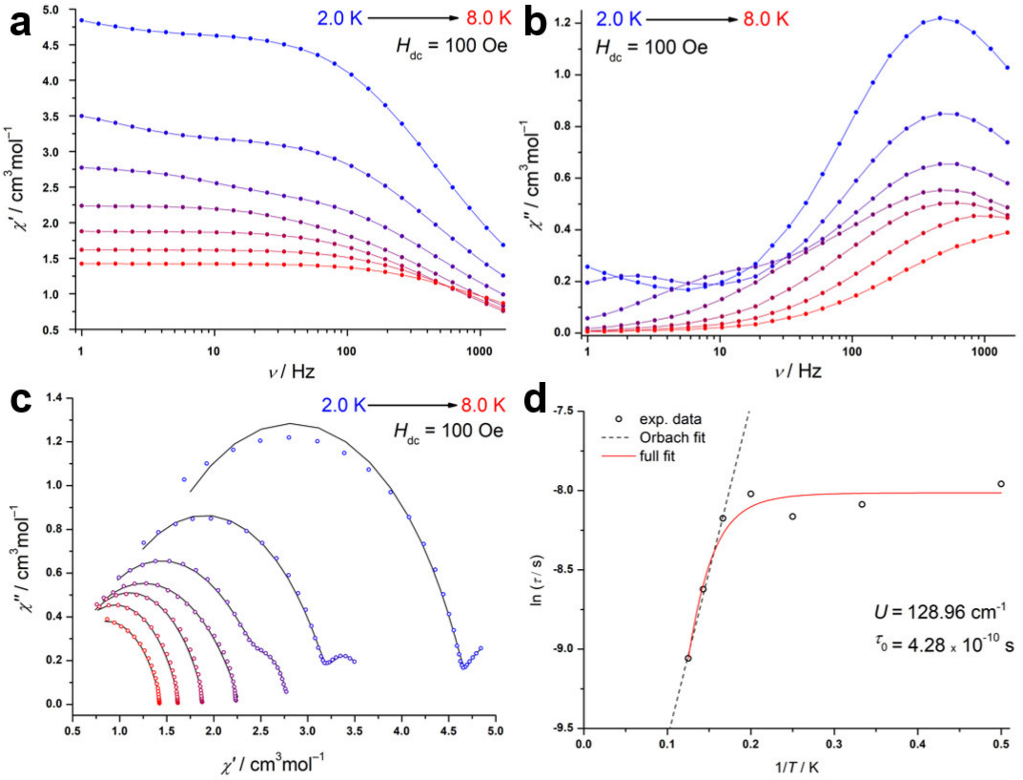

Figure S28. Magnetic data for **5** under an applied field of 100 Oe. Dynamic in-phase (**a**) and out-of-phase (**b**) susceptibility versus variable frequencies from 2 to 8 K. Cole-Cole plot (**c**) with corresponding CC-fit data; Arrhenius plot (**d**) with red curve exemplifying the full fit of all relaxation processes and the dash line demonstrates a sole Orbach regression.

Furthermore, it is confirmed in the unexpected shape of the curves, mainly visible at low temperatures from 2 to 4 K in the Cole-Cole plot (Figure S28c). Therefore, two relaxation processes were applied within the CC-fit program to generate the Cole-Cole plot fit and the corresponding data for the Arrhenius plot (Figure S28d). A linear fit of the Arrhenius plot unveiled a slightly higher effective energy barrier of 21.1 cm^{-1} employing only the Orbach relaxation process (dashed line, Figure 28d).

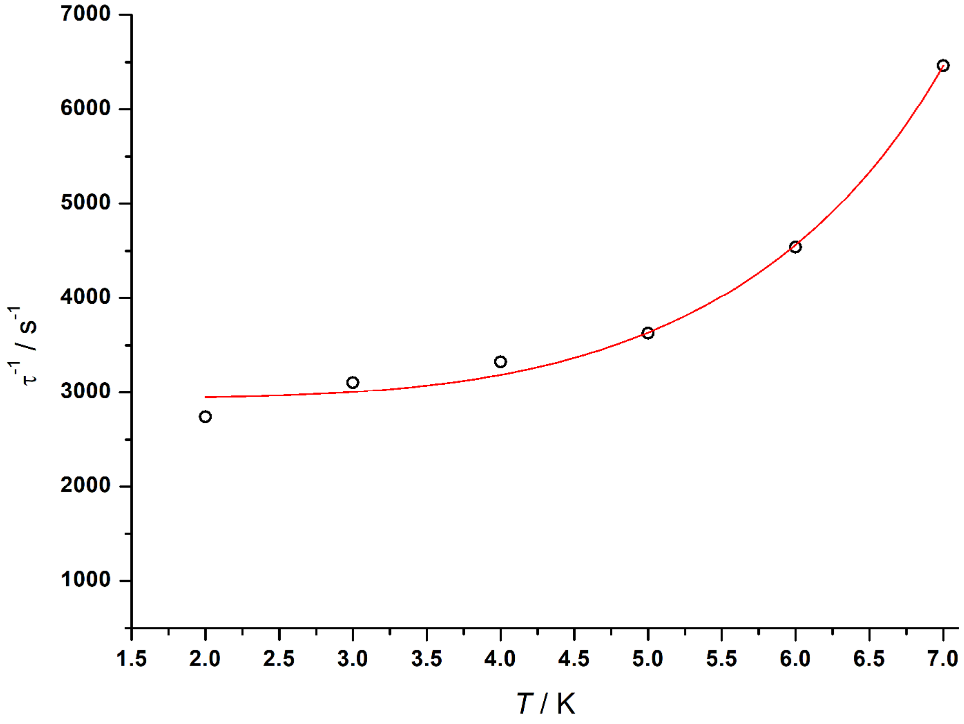

Figure S29. Relaxation curve for **5** with zero field conditions. The red line represents the relaxation fit according to **Eq 2**.

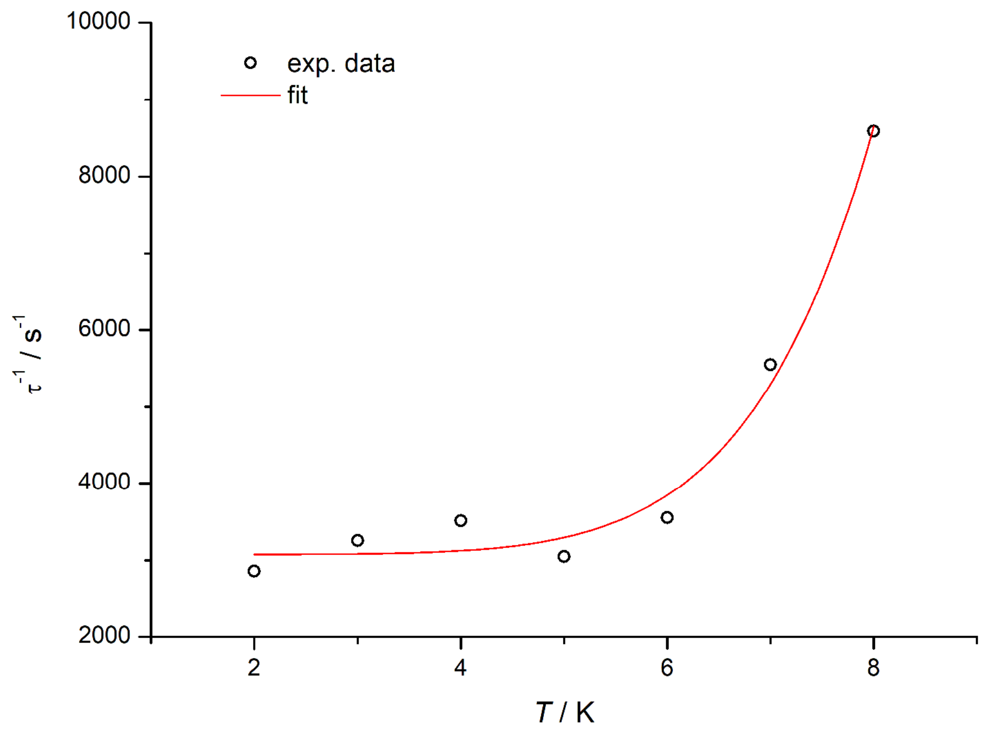

Figure S30. Relaxation curve for **5** with 100 Oe conditions. The red line represents the relaxation fit according to **Eq 2**.

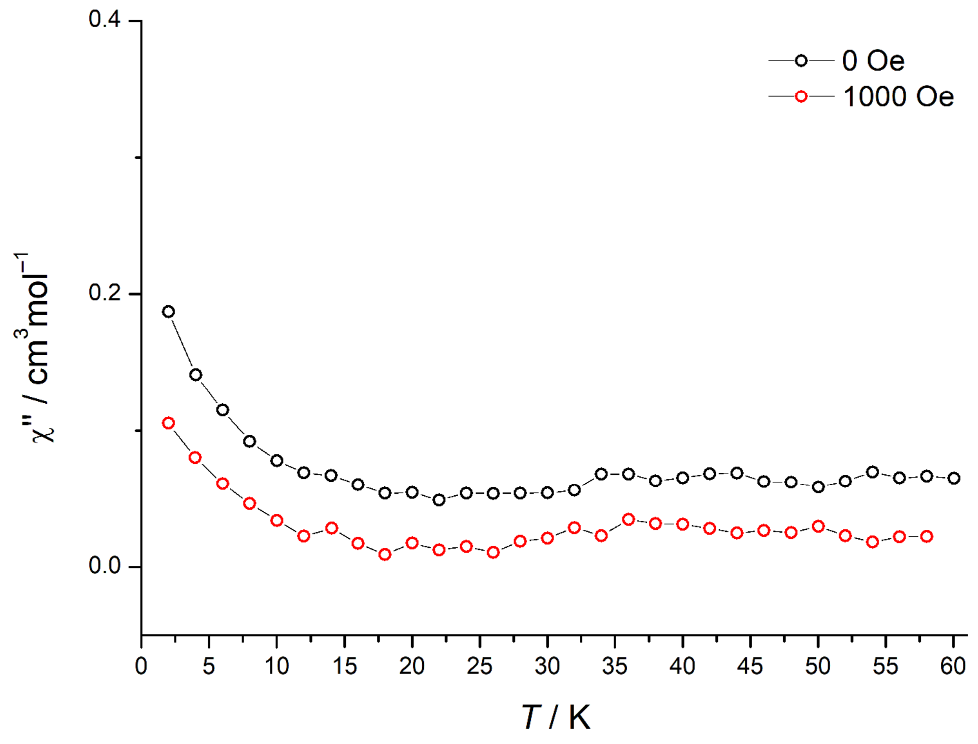

Figure S31. χ'' versus T for **6** with 0Oe and 1000Oe applied field, for the highest frequency of 1488 Hz. No Maxima found since the maxima are positioned at lower frequencies as illustrated in Figure S32. Under an applied field, no maxima were observed.

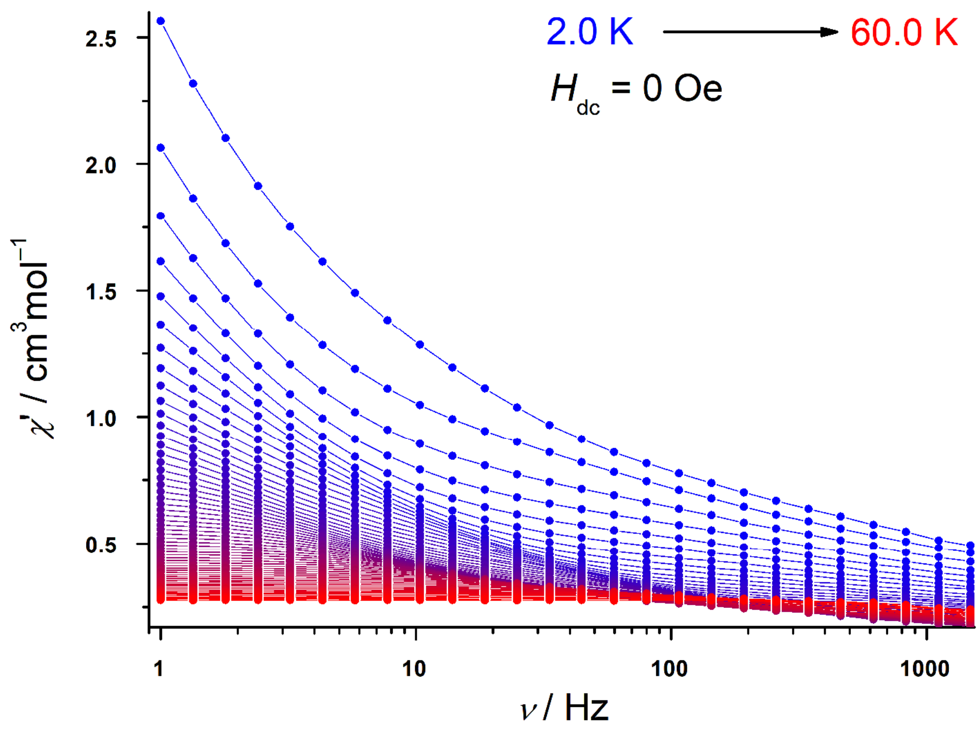

Figure S32. χ' versus v for **6** with 0Oe conditions from 2 K to 60 K.

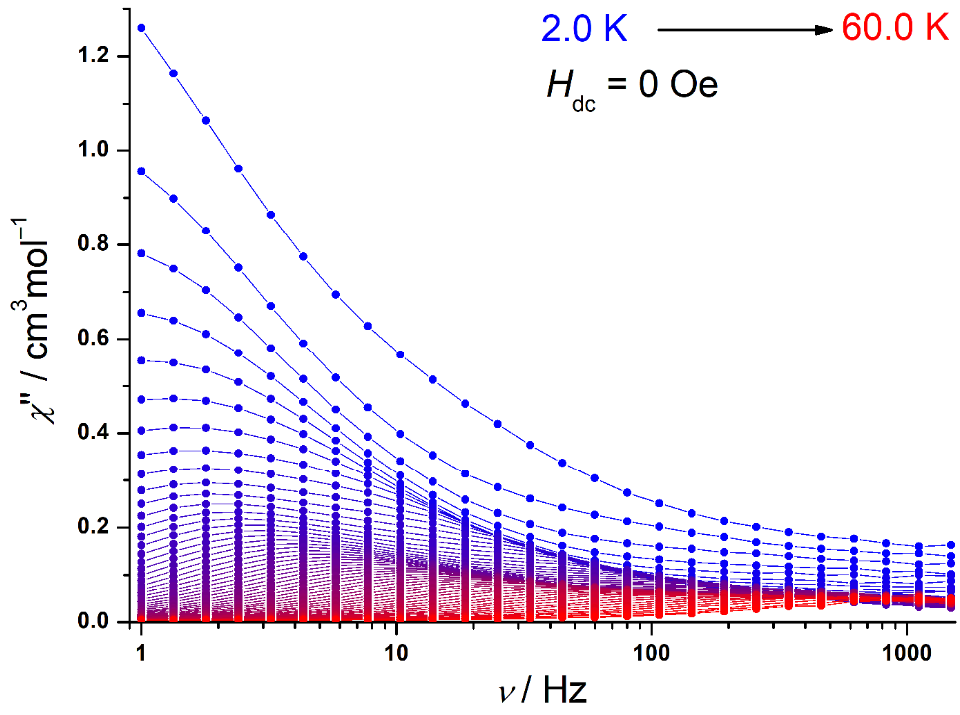

Figure S33. χ' versus v for **6** with 0Oe conditions from 10 K to 60 K.

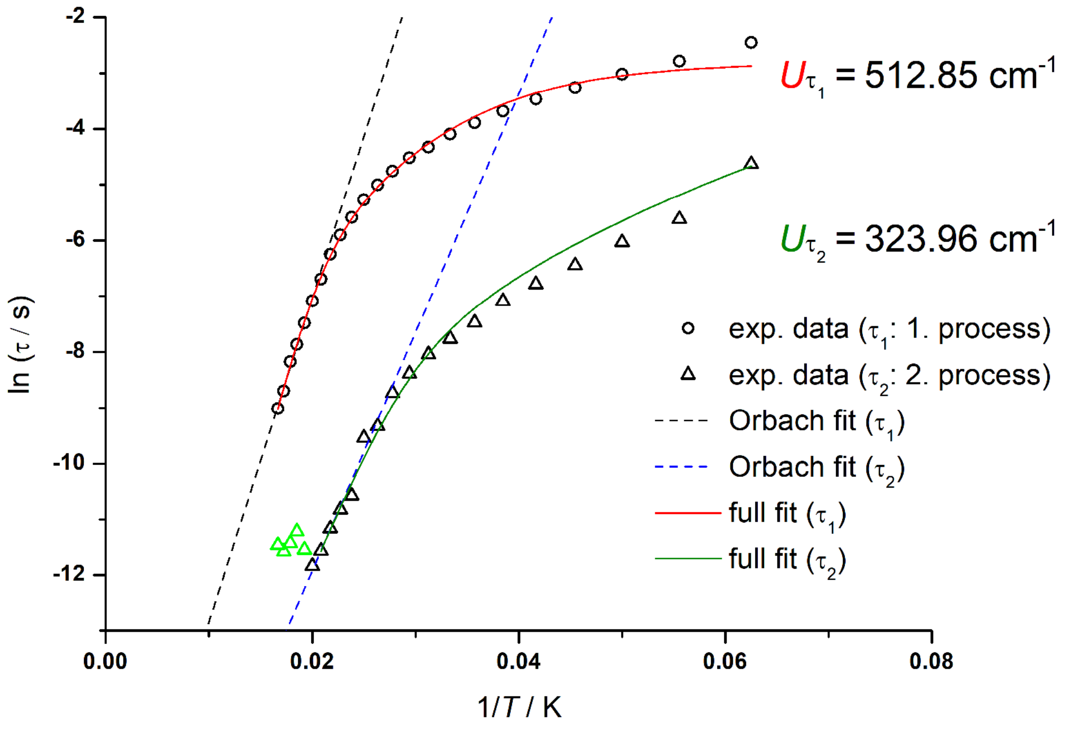

Figure S34. Arrhenius plot for **6** with the full fit of both relaxation processes. For the second process tau 2, the green triangles were excluded for the full fit.

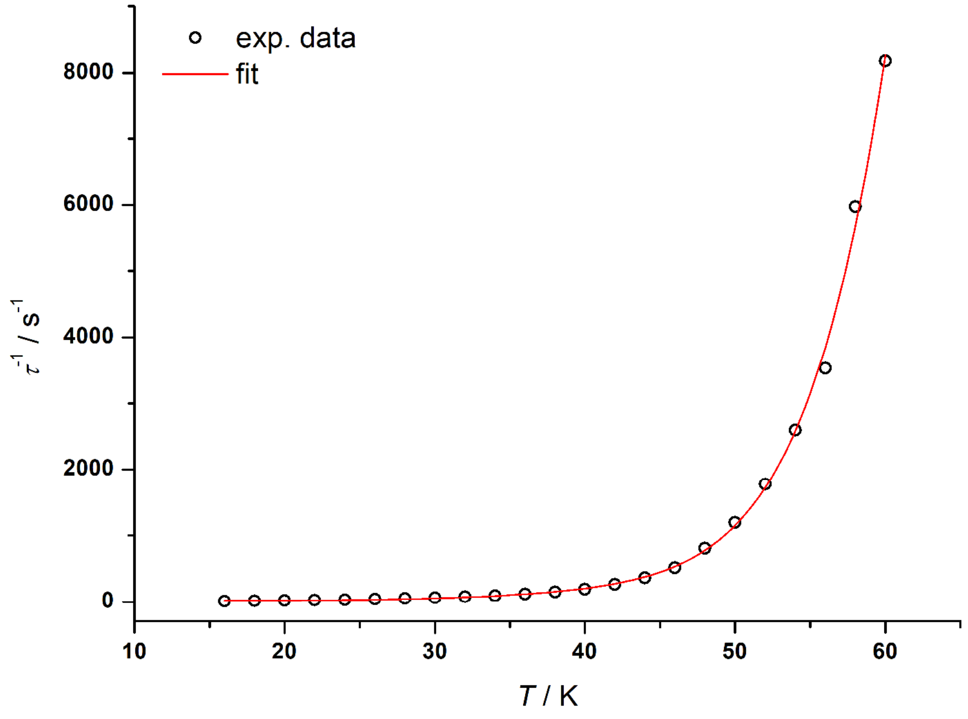

Figure S35. Relaxation curve for **6** under zero field conditions for tau1. The red line represents the relaxation fit according to **Eq 2**.

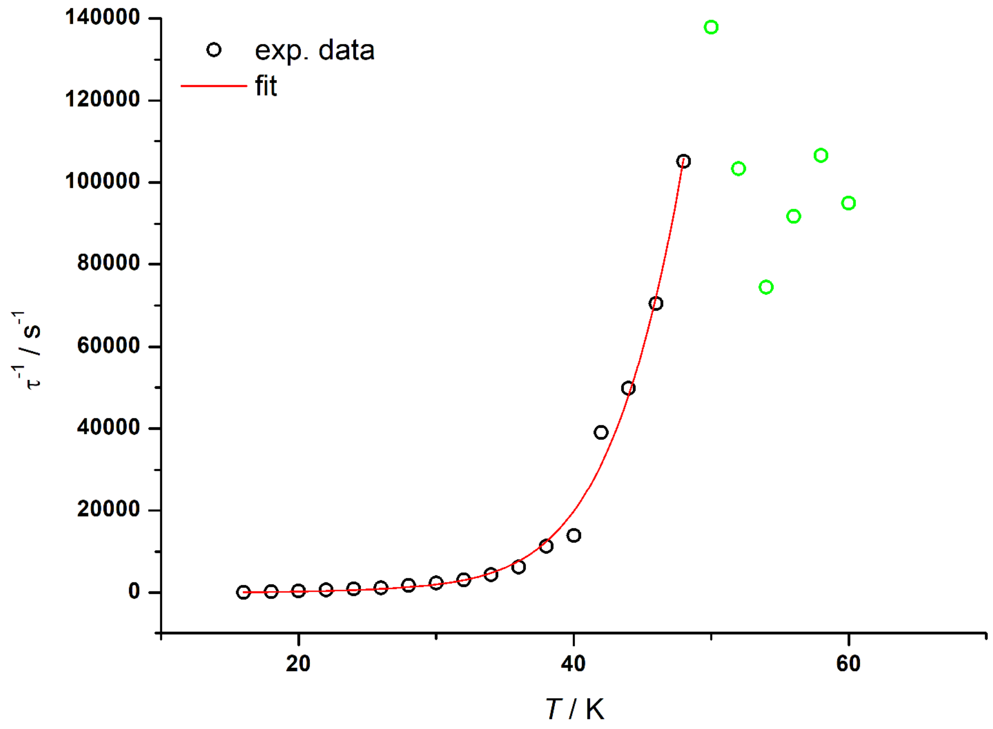

Figure S36. Relaxation curve for **6** under zero field conditions for tau2. The red line represents the relaxation fit according to **Eq 2**. The green data was exclude from the full fit.

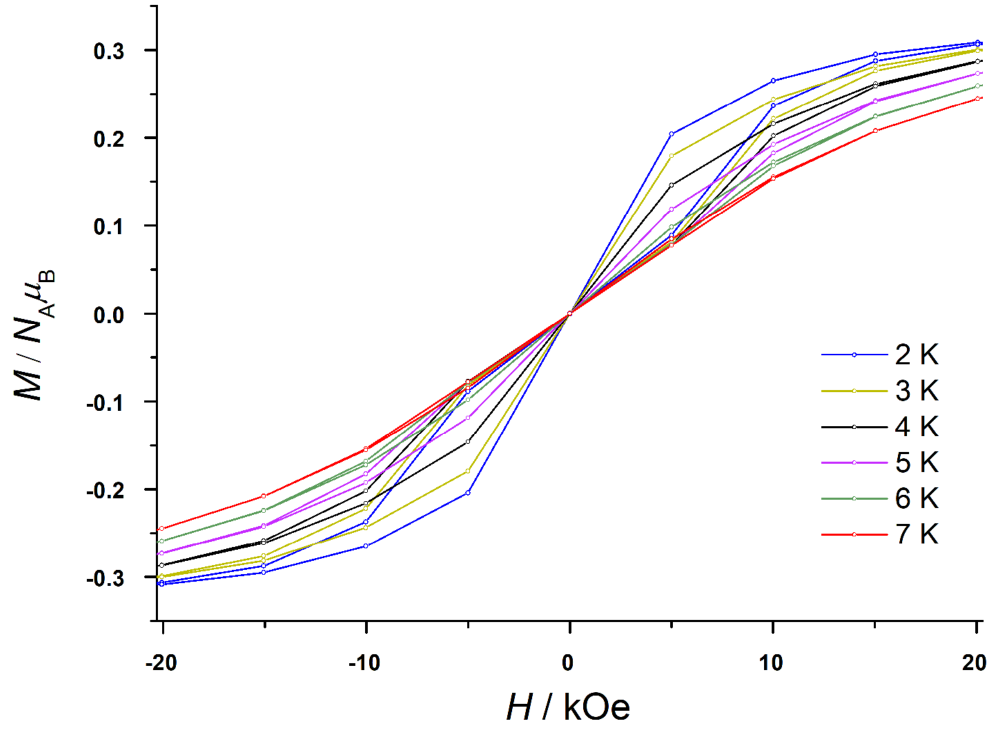

Figure S37. Field dependency of the magnetization for **6** from 2 K to 7 K at a sweep rate of 185 Oe/s.

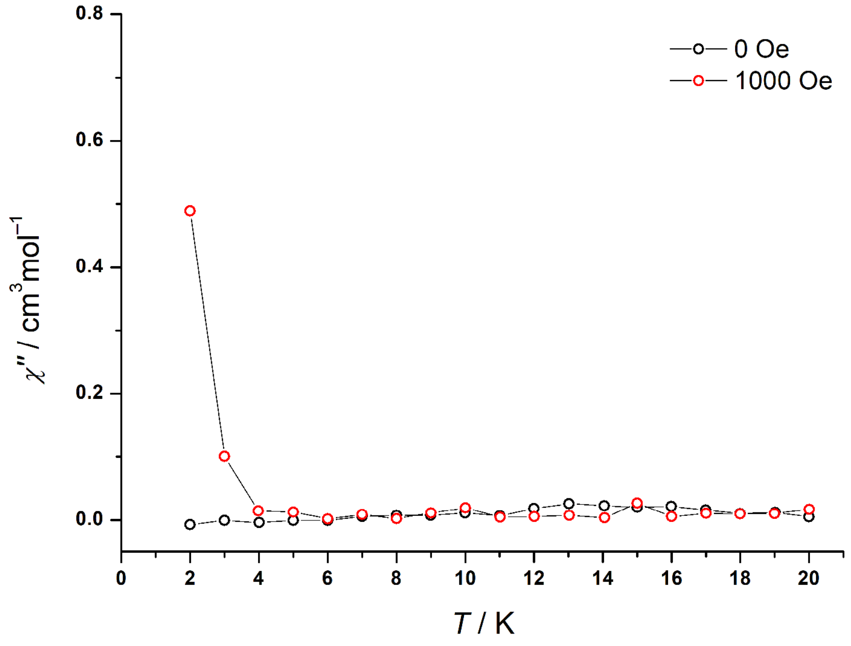

Figure S38. χ'' versus T for **7** with 0Oe and 1000Oe applied field.

The full fits of the relaxation time according to **Eq S2** give the following best fitting parameters:

Table S10: Best fitting parameters for **5**.

#	**5** (0 Oe)	**5** (100 Oe)
τ_0 (s^{-1})	$2.01 \cdot 10^{-9}$ ($\pm 8.09 \cdot 10^{-10}$)	$4.28 \cdot 10^{-10}$ ($\pm 7.95 \cdot 10^{-12}$)
$U_{eff,\ fullfit}$ (cm^{-1})	77.04.0 (±8.31)	128.97 (±14.89)
$U_{eff,\ Orbach}$ (cm^{-1})	14.9 (±3.32)	21.1 (±4.89)
N	4.59 (±0.39)	6.89 (±0.13)
C ($s^{-1}K^{-n}$)	0.43 (±0.06)	$3.37 \cdot 10^{-3}$ ($\pm 1.52 \cdot 10^{-4}$)
τ_{QTM}^{-1} (s^{-1})	2937.45 (±95.47)	3075.94 (±176.23)

Table S11: Best fitting parameters for **6.**

#	**6** (0 Oe, τ_1)	**6** (0 Oe, τ_2)
τ_0 (s^{-1})	$6.66 \cdot 10^{-10}$ ($\pm 2.22 \cdot 10^{-11}$)	$8.25 \cdot 10^{-10}$ ($\pm 1.32 \cdot 10^{-12}$)
U_{eff} (cm^{-1})	512.85 (±74.85)	316.34 (±3.02)
$U_{eff,\ Orbach}$ (cm^{-1})	403.26 (±17.86)	297.20 (±27.93)
n	5.17 (±0.35)	4.41 (±0.14)
C ($s^{-1}K^{-n}$)	$1.50\ 10^{-3}$ ($\pm 1.50 \cdot 10^{-4}$)	$5.22 \cdot 10^{-4}$ ($\pm 4.20 \cdot 10^{-5}$)
τ_{QTM}^{-1} (s^{-1})	1680.13 (±15.0)	0 (±36.1)

[1] T. Schulz, K. Meindl, D. Leusser, D. Stern, J. Graf, C. Michaelsen, M. Ruf, G. M. Sheldrick, D. Stalke, *J. Appl. Crystallogr.* **2009**, *42*, 885-891.
[2] Bruker AXS Inc., in *Bruker Apex CCD, SAINT v8.30C* (Ed.: Bruker AXS Inst. Inc.), WI, USA, Madison, 2013.
[3] L. Krause, R. Herbst-Irmer, G. M. Sheldrick, D. Stalke, *J. Appl. Crystallogr*. **2015**, *48*, 3-10.
[4] G. M. Sheldrick, *Acta Crystallogr.* **2015**, *A71*, 3-8.
[5] G. M. Sheldrick, *Acta Crystallogr.* **2015**, *C71*, 3-8.
[6] C. B. Hübschle, G. M. Sheldrick, B. Dittrich, *J. Appl. Crystallogr.* **2011**, *44*, 1281-1284.
